# Probabilistic Learning by Rodent Grid Cells

**DOI:** 10.1371/journal.pcbi.1005165

**Published:** 2016-10-28

**Authors:** Allen Cheung

**Affiliations:** The University of Queensland, Queensland Brain Institute, Upland Road, St. Lucia, Queensland, Australia; University College London, UNITED KINGDOM

## Abstract

Mounting evidence shows mammalian brains are probabilistic computers, but the specific cells involved remain elusive. Parallel research suggests that grid cells of the mammalian hippocampal formation are fundamental to spatial cognition but their diverse response properties still defy explanation. No plausible model exists which explains stable grids in darkness for twenty minutes or longer, despite being one of the first results ever published on grid cells. Similarly, no current explanation can tie together grid fragmentation and grid rescaling, which show very different forms of flexibility in grid responses when the environment is varied. Other properties such as attractor dynamics and grid anisotropy seem to be at odds with one another unless additional properties are assumed such as a varying velocity gain. Modelling efforts have largely ignored the breadth of response patterns, while also failing to account for the disastrous effects of sensory noise during spatial learning and recall, especially in darkness. Here, published electrophysiological evidence from a range of experiments are reinterpreted using a novel probabilistic learning model, which shows that grid cell responses are accurately predicted by a probabilistic learning process. Diverse response properties of probabilistic grid cells are statistically indistinguishable from rat grid cells across key manipulations. A simple coherent set of probabilistic computations explains stable grid fields in darkness, partial grid rescaling in resized arenas, low-dimensional attractor grid cell dynamics, and grid fragmentation in hairpin mazes. The same computations also reconcile oscillatory dynamics at the single cell level with attractor dynamics at the cell ensemble level. Additionally, a clear functional role for boundary cells is proposed for spatial learning. These findings provide a parsimonious and unified explanation of grid cell function, and implicate grid cells as an accessible neuronal population readout of a set of probabilistic spatial computations.

## Author Summary

Cells in the mammalian hippocampal formation are thought to be central for spatial learning and stable spatial representations. Of the known spatial cells, grid cells form strikingly regular and stable patterns of activity, even in darkness. Hence, grid cells may provide the universal metric upon which spatial cognition is based. However, a more fundamental problem is how grids themselves may form and stabilise, since sensory information is noisy and can vary tremendously with environmental conditions. Furthermore, the same grid cell can display substantially different yet stable patterns of activity in different environments. Currently, no model explains how vastly different sensory cues can give rise to the diverse but stable grid patterns. Here, a new probabilistic model is proposed which combines information encoded by grid cells and boundary cells. This noise-tolerant model performs robust spatial learning, under a variety of conditions, and produces varied yet stable grid cell response patterns like rodent grid cells. Across numerous experimental manipulations, rodent and probabilistic grid cell responses are similar or even statistically indistinguishable. These results complement a growing body of evidence suggesting that mammalian brains are inherently probabilistic, and suggest for the first time that grid cells may be involved.

## Introduction

Mammals use probabilistic computations to perceive noisy and ambiguous sensory inputs [1–5]. It seems likely that learning an internal model of a noisy sensory environment should follow similar statistical inference principles [4]. While solid behavioural evidence [1–5] and mounting *in vivo* evidence [3, 4] support probabilistic sensory perception, *in vivo* evidence is lacking for probabilistic learning [4, 5]. It is virtually unknown how any probabilistically learned neural model of the world may look through neurophysiological recordings.

The mammalian hippocampal formation is heavily implicated in spatial learning [6–9]. Grid cells within the hippocampal formation tile Euclidean space in a repeating firing pattern, thought to provide a spatial metric [7–11]. Both theoretical and experimental evidence suggest that grid cells may be used for path integration (PI) via integration of self-motion estimates [8, 10, 12–14]. However, all PI systems suffer from cumulative error [15, 16] necessitating frequent corrections [17–22]. In darkness [10, 13, 23, 24], fusion of sensory and learned information is necessary to maintain spatially-stable grid cell responses [17, 18, 25]. Theoretically, learned boundary information is sufficient to correct cumulative PI errors in darkness [17, 18]. Consistent with theory, boundary cells have been found to fire along arena boundaries [26–29], coexist with grid cells in the hippocampal formation, and provide a plausible neuronal substrate to encode boundary information [17–20]. However, it is unclear how grid and boundary information contribute to spatial learning, or how their responses may be altered by learning.

Currently, no realistic learning model can unify grid and boundary cell activity for learning or localization in light and dark conditions. Darkness poses a formidable challenge by limiting inputs to noisy self-motion and intermittent boundary contacts, neither being location-specific. The approach of approximating spatial learning by assuming error-free PI by grid cells [20, 22] bypasses the fundamental problem of SLAM (simultaneous localization and mapping) [30–32], overlooking how cumulative errors [15, 16] impair spatial learning and undoubtedly shaped the evolution of spatial cognition. Spatial learning models which rely on vision [21, 33] do not generalize to explain stable grid fields in darkness [10, 13]. Additionally, the nature of learned spatial information must affect the diverse grid cell responses caused by arena manipulations, including grid rescaling [34, 35] and grid fragmentation [36, 37], but whose relationship to learned spatial representations remain unclear. Here, a new learning model adapted from probabilistic SLAM [30–32] is proposed which explains how information encoded by grid and boundary cell populations may be simultaneously learned, recalled and stabilized. This model takes realistic noisy inputs, represented by plausible neuronal codes using rate-coded (non-spiking) grid and boundary cell models, and carries out probabilistic information fusion algorithmically. Grid and boundary cell responses are modulated recursively through information fusion, during both learning and recall. Expected neuronal responses are characterized and compared to rodent electrophysiological recordings across diverse conditions.

## Results

### Probabilistic learning using grid and boundary cells

**Fig 1:**
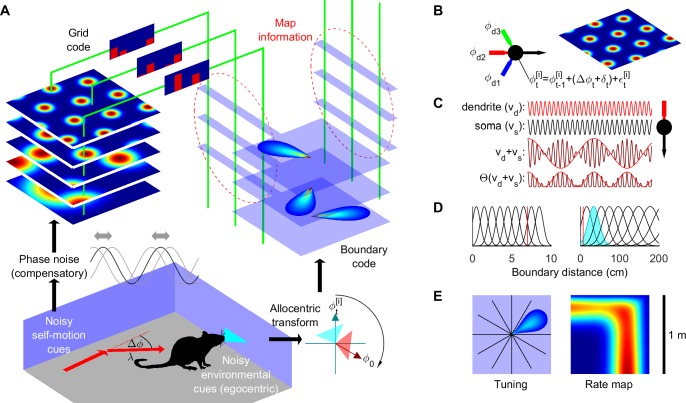
Probabilistic spatial learning using grid and boundary cells. (**A**) Self-motion (angular and linear displacement, Δ*ϕ* and λ) and boundary inputs (transformed via estimated heading, $\phi_{t}^{\left[i\right]}$) shift grid phase and activate boundary cells, respectively, forming associative maps (dashed ellipses) during learning. (**B**) Self-motion phase signal is integrated along each preferred direction of the grid cell (*ϕ*_d1_, *ϕ*_d2_, *ϕ*_d3_) via plane wave oscillators, whose coincident activity leads to a hexagonal tessellating spatial grid in the absence of noise. (**C**) An example of plane wave oscillators produced by the interference between dendritic membrane oscillations, *v*_*d*_ = cos(*ω*_*d*_*t*), and somatic membrane oscillations, *v*_*s*_ = cos(*ω*_*s*_*t*), producing an approximately sinusoidal rectified oscillation, Θ(*v*_*d*_ + *v*_*s*_), where Θ denotes the Heaviside function [14]. (**D**) Distance tuning functions of short-range (left) and long-range (right) boundary cells (red line—maximum boundary detection distance without vision). (**E**) Firing rate map (right) of boundary cell with example tuning direction (**E**, left) and distance (**D**, shaded). See also S1 Fig and S1.1 Text.

A probabilistic grid and boundary cell spatial information fusion model (SIFM) was developed (Figs 1A and S1, Methods, S1.1 Text) based on a Rao-Blackwellized particle-filter [30–32]. This algorithmic implementation of information fusion continually updates a sample of possible positions, orientations, and environmental layouts (maps), primarily using self-motion and boundary information. During a learning session, boundary information is associated with position information incrementally to build a distribution of possible maps corresponding to the possible paths defined by the dynamic distribution of positions and orientations. At each step boundary prediction error feeds back to modulate the distribution of positions, orientations and maps.

SIFM is the first demonstration that information contained in grid and boundary codes of the rodent hippocampal formation may be sufficient to implement recursive probabilistic information fusion. In SIFM, noisy self-motion cues update grid cell responses (Fig 1B and 1C, S1.1.2 Text), encoding a distribution of position and orientation (pose), spanning eight modular grid scales estimated from rodent data [35]. Noisy boundary cues are encoded by boundary cells covering twelve tuning directions and nineteen tuning distances (Fig 1D and 1E). Eleven long-range boundary cell tuning distances were inferred previously from hippocampal place cell data [38], while eight additional short-range tuning distances enable boundary detection in darkness, consistent with medial entorhinal boundary cell properties [26]. From self-motion and boundary cues experienced along random trajectories, each multi-modular grid code learns an association map (weights to boundary cells, Figs 1A and S1, S1.1.6 Text), which together determines predictive boundary cell responses. Each multi-modular grid code is assumed to maintain the same relative phase across grid scales (modules) despite continual changes in position. Note that although a self-corrective mechanism could potentially maintain relative grid phase across modules [39], partial decoupling and hence independent operation of some grid modules [35] does not prohibit SIFM function (described later under ‘Three novel tests of probabilistic learning’). During learning and recall, boundary prediction error recursively modulates the grid code distribution (S1 Fig, S1.1.7 Text). Sensory noise was based on previous analyses of published rat experiments [17, 18, 40, 41], while trajectory characteristics and sensory information were constrained by rat physiology and published experimental designs (see S1.1–S1.3 Text for details).

### Grid phase noise is tolerated and necessary

**Fig 2:**
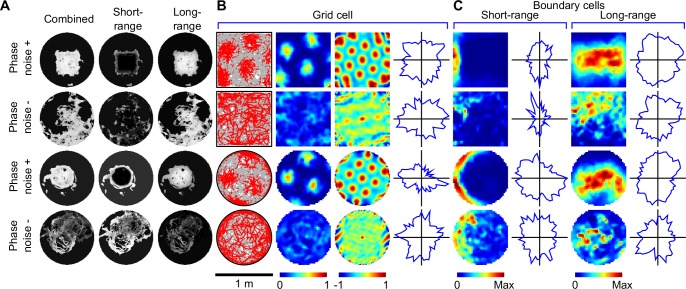
Typical learning outcomes with and without compensatory phase noise, initially naïve, with vision. (**A**) Combined, long-range and short-range association maps showed that learning arena structure requires compensatory phase noise. (**B**) Trajectories and spikes (grey lines, red dots, column 1), firing rate maps (column 2), and rate map autocorrelograms (columns 3) of probabilistic grid cells show that stable grids depend on compensatory phase noise. (**C**) Predictive boundary cells also required compensatory grid cell phase noise to show boundary-dependent responses (rate maps, columns 1 and 3). Neither probabilistic grid cells nor predictive boundary cells showed directional-selectivity (directional rate plots, (**B**) column 4, (**C**) columns 2 and 4).

In arenas similar to published grid cell experiments [10, 11, 13, 26, 34, 35], stable hexagonal grid patterns were seen (gridness index mean ± SD = 1.23 ± 0.07 in 1 m square arena, *n* = 4,000; 1.17 ± 0.11 in 1 m circular arena, *n* = 4,000) consistent with rodent grid cells [29, 42, 43] (Figs 2, S2 and S3, S1 Table). Like pure rodent grid cells [42], probabilistic grid cells were directionally-insensitive (directional information content < 0.1 bits/spike). Two distinct types of grid phase noise exist in SIFM, one arising from self-motion cues which corrupts the estimate of pose and is correlated between all grid cells, the second arising intrinsically in grid codes and is independent between grid codes but correlated across modules within each grid code. This second type of phase noise is used opportunistically to compensate for the information loss due to self-motion errors [17, 18, 30–32] (Figs 1A and S1). By virtue of its independence between grid codes, compensatory phase noise serves to sample the phase space to keep track of pose uncertainty, which is critical for successful information fusion. Without compensatory phase noise, grid cell responses were perfectly coupled, carrying redundant information, resulting in pure PI (Fig 2 rows 2 and 4) and unstable grid fields (gridness index mean ± SD = -0.05 ± 0.27 and -0.10 ± 0.29 for the square and circular arena, respectively). Notably, SIFM can also learn using spatially irregular grid codes (S4 Fig), in keeping with recent reports of anisotropic but stable grids [44].

A unique feature of SIFM are predictive boundary cells downstream of grid cells (Figs 1A and S1, S1.1 Text), which operate together with previously described sensory boundary cells [27, 38, 45]. Successful learning resulted in predictive boundary cell responses similar to rodent boundary cells [26, 27, 29] (Figs 2C, S2 and S3). However, absence of compensatory grid phase noise led to spatially-dispersed associations learned between grid and boundary cells (Fig 2A rows 2 and 4), because grid responses themselves were spatially-dispersed (Fig 2B). Hence stability of predictive boundary fields (Fig 2C) depends on grid stability (Fig 2B), so that inhibiting probabilistic learning by removing compensatory grid cell phase noise also prevents stable predictive boundary fields being established. Surprisingly, approximately one third of predictive boundary cells were unclassified using the border score [26] (b<0.5, S5 Fig) raising the possibility that many predictive boundary cells do not encode boundary information. Since some rodent boundary cells are active parallel to, but disjoint from, a boundary [26, 27], consistent with model predictions [27, 38, 45], a new hypothesis-based classification procedure was developed (S1.3.6 Text) which correctly classified predictive boundary cells, showing that boundary information is encoded (S5 Fig). Finally, some predictive boundary cells showed fractional activity along a wall (S2C and S3C Figs), similar to some rodent boundary cells [26]. Fractional activity arose from a wall being oblique to the tuning direction (bottom row of S2 and S3 Figs).

### Stable grid fields in darkness

**Fig 3:**
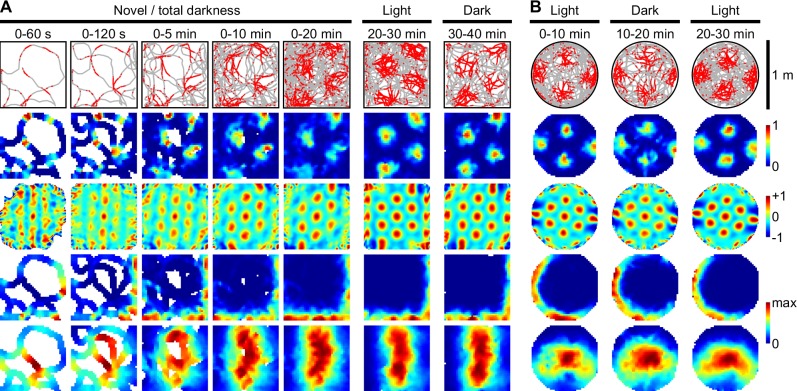
Rapid emergence and persistence of stable grid and boundary fields in darkness. (**A**) Stable probabilistic grid cell response (rows 1 to 3) in a 1 m square arena, in darkness and initially disoriented (Novel / total darkness), and during subsequent light (Light) and dark (Dark) sessions, matches rat grid cell data (Fig S7b of [10]). Predictive boundary fields (row 4 –short-range, row 5 –long-range) are also immediately stable in darkness. (**B**) Probabilistic grid and boundary fields persist in darkness in a 1 m circular arena (∞-fold rotational symmetry), matching rat grid cell data (Fig S7a of [10]).

Rodent grid cell rate maps are immediately stable in darkness in a novel room (Fig S7b of [10]), showing that grid stability and alignment do not require visual cues or familiar room landmarks. Potentially, PI is used to track location, but cumulative sensory errors cause failure within 1 to 2 minutes [17, 18], an order of magnitude less than rodent grid cell results [10]. Similarly, learning the new environment using PI would also fail (Fig 2) because PI errors accumulate if left uncorrected leading to increasing uncertainty and unstable grids [12, 46]. However, prior exposure to an identical arena in a different room [10] could plausibly have established an equivalent map in memory, which is additional information required for information fusion and grid stabilization. With prior exposure to a geometrically-equivalent arena, probabilistic grid fields are also immediately stable in darkness, despite initial disorientation (Fig 3A Novel / total darkness). Also consistent with rodent results, probabilistic grid fields remained stable in light (Fig 3A Light), and in a second darkness session (Fig 3A Dark). Re-orientation was possible due to the arena’s local rotational asymmetry, allowing the fusion of self-motion and boundary information to converge on one of four possible solutions [18]. Local arena asymmetry was shown previously to improve localization due to the limited number of rotationally equivalent poses which are consistent with sensory information [17, 18]. Hence the coupling between probabilistic grid cells and predictive boundary cells accounts for the persistence of rodent boundary fields in darkness (Fig 3A rows 4 and 5) [27].

Since circular arenas possess no local or global rotational asymmetry [18], perhaps stable grid fields in darkness (Fig S7a of [10]) cannot be explained by probabilistic models. However, SIFM slowed spatial destabilization sufficiently to allow grid fields to persist (Fig 3B). Bin-wise correlations between probabilistic grid maps in light and darkness were almost identical to rats (mean ± SEM, *n* = 33: for Light-to-Dark, *r*_SIFM_ = 0.50 ± 0.03, *r*_rat_ = 0.50 ± 0.03, *P* = 0.97, *t*_32.3_ = -0.04; for Dark-to-Light, *r*_SIFM_ = 0.53 ± 0.03, *r*_rat_ = 0.53 ± 0.03, *P* = 0.87, *t*_32.3_ = -0.17; Welch’s *t*-test), showing that persistence of grids in darkness may be largely explained by the fusion of self-motion and learned boundary information. Hence both the emergence and persistence of stable grids in darkness is explained by the same probabilistic model.

### Rescaling of grids in resized arenas

**Fig 4:**
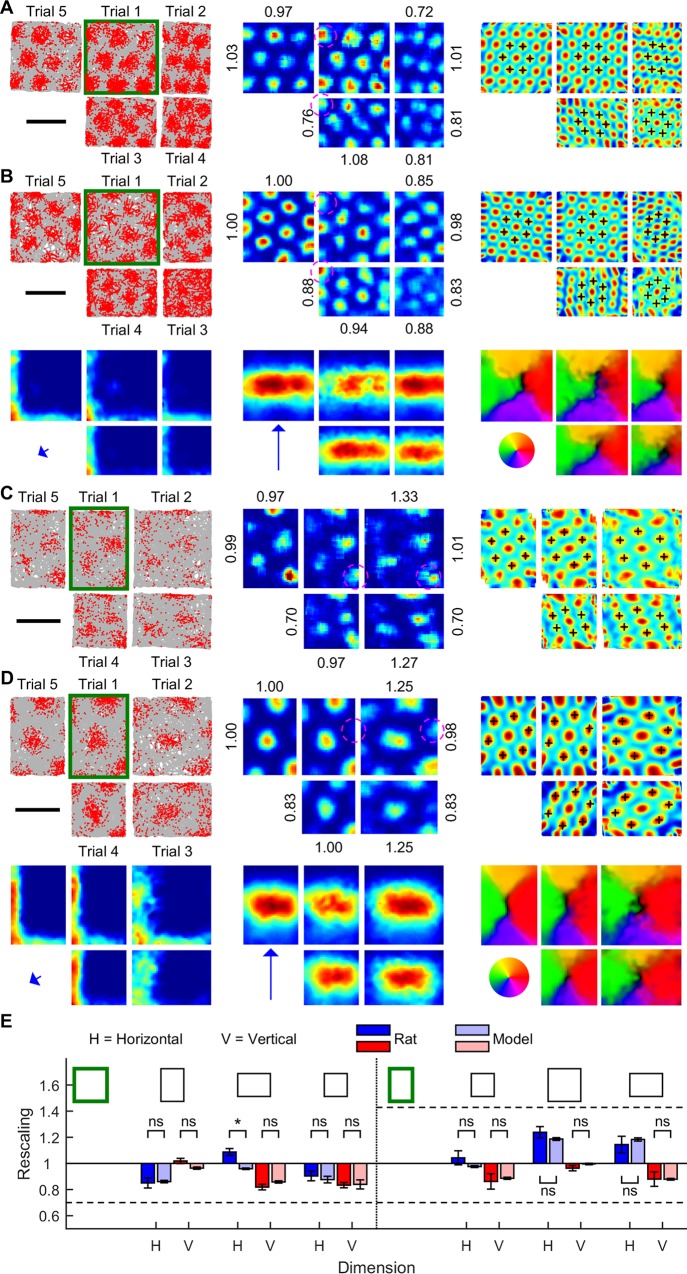
Partial rescaling of grid and boundary fields during arena resizing. (**A**) Grid cell data from a rat trained in a 1 m square arena (Trial 1), tested in three contracted arenas, and retested in the familiar arena. Rate maps (middle) and autocorrelograms (right) show partial grid rescaling principally along the contraction axes (full rescaling = 0.70, no rescaling = 1.00), causing partial loss of grid peaks (dashed circles). (**B**) Probabilistic grid cell data showing partial rescaling along arena compression axes. Typical predictive boundary cell responses (lower left–short-range, lower middle–long-range) showing stable boundary alignment across arenas, consistent with globally stable boundary vector maps (lower right). (**C**) Rat grid cell data—as per (**A**), but training in a 100 × 70 cm arena (Trial 1), and testing in both expanded and contracted arenas. Arena expansion causing partial gain of grid peaks (dashed circles; full rescaling = 0.70 (contraction) or 1.42 (expansion), no rescaling = 1.00). (**D**) Probabilistic grid cell data—as per (**B**), training in a 100 × 70 cm arena (Trial 1). (**E**) Grid rescaling magnitude (mean ± SEM; dashed line, magnitude of environmental rescaling; all *P* > 0.05 except horizontal rescaling dimension of Trial 3 in (**A**), *P* = 2.5×10^−3^, *t*_44_ = 4.6, two-sample *t*-tests, FDR corrected). (**A**-**E**) Scale bars, 50 cm. Rat data was previously published [34], with permission from C. Barry and K. Jeffery.

Grid patterns compress or stretch along the resized dimension of a test arena [34, 35], but the mechanism is poorly understood. One model assumed ideal associations between visual features and grid cells to reset grid activity [22], deforming grid patterns in resized arenas. However, grids rescaled heterogeneously over the arena (Figs 7A, B of [22]) in contrast to rodent grids [34, 35], because dominant visual features locally anchored the model grid cell’s activity. A more recent model assumed place cells, driven by boundary vector cells, reset grid cell activity [19]. Both models were heavily biased towards boundary information, thus preserving the same grid peaks despite arena resizing, differing from rodent grid cell rate maps showing partial loss or gain of grid peaks [34]. Conversely, a particle filter model underestimated grid rescaling when boundary information was used infrequently [17, 18], due to the dominance of PI. In contrast, probabilistic grids rescaled partially, nearly identical to rodent data (Fig 4). Like rodent grid cells, probabilistic grids rescaled isotropically, leading to partial loss or gain of grid peaks at boundary edges (e.g., circled grid peaks in Fig 4). Across twelve rescaling dimensions, rescaling magnitude was almost identical between probabilistic and rodent grid cells (Fig 4E). Notably, probabilistic grid rescaling in resized arenas was not based on a resizing-dependent change in the velocity signal [12], or altered oscillatory properties of grid cells. Instead, altered oscillation frequency caused omnidirectional probabilistic grid rescaling (S6 Fig) in an unchanged arena, and compatible with rodent grid expansion in novel environments [47]. In the latter, omnidirectional rescaling of probabilistic grids was caused by a change in the overall speed signal gain in the novel but geometrically equivalent arena. In contrast, the speed signal in rescaled arenas was unchanged, and grid rescaling was SIFM’s probabilistic solution to the conflict between learned and current environmental boundary information.

Although probabilistic grid patterns varied substantially with either arena geometry or oscillatory dynamics, grid stabilization depended critically on boundary information, contrary to previous argument [43]. Instead, probabilistic grid rescaling resulted from the competition between boundary and self-motion information. In resized arenas, some grid codes closely matched PI position but not boundary code, and some vice versa. High boundary prediction error suppressed the PI-matching grid codes, biasing towards grid codes which better matched the boundary input (S1 Fig, S1.1 Text), thereby gradually pulling the grid code distribution away from the PI estimate. Simultaneously, coupling of predictive boundary cells to grid cells caused predictive boundary fields to shift along the rescaling dimension (S7 Fig), distinct from sensory boundary cells whose response distance to the boundary is fixed [38, 45]. The former is a novel prediction of predictive boundary cells.

### Probabilistic learning and recall exhibits attractor-like grid cell dynamics

**Fig 5:**
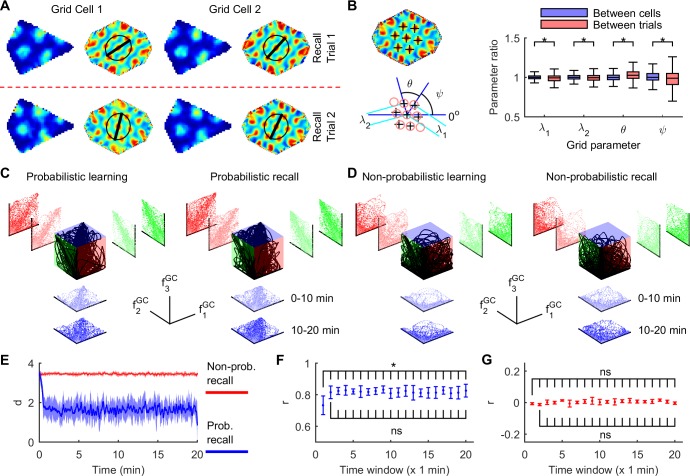
Probabilistic grid cells exhibit attractor dynamics during learning and recall. (**A**) Typical probabilistic grid cell response patterns, showing greater similarity between cells in the same trial, than in the same cell across trials (major axis shown of elliptical fit to six inner autocorrelogram peaks). (**B**) Each autocorrelogram was fitted using a regular grid template [43] (lower left, 200 grid cells × 10 independent learning trials × 2 recall localization trials). All grid parameter ratios (right) show greater variability between trials (same cell) than between cells (same trial) (*P* = {2.7×10^−22^,2.8×10^−13^,4.8×10^−16^,4.0×10^−13^}, *F*_1,1998_ = {33.9,17.4,14.2,53.6}, parameters = {*λ*_1_,*λ*_2_,*θ*,*ψ*}, Brown-Forsythe test for equal variance). (**C**) Grid activity space within a single probabilistic grid module shows clustering along the attractor (unit diagonal, perfect correlation), during naïve learning (left) and disoriented recall (right). Activity, f^GC^, of three grid cells are shown. (**D**) As per (**C**) but from non-probabilistic learning (left) and recall (right) where the boundary prediction error was always set to one. Otherwise, the distributed grid code and association maps, associative learning, and stochastic resampling were identical to SIFM, demonstrating that the attractor-like properties of SIFM require boundary prediction error feedback. (**E**) The Euclidean distance, *d* (mean ± S.D., 10 s boxcar smoothing), from the attractor (200 grid cells, grid scale module 1) from probabilistic (blue) and non-probabilistic (red) recall trials from (**C**) and (**D**). (**F**) Pearson’s correlation (*r*, mean ± SD, *n* = 10 independent recall trials) between grid cell activity (200 grid cell pairs) during Recall Trial 1 series of (**A**). Activity correlation changed significantly during re-orientation (*P* = 2.4×10^−5^, *F*_19,171_ = 3.23, one-way repeated measures ANOVA), increasing from the first minute (all *P* < 0.02, 19 paired *t*-tests, FDR corrected), then plateaued (all *P* > 0.2, 18 paired *t*-tests). (**G**) As per (**F**) but during the non-probabilistic recall series in (**D**) and (**E**). There was no change in the activity correlation during attempted re-orientation (*P* = 0.44, *F*_19,171_ = 1.03, one-way repeated measures ANOVA).

The spatial regularity and invariance of rodent grid cell responses are consistent with low-dimensional attractor properties [8, 12, 43, 48]. Local grid cell pairs showed highly correlated activity, adjusting for 2D translation, even when grid parameters were experimentally altered, suggesting strong intrinsic coupling of grid cell activity [11, 43]. For example, observed high inter-grid correlation cannot be explained simply by the stability of individual grid properties, since those properties change significantly with experimental manipulations such as arena resizing. Since information fusion in SIFM specifically requires partially uncoupled grid cells to track uncertainty in grid phase (e.g., Fig 2), perhaps probabilistic learning and recall (S1.1.8 Text) cannot explain attractor-like properties of rodent grid cells. To clearly test the strength of functional coupling between probabilistic grid cells, two recall tests were used with identical initial conditions (Fig 5A). Learning was ceased during recall tests to prevent the possibility that a dynamically changing spatial memory may contribute to session-specific correlations in grid response. Hence, if there is stronger correlation within a grid cell between trials than between grid cells in the same trial, then correlation of between-cell activity may simply reflect between-trial stability [43]. Like rodent grid cells, the opposite is true for probabilistic grid cells whose properties were more stable between cells within a trial, than in the same cell between trials (Fig 5B). The same analysis was repeated using an arena resizing series to determine if gross changes in grid parameters may weaken the functional correlation between grid cells (S8 Fig). Again, grid parameters co-varied between cells across conditions, showing a nearly identical pattern to rodent grid cells up to a scale factor. Like rodent grid cells [43], probabilistic grid cell parameter ratios also varied with omnidirectional rescaling in novel environments, with the changes virtually identical between all cell pairs within each session (S9A Fig).

Additionally, rodent grid cells show small phase changes across different test sessions in the same arena, which exceed the variability of between-cell phase offsets across the same sessions (e.g., Fig 2 of [43]). Since probabilistic grid peak locations depend on learned information, perhaps grid drift is negligible between recall sessions since learned information is unchanged. Instead, the magnitude of within-cell phase drift is significantly larger than the magnitude of change in between-cell phase offsets across two independent recall trials (S9B Fig, *P* = 3.3 × 10^−267^, *z* = 34.9, Wilcoxon rank sum test between the Between-cell and Within-cell magnitude distributions, *n* = 2,000). Similarly, the magnitude of within-cell phase drift is also larger than the phase offset of phase-matched grid cell pairs within each recall trial (*P* < 10^−300^, *z* = 42.5, Wilcoxon rank sum test between the Within-cell magnitude distribution and phase offset distribution from Recall trial 1, *n* = 2,000; *P* < 10^−300^, *z* = 44.6, Wilcoxon rank sum test between the Within-cell magnitude distribution and phase offset distribution from Recall trial 2, *n* = 2,000). These results demonstrate that probabilistic grid cells exhibit correlated phase changes across independent test sessions, similar to rodents. Notably, learned information was constant and equivalent between probabilistic recall sessions, suggesting that phase drift may primarily be a function of stochastic processes such as random movement and noise.

To determine if momentary system dynamics also display attractor character analogous to temporally-averaged grid patterns, the response of 200 phase-matched probabilistic grid cells were examined. As expected from momentary functional coupling, response time series between all grid cells within a scale module were correlated, orbiting an attractor in activity space (unit diagonal in Fig 5C). Recall localization tests were performed in darkness to exclude compass stability as a possible explanation for the high correlation between cells (using arena 1-fold rotational symmetry for global orientation [18]). In contrast, there was a clear reduction in the activity correlation between grid cells during non-probabilistic learning or recall (Fig 5D). Here, non-probabilistic learning was identical to probabilistic learning in all respects except for the loss of useful feedback from boundary prediction error thereby specifically preventing the probabilistic fusion of self-motion and boundary information while preserving the distributed estimate of pose and associative learning (S10 Fig). Initially disoriented, the perpendicular distance between the activity state and the attractor reduced and then stabilized during probabilistic recall, but remained high during non-probabilistic recall (Fig 5E). Once localized, correlation between intra-modular grid cells remained high (Fig 5F, mean *r* > 0.8 after the first minute) for probabilistic grid cells, but not for non-probabilistic grid cells (Fig 5G, mean *r* < 0.02), showing that successful localization was marked by strongly correlated grid cell activity. Taken together, these results demonstrate that probabilistic grid cells exhibit attractor properties similar to rodent grid cells, and that these properties specifically require probabilistic information fusion. Furthermore, attractor properties should be evident in darkness, initially disoriented, and on a momentary basis. SIFM also provides support that oscillatory and attractor dynamics may be complementary [19, 49]. Notably, attractor-like SIFM grid cell correlations arise from shared sensory inputs and convergence in learned information rather than direct coupling between grid cells, consistent with recent noise correlation analysis of rodent grid cells suggesting that relatively little spike train correlation is attributable to direct synaptic connections between cell pairs [50].

### Grid cell maps fragment in a multicompartment environment

**Fig 6:**
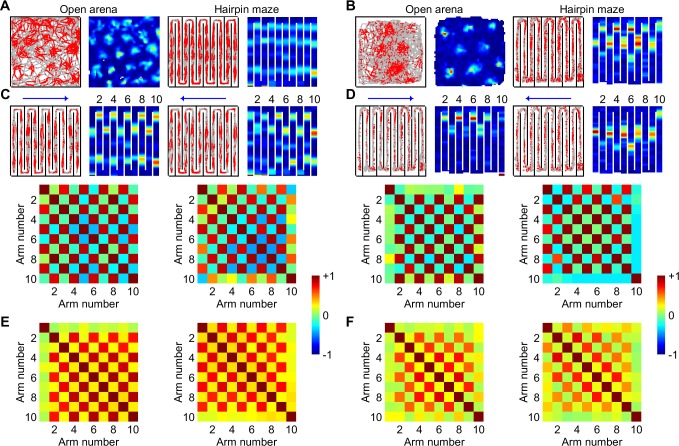
Fragmentation of grid cell maps in a multicompartment environment. Probabilistic (**A**, **C**) and rat (**B**, **D**) grid cell response patterns are both disrupted in a hairpin maze of the same dimensions as an open square arena (1.5 m × 1.5 m). Rate maps repeat across alternating arms but differed with the global running direction (easterly →, westerly ←), inconsistent with purely location-specific activity. Arm-arm rate vector correlations (10 cm bins along each arm axis) show checker-board patterns from individual probabilistic (**C**) and rat (**D**) grid cells. (**E**, **F**) Population correlation matrices showing similar alternating arm-arm rate relationships (*n* = 2,000 concatenated probabilistic grid cell rate maps (**E**), *n* = 105 from rat (**F**) using the first 20-minute session). Rat grid cell displays were constructed from data published previously [36], with permission from D. Derdikman, M.-B. Moser and E. Moser.

Hexagonal grid patterns are lost in hairpin mazes [36, 37]. Resulting grid cell rate maps fragment, repeating across alternating maze arms, acquiring a dependence on global running direction [36]. It remains unclear why inserted barriers alter grid cell activity in this particular way. It was hypothesized that grid maps fragment into multiple submaps, interconnected across hairpin turns [36, 37]. If so, probabilistic grids should not fragment since only a single map is learned (e.g., Fig 2), and no specific mechanism exists to connect or switch between maze arms. Surprisingly, probabilistic grid cell rate maps do fragment, alternate across arms in a hairpin maze (Fig 6A), and depend on global running direction (Fig 6C), similar to rats (Fig 6B and 6D). Arm-arm correlation matrices showed similar checkerboard patterns for individual grid cells (Fig 6C and 6D) and for a population (Fig 6E and 6F). Correlation between matrices from probabilistic and rat grid cells were high (easterly, *r* = 0.92, *P* = 9.9×10^−43^; westerly, *r* = 0.95, *P* = 1.8×10^−50^; element-wise correlation of population correlations of all unique arm-arm pairs), but dropped if the global running direction was mismatched (probabilistic easterly vs rat westerly, *r* = 0.82, *P* = 2.0×10^−25^; probabilistic westerly vs rat easterly, *r* = 0.86, *P* = 4.2×10^−31^), suggesting rate maps are more complex than repeating submaps across alleys.

**Fig 7:**
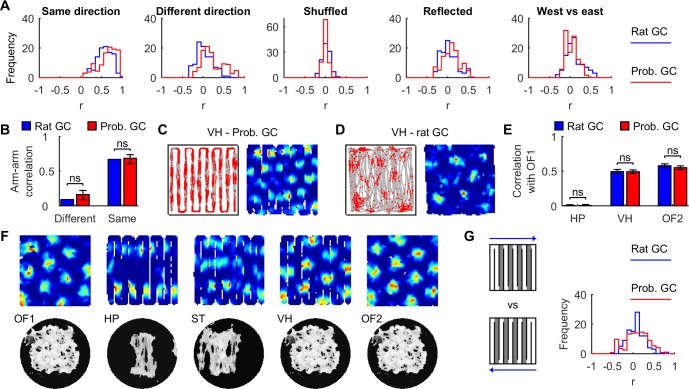
Grid fragmentation is an emergent property of probabilistic grid cells. (**A**) Distributions of mean correlations between arms where the rats ran in the: same local direction (odd-odd and even-even arms, excluding 1 and 10); different local direction (odd-even arms, excluding 1 and 10); same local direction but randomly shuffled spatial bins; different local direction but rate vectors along odd-numbered arms were reflected along north-south; and different global directions (all pairs of arms, excluding 1 and 10). (**B**) The median correlation between arms of the same or different local running direction (mean of 73 directionally-insensitive rat grid cells, compared to 95% C.I. from 10^4^ resampling means of 73 probabilistic grid cells). (**C**, **D**). Regular grid patterns along virtual hairpin (VH) trajectories in an open arena. (**E**) Spatial correlations between rate maps in the first open field (OF1) and: the hairpin maze (HP), virtual hairpin maze (VH) and a second open field (OF2), were indistinguishable between model and rat grid cells (mean ± SEM; OF1 vs HP, *P* = 0.99, *t*_199.6_ = 0.014; OF1 vs VH, *P* = 0.96, *t*_52.6_ = -0.048; OF1 vs OF2, *P* = 0.47, *t*_207.1_ = -0.73; Welch’s *t*-test). (**F**) Probabilistic grid cell rate maps (top row) and association maps (bottom row) from the hairpin maze series in a 1.5 m square arena: learning in an open field (OF1), learning in a hairpin maze (HP), learning in a semi-transparent hairpin maze (ST), recall in a familiar open field using a hairpin trajectory (virtual hairpin—VH), and recall in a familiar open field using a random trajectory (OF2). (**G**) Distributions of mean correlations between arms (shaded, excluding 1 and 10) where runs were in different global directions but the same local direction. Rat grid cell displays were constructed from data published previously [36], with permission from D. Derdikman, M.-B. Moser and E. Moser.

Like rat grid maps (Fig 7A), correlations between arms with the same local running direction were higher than for arms with: opposite local running direction (*P* = 3.5x10^-91^, *z* = 20.3, Wilcoxon rank sum test); the same local running direction but randomly shuffled bins (*P* = 4.8x10^-132^, *z* = 24.5); opposite running direction reflected along the north-south axis (*P* = 9.0x10^-112^, *z* = 22.5); opposite global running direction (east vs west, *P* = 7.5x10^-125^, *z* = 23.8). The median arm-arm correlations of directionally-insensitive rat grid cells (*n* = 73) were within the 95% resampling C.I. of probabilistic grid cells (Fig 7B; 10^4^ resamples of *n* = 73; different local direction, rat median *r* = 0.09, 95% C.I. = 0.09 to 0.22; same local direction, rat median *r* = 0.67, 95% C.I. = 0.61 to 0.74).

Perhaps grid fragmentation arose from the complex interplay between cumulative error, altered path structure in hairpin mazes, and arena geometry [15, 17, 18], not the inserted maze walls *per se*[36]. If so, grid maps should still fragment in an open arena if hairpin-like paths are followed. Instead, both probabilistic and rat grid cells (Fig 7C and 7D) preserved their hexagonal grid patterns along virtual hairpin (VH) paths [36]. It should be noted that rats were trained progressively to run to successive turn locations along an approximate virtual hairpin path in an open arena (Fig 7D), whereas SIFM grid cells were tested along an ideal hairpin path without further training (Fig 7C). Bin-wise correlations were indistinguishable between rat and probabilistic grid maps for open arena and: hairpin maze, virtual hairpin maze, and a second open arena (Fig 7E, *P* > 0.4 for all comparisons).

Contrary to the multiple submap hypothesis [36, 37], probabilistic learning resulted in a single map (Fig 7F). Multiple geometrically-similar alleys were partially compressed, reducing the east-west spatial extent while preserving the north-south extent. Incomplete compression caused easterly and westerly global runs to use non-identical map information, causing directional-dependence (Fig 6C). Compression was reduced using semi-transparent maze barriers (Fig 7F, ST), resulting in a hybrid between arm-arm repetition and global tessellation patterns. The semi-transparent maze was modelled by including perimeter boundary inputs to boundary cells at all times to provide global arena geometry information, in addition to immediately adjacent walls. Semi-transparent walls were assumed to make perimeter walls visible to the rat. Consequently, arms with similar running directions remained correlated (*P* = 1.4x10^-34^, *z* = 12.3, Wilcoxon signed rank test) but reduced compared to opaque walls (*P* = 8.4x10^-65^, *z* = -17.0, Wilcoxon rank sum test), similar to rats [36]. This shows that global boundary cues can partially disambiguate repetitive local arena structure.

To further investigate the underlying cause of map compression during probabilistic learning, two hypotheses were tested. First, perhaps the resolution of the association map was too low to reliably learn the hairpin corridor structure, leading to map compression. Probabilistic learning trials were repeated using 4-fold and 0.25-fold association map resolution, but both map compression and grid fragmentation persisted (S11A and S11B Fig, S2 Table). Second, perhaps high positional uncertainty caused boundary cues to be more heavily weighted than self-motion cues during probabilistic information fusion, leading to ambiguity between adjacent corridors. If so, a sufficient reduction in self-motion noise should recover the hairpin structure during learning, and rescue the hexagonal tessellating grid pattern, which was indeed the case (S11C and S11D Fig, S2 Table). Taken together, these results suggest that grid fragmentation and map compression are due to the interaction between cumulative self-motion error and probabilistic learning.

If a single learned spatial representation is used for both easterly and westerly runs, it may be hypothesized that re-use of the same map should be evidenced by significant similarity in the response patterns between easterly and westerly runs where the local running directions matched (Fig 7G). However, rat arm-arm correlations with the same local direction (but opposite global direction) were weakly correlated (mean ± SD = 0.07 ± 0.20, *P* = 2.4×10^−4^, *t*_104_ = 3.8, one-sample *t*-test). Surprisingly, probabilistic grid cells show similarly weak correlation (0.05 to 0.15, 95% C.I. of mean cross-correlation from 10^4^ resamples of 105 probabilistic grid cells). The low correlation in probabilistic grid cells shows that re-use of a single map is still compatible with distinct response patterns where the local running direction matched but global direction differed. Taken together, the results suggest that a single laterally compressed spatial map parsimoniously explains the fragmentation of grid maps in hairpin mazes, and is an emergent property of probabilistic learning.

### Three novel tests of probabilistic learning

**Fig 8:**
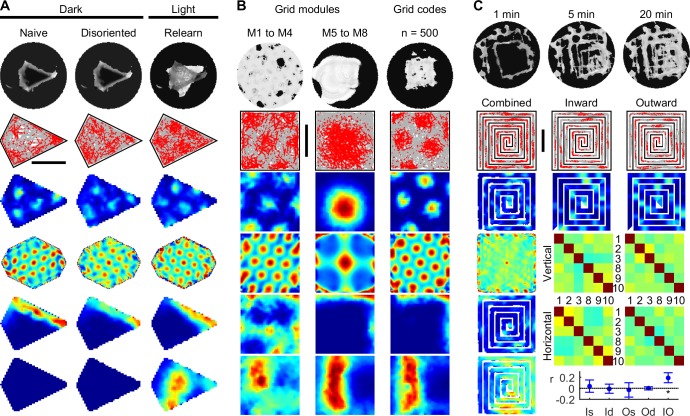
Novel predictions of probabilistic spatial learning. (**A**) Naive learning in an arena with 1-fold rotational symmetry in total darkness produced stable grid and boundary fields, persisting with reorientation in darkness, but remapping during relearning in light. Short-range boundary cell responses (row 5) follow grid cells, while long-range boundary cells (row 6) are inactive prior to light exposure. (**B**) Probabilistic spatial learning tolerates a 50% reduction of grid modules or grid codes, showing stable grids (rows 2–4), but boundary fields degrade without large grid modules (column 1, rows 5 and 6). (**C**) Learning in a spiral maze disrupts grid periodicity (column 1, rows 2–4), but maintains stable multimodal rate maps for both global inward (column 2) and outward (column 3) paths. Rate codes do not repeat across spiral arms (arm-arm correlation matrices as per Fig 6, arms numbered from left to right, bottom to top; central arms excluded due to short length). No correlation exists between rate codes along spiral arms with the same (Is, Os) or different (Id, Od) local running direction, irrespective of global inward (Is, Id) or outward (Os, Od) running direction (mean ± SD, *P* > 0.2 in each case, *n* = 10 independent trials × 400 concatenated grid rate maps, paired *t*-tests). A positive average correlation was found between rate codes along arms with opposite local and global running directions (IO, *P* = 9.1x10^-5^, *t*_9_ = 6.7). (**A** to **C**) Scale bar 50 cm.

Strong visual cues are often used to establish stable grid cell recordings [10, 11, 26, 34–36, 47]. In rat pups, stable grids emerged after eyelids unsealed and following exploratory experience [51, 52]. Even tests in darkness were performed in experienced rats [10], raising the possibility that naïve learning must fail in darkness. However, most experimental arenas have higher-order rotational symmetry in which multiple rotationally-equivalent solutions match sensory information in darkness [18]. This confound is avoided in arenas with 1-fold rotational symmetry (Fig 8A). Thus, SIFM predicts that a naïve rat can learn a spatial representation of a kite-shaped arena in total darkness, and re-localize in darkness when disoriented. Interestingly, resumed learning in light should cause global rotations of grid and boundary fields because novel long-range sensory boundary information cause large discrepancies between predictive and sensed boundary information, destabilizing the distributed estimate of pose to form a new map (Fig 8A top row). A similar mechanism may have contributed to grid remapping during alternating lights on/off training [13]. New probabilistic learning was marked by the emergence of long-range boundary cell responses in light (Fig 8A bottom row).

The stability of standard attractor network models depend critically on balanced local connection strengths [8, 12, 48, 53, 54], and it is unclear what effects local disruptions of connections may have on the spatial stability of grid cell rate maps. However, disruptions caused by the insertion of tetrodes into grid cell networks do not cause grid field drift [10, 11, 13, 34–36, 42, 47, 51, 52], suggesting that underlying computations are robust to local damage. Consistent with rodent results, SIFM tolerates abrupt loss of grid cells either through lesioning 50% of all scale modules, or 50% of grid cells spanning all modules (Fig 8B). Despite stable grids, boundary cell rate maps lost strict adherence to boundary geometry without large-scale grid modules due to increased spatial repetitiveness of the remaining grid code (Fig 8B, column 1).

Grids are predicted to fragment differently in spiral mazes compared to hairpin mazes. In a spiral maze, probabilistic grid maps are uncorrelated along the same running direction (Fig 8C, 8Is and 8Os), distinct from the hairpin maze [36, 37] (Figs 6 and 7). Instead, rate maps along arms at the same location but opposite running direction were correlated (IO), encoding place-specific information. Different patterns of grid fragmentation may therefore be signatures of probabilistically-learned spatial information, which vary with environmental structure.

Another experiment may be useful for differentiating between SIFM and standard attractor models. Distributed grid codes in SIFM are expected to decouple over time in an unbounded 2D field devoid of visual or other localizing cues. For example, a blindfolded rat which forages in a very large field (in between contacts with boundaries or other cues) can only rely on PI to keep track of location. Spatial correlations between SIFM grid cells within a module should decrease gradually due to cumulative PI errors. In contrast, grid cells in standard attractor models should remain functionally coupled and show spatially correlated drift within any one scale module.

## Discussion

Rodent grid cells show consistent and specific properties of probabilistic computations, which include grid fragmentation in hairpin mazes, attractor dynamics, partial grid rescaling in resized arenas, and stable grids in darkness. A new spatial information fusion model (SIFM) successfully performed probabilistic learning and recall using grid and boundary cells, unifying diverse grid cell response properties. This contradicts prevailing theories that grid cell networks primarily perform PI, with a separate mechanism correcting cumulative PI errors [9, 10, 12, 14, 19, 20, 22]. The latter implies that a hitherto unidentified spatial system actually solves the hard problem of SLAM. Parsimoniously, SIFM suggests that grid cells can participate directly in SLAM computations to maintain spatial stability. The remarkable similarity between SIFM and rodent data across diverse experiments also show that noisy self-motion [15–18] and boundary vector estimates [27, 38, 45] adequately encapsulate the principal sensory information used by rodent grid cells in published experiments.

SIFM provides the first demonstration that grid and boundary rate codes suffice to perform probabilistic spatial learning, despite grid codes being surjective functions of position, and boundary inputs varying substantially with the availability of vision. Additionally, probabilistic learning copes with both self-motion and boundary estimation noise, while taking advantage of intrinsic grid phase noise. The breadth and depth of similarity between probabilistic and rodent grid cell responses, partly due to emergent properties of probabilistic learning, suggest that consideration of realistic learning and perceptual constraints can lead to deeper insights into grid cell behaviour and spatial cognition more broadly.

A valid question is whether SIFM still performs PI since individual grid cells are based on a PI model which can be considered to be equivalent to a simplified oscillatory interference model (S1.1.2 Text). A key insight of SIFM is that grid stability depends crucially on maintaining a probability distribution of grid codes which is dynamically modulated through boundary prediction error. Hence PI should be treated as a special case of SIFM in which probabilistic aspects of the computations are disabled (e.g., by removing compensatory phase noise in Fig 2 or preventing prediction error feedback in S10 Fig), the latter unable to form or maintain stable grids in the presence of sensory noise. It is also important to distinguish between spatial information fusion and simpler PI-reset models. The latter can be considered a special case of information fusion where the probabilistic weight distribution is a Delta function centred on the sensory input. For example, contact with a square arena’s westerly boundary resets the grid code due to previously learned associations between a westerly boundary cell and grid cells [20]. However, such a model rigidly anchors the grid to the boundary, incompatible with partial grid expansion or contraction in resized arenas [34]. Furthermore, PI-reset relies on sensory cues directly driving the correct boundary cell’s activity. In darkness therefore, the drift in grid orientation cannot be slower than the head direction system. Yet in darkness, head direction is unstable within 2 minutes [40], whereas grid patterns remains stable for 10 to 20 minutes [10]. Finally, associative learning between boundary and grid cells is particularly challenging for any PI-reset model because of the chicken-or-egg problem of SLAM. A noisy PI estimate diverges from true position unless reset via associated boundary cells, but those associations do not exist at the start of learning. This was avoided in a recent model by assuming error-free PI [20], which is biologically unrealistic. Similarly, place cell based reset information can only be learned if sensory cues are sufficient to drive each place cell’s spatial response [19, 22]. In darkness, the same difficulties arise, where sensory cues far from a boundary are not sufficient to define position, and grid orientation should drift at least as rapidly as head direction. Hence it is specifically SIFM’s ability to perform probabilistic information fusion, rather than PI or PI-reset, which enables robust learning and recall, which in turn accounts for diverse grid cell response patterns in light and darkness. This work challenges the fundamental assumption of virtually all grid cell models that the computational problem to which grid cells provide a solution is PI.

Cues other than self-motion and boundary cues are likely to contribute to spatial stability of cell responses recorded from the hippocampal formation. Arena olfactory cues, for example, contribute to rodent place field stability [23] and may play a role in grid stability, although the latter has not been tested. However, as discussed previously [17], multiple studies showed that even careful removal of olfactory cues (including [23]) did not abolish stable place fields, including in a Morris water maze [24]. In contrast, HD cells drifted even during a single session in darkness where olfactory cues were not specifically minimized [40, 41]. Taken together, these results suggest that olfactory cues are neither necessary nor sufficient to provide a coherent explanation of stable spatial fields in darkness. A simultaneous recording of grid and HD cells in darkness in a Morris water maze may allow definitive quantification of the contribution of olfactory cues to stable grids in darkness.

Since rodent spatial fields are stable across multiple combinations of sensory cues, including without vision or olfactory cues, the underlying computations must be adaptable to variable reliability of each information stream. For example, it would be disastrous for a rat to always learn or recall by relying entirely on either stable visual or olfactory cues since they are not always there. The fact that rodent spatial cognition allows multiple information streams to contribute, and not in an obligatory all-or-none fashion, suggests some sort of probabilistic algorithm. The behaviour of grids under different manipulations and environments provide important tests of any proposed model of rodent spatial learning and recall.

In multi-compartment environments, the unexpected emergent boundary representation arising from probabilistic learning resulted in grid fragmentation (Figs 6 and 7). The learned representation is both a geometric distortion and a form of spatial information compression, in which multiple similar spatial structures are efficiently represented as one. Corridors in an opaque hairpin maze have identical geometry when considering only those walls which are visible to the rat. Thus the sensory boundary information within one corridor is equivalent to a number of other corridors. In the absence of any specific task or contextual disambiguation between the corridors, then arguably there is no need to maintain separate representations of multiple equivalent maze corridors. Potentially less neural resource is required to store a compressed representation. The close match between rodent and probabilistic grid cells in terms of global grid fragmentation, directional dependence, arm-arm-correlation matrices, a variety of arm-arm correlation distributions, and bin-wise 2D rate map similarity patterns shows that this novel and parsimonious explanation must be considered a viable alternative to the original hypothesis of multiple submaps linked at hairpin turns [36]. A working model of the latter has yet to be reported.

A large number of attractor network models have been proposed to explain grid cell responses [7–9, 12, 20, 21]. While the functional coupling between grid cells is consistent with attractor dynamics [43], the mechanism which supports such dynamics remains unclear. Here, biologically-realistic response patterns of probabilistic grid cells rely on functional coupling arising from shared self-motion inputs and feedback from boundary prediction errors, rather than static connections independent of environmental cues. Indeed, the typically rigid synaptic connectivity between attractor network grid cells would lead to near-perfect correlation in grid cell activity, voiding the ability to dynamically track growing uncertainty which is particularly important during prolonged periods in darkness. Furthermore, standard attractor networks require delicately balanced network weights to function so are highly sensitive to local damage, bringing into question their robustness to trauma, disease and even tetrode insertion. Nevertheless, a network implementation of SIFM has not been developed, and it is possible that attractor network properties may be modified to simultaneously enable: functional decoupling to track uncertainty while providing redundancy and robustness; and functional coupling via environmental inputs to maintain stability while explaining diverse grid cell responses. In that way, the shift in an attractor’s activity bump may depend on 1) self-motion cues, 2) compensatory phase noise which samples phase space, and 3) prediction error feedback such as via a boundary code. Intriguingly, cooperative oscillatory and attractor dynamics underpin SIFM function, and may also guide a connectionist instantiation of SIFM.

Overall, SIFM shows that a single probabilistic model concurrently and accurately explains numerous grid cell response properties, using realistic noisy inputs, and without assuming prior learned information. Probabilistic spatial computations manifest as a flexible yet stable set of response patterns which depend on arena information and experimental design, often indistinguishable from rodent grid cells. Hence grid cell ensembles may provide a hitherto unexplored window into probabilistic computations in a higher-order cognitive system. The dependence of grid response patterns on sensory inputs also supports the growing view that probabilistic perception complements probabilistic learning [4]. The convergence of experimental and theoretical evidence presented here suggests that spatial perception and spatial learning both depend on probabilistic interactions between grid and boundary cells.

## Methods

The new spatial information fusion model (SIFM) was developed firstly to investigate whether probabilistic fusion of realistic noisy spatial information is possible, even in principle, when constrained by using only representations which can plausibly be encoded by neuronal responses of the hippocampal formation. A second objective of SIFM was to investigate whether rodent grid cell responses are consistent with predictions using probabilistic learning and recall computations, under diverse experimental conditions. The principle of SIFM is fusion of temporally-integrated self-motion information, egocentric boundary vector information, and occasionally head direction information when available, to produce a joint estimate of the current grid code (pose) and grid-boundary associations (map) distribution (S1.1 Text). The specific implementation using noisy sensory cues, neuronal codes and a Rao-Blackwellized particle filter is briefly outlined below (see S1.1.2–S1.1.10 Text for details). This is a mathematically succinct implementation of recursive Bayesian inferencing principles, aimed at a systems-level approximation of the computations carried out by neural networks involving grid and boundary cells.

Temporally-integrated self-motion information is encoded by a population of grid cells whose responses are modulated by both speed and heading (S1.1.2 Text). To function, SIFM requires a temporally-stable function of spatial phase, which need not be a regular grid (e.g., S4C Fig). Noisy self-motion cues provide approximate linear and angular displacement inputs to grid cells, which in turn have independent phase noise which plays a compensatory role (S1.1.3 Text). Noisy boundary cues provide short-range vectorial information to boundary cells when within somatosensory contact range, and long-range vectorial information when vision is available (S1.1.4 Text). A noisy compass cue is provided in the presence of vision (S1.1.5 Text). Grid-boundary associations are approximated by a linear average over time, over a set of predefined map grid codes (S1.1.6 Text). Each active grid code (activity of phase-correlated grid cells in multiple modules) and its association map corresponds to a single ‘particle’ in the particle filter implementation. Using learned grid-boundary associations, new grid codes generate predictive boundary codes, whose discrepancy with sensory boundary information yields a prediction error and importance weight for particle resampling (S1.1.7 Text). Note that the population of predictive boundary cells have distinct properties from sensory boundary cells which have been described previously [27, 38, 45]. Only predictive boundary cell responses are presented. Disorientation is modelled as a random redistribution of grid code activity (S1.1.8 Text). Pseudocode summarizes key implementation steps for probabilistic learning (S1.1.9 Text) and recall (S1.1.10 Text).

Random simulated trajectories were used to provide full coverage of each arena, mimicking the behaviour of trained rats, except in hairpin mazes where rats were trained to run along maze corridors (S1.2 Text).

Methods for calculating firing rate maps, spatial crosscorrelograms, gridness index, grid rescaling and border score have been described previously so are only briefly summarized (S1.3.1–S1.3.5 Text). The parametric rate map correlation was developed to determine whether a cell’s response is more grid-like or boundary-like, based on computational hypotheses of each cell type (S1.3.6 Text). Unlike the border score, this metric correctly classified long-range model boundary cells. Associative weight maps were displayed by averaging across all boundary codes at the nominal position corresponding to each grid code of an association map (S1.3.7 Text). For visual comparison with published data, grid cell and boundary cell spikes were simulated using an inhomogeneous Poisson process (S1.3.8 Text). Predictive short-range boundary vector maps were produced to visualize the learned local boundary direction (S1.3.9 Text). Spike-triggered dynamic rate maps and autocorrelograms were used to detect underlying spatial regularity in response patterns which may drift over time (S1.3.10 Text). Grid phase change was quantified both between grid cells and within the same grid cell across different recall trials in darkness in a kite arena (S1.3.11 Text).

## Supporting Information

S1 Fig Using boundary prediction error in a probabilistic learning model. Three example grid codes and their associative weights with predictive boundary codes are shown (numbered [i-1], [i] and [i+1]). Environmental cues provide egocentric boundary vector information in the animal’s egocentric reference frame, and transformed via each grid code’s private heading estimate to an allocentric boundary code (sensory). Self-motion cues (angular and linear displacement estimates) cause grid cell phase shift, in conjunction with compensatory phase noise. Via each associative weight matrix, a boundary code (predictive) is generated from its corresponding grid code, compared to the sensory boundary code, producing an error signal (Error). The magnitude of each error determines the probability of a grid code and its association weight matrix being replaced during resampling (indicated by the red feedback arrow). Concurrently, associative weights between grid and predictive boundary codes continually update using current sensory boundary information. See also S1.1 Text.
(TIF)

S2 Fig Examples of probabilistic grid and predictive boundary cell responses from a single learning trial. (**A**) Trajectory (grey lines) and spikes (red dots) are shown for one representative grid cell from 8 grid scale modules during a single learning trial of 20 minutes with vision in a 1 m square arena. Rate maps (row 2) and autocorrelograms (row 3) show spatial periodicity, up to arena size. (**B**) Rate maps of short-range predictive boundary cells, showing activity along either one or two adjacent arena walls. The radial tuning function of each row of boundary cells is shown in cyan (left column, the maximum boundary contact range is indicated by a red line). (**C**) In addition to the properties of short-range boundary cells, some rate maps of long-range boundary cells were disjoint from boundaries parallel to the field, similar to both a subset of subicular boundary vector cells [27], and also a subset of medial entorhinal neurons [26] which do not fit the current definition of border cells. Also similar to a subpopulation of medial entorhinal border cells, some predictive boundary fields were restricted along a wall (arising from a response to more distant boundaries rather than the adjacent walls). The ideal tuning direction for each boundary rate maps is shown (bottom row, 95% C.I. shaded).
(TIF)

S3 Fig Effects of a single barrier on probabilistic grid and boundary cell responses. As per S2 Fig but with a 50 cm barrier inserted (vertical white line). Predictive boundary cell activity was seen along both the perimeter boundary and along the interior barrier, consistent with rodent boundary vector cells and border cells in subiculum and medial entorhinal cortex [26, 27].
(TIF)

S4 FigGrid and map regularity are not required for probabilistic spatial learning.(**A**) Example of an association map and magnified subregions (□ and □) learned using a regular hexagonal array of association map grid codes. Typical grid cells (columns 2 and 3) and predictive boundary cells (columns 4 and 5), showing tessellating and boundary-following responses, respectively. Estimated boundary cell tuning functions (row 3, columns 4 and 5) show response concentrated at a single allocentric boundary direction and distance. Estimated optimal boundary cell tuning (+) was within 1 SD of ideal tuning parameters (ellipse). (**B**) As per (**A**) but learning with irregular association map spatial codes (left column), showing similar grid cell and boundary cell properties (uniformly random spatial locations at the same mean density as **A**). (**C**) As per (**A**) but learning with irregular association map spatial codes (left column) and irregular grids (columns 2 and 3), showing that predictive boundary fields also tolerate irregularity in the grid codes used for probabilistic learning (uniformly random spatial locations at the same mean density as **A**, and randomly chosen grid cell oscillatory components: *ϕ*_*dj*_ ∼ *N*(*jπ*/3,0.2^2^) in radians). Despite loss of regular grid patterns, grid cell responses remained spatially selective and temporally stable.(TIF)

S5 FigParametric rate map correlation.(**A**) The rate map and autocorrelogram (top row) of a probabilistic grid cell in a 1 m circular arena showing a low border score (b < 0.5) and high gridness index (g > 0), consistent with the current definition of a grid cell. Using a boundary vector cell hypothesis (row 2), and a simplified oscillatory interference grid cell hypothesis (row 3), a parameter map (left) and reconstructed rate map (right) are shown (see S1.3.6 Text for details). Since the reconstructed rate map using a grid cell hypothesis better matched the original rate map (higher *r*) than using a boundary cell hypothesis, this was classified correctly as a grid cell. (**B**) As per (**A**) but data was from a short-range predictive boundary cell, showing that border score, gridness index and parametric rate map correlation coefficients are in agreement that it is a boundary cell. (**c**) Gridness index[43] vs border score [26] of grid cells (●, *n* = 8,000) and boundary cells (●, *n* = 2,640) from 20 recall trials in a 1 m circular arena (including data from (**A**) and (**B**)), showing standard threshold values (cyan lines). Probabilistic grid cells (GC) were classified with high sensitivity (sens.) and specificity (spec.), but 31% of predictive boundary cells (BC) were unable to be classified (uncl.). Note that some cells could not be plotted because at least one metric was undefined. Only those boundary cells tuned between 3 and 100 cm were included for analysis, due to arena size constraint and analysis spatial sampling resolution. (**D**) For the same data as (**C**), parametric rate map correlations are shown under a boundary vector cell hypothesis, r(Hyp:BVC), and a simplified oscillatory interference grid cell hypothesis, r(Hyp:GC). Unclassified cells (uncl.) were defined as those where both correlation coefficients were below 0.5. (**E**) As per (**A**) but in a 1 m square arena with irregular grid axes and grid scales. Normally, this would not be classified as a grid cell (low gridness). In contrast, use of parametric rate map correlation coefficients correctly classifies this as a grid cell. (**F**) As per (**C**) but data was from a long-range boundary cell. Normally, this would not be classified as a boundary cell (low border score). In contrast, use of parametric rate map correlation coefficients lead to the correct classification. (NaN = not a number, arising from insufficient peaks being found in the autocorrelogram to calculate a gridness index.) (**G**) As per (**C**) but using data from 10 independent learning trials in a 1 m square arena with noisy grid axes and grid scales (including data of (**E**) and (**f**); 4,000 grid cells, 1,320 boundary cells), showing over a third of both grid and boundary cells as unclassified. (**H**) As per (**D**) but using the data from the 1 m square arena of (**E**) and (**F**). (**I**) As per (**C**) and (**G**), but pooled over all SIFM data sets in open 2D environments with vision (72,000 grid cells, 23,760 boundary cells), showing 38% of boundary cells as unclassified based on the border score and gridness index. The marker size was reduced for clarity. (**J**) As per (**I**), but using parametric rate map correlation coefficients to achieve high classification sensitivity (97–99%) and specificity (97–99%) for both grid and boundary cells.(TIF)

S6 FigGrid expansion in novel environments is consistent with reduced self-motion gain.(**A**) The effect of environmental novelty was modelled as reduced self-motion gain in a 1 m square arena (Novel 1, gain = 6/9; Novel 2, gain = 7/9; Novel 3, gain = 8/9; Familiar 2, gain = 1), following learning (Familiar 1, gain = 1). Grid fields were evident (Novel 1 to 3), showing graded grid expansion (rows 1 to 3). Boundary cell activity also persisted despite conflict with self-motion cues, showing that identical sensory cues and learned information can be used to stabilize multiple distinct grid patterns resulting from reduced self-motion gain. (**B**) Ideal temporal dynamics of probabilistic grid cells in familiar and novel environments, from grid scale module 1 and 2 (M1 and M2, respectively), with somatic input based on the speed-independent theta frequency reduction in rats exposed to novel environments [47, 55]. (**C**) Somatic (+) and dendritic (◊, ○) oscillatory frequencies of grid scale module 1 (○) and 2 (◊), corresponding to the reduced self-motion gains of (**A**). (**D**) Predicted (◊, ○) and actual (◊, ○) grid spacing (mean ± SD) of probabilistic grid cells diverge in novel environments, showing that oscillatory interference parameters do not fully determine grid scale if probabilistic computations are used.(TIF)

S7 FigArena resizing leads to partial rescaling of predictive boundary field distances to boundaries.(**A** and **B**) Two examples (rows 1 and 2) of predictive boundary cell rate maps from two separate arena resizing series. From boundary cells with tuning directions perpendicular to the boundaries, tuning distances were estimated along the ideal tuning direction (arrow). Field position (─) was estimated by the rate map’s center of mass, thresholded by its mean, i.e., $\Theta \left(f-\bar{f}\right)$. (**C** and **D**) All estimated field distances are shown against their ideal tuning distance. The red line is the equivalence line, adjusted for the wrapped Gaussian angular tuning distribution which reduces the mean perpendicular boundary detection distance, i.e., *y* = *xExp*(−*σ*_*θ*_^2^/2) where *σ*_*θ*_ = *π*/12 in this model. Arena compression consistently reduced boundary field distances (below the red line) while arena expansion increased field distances (above the red line). (**E**) Predictive boundary cells with optimal tuning distances of 16.2 and 33.8 cm from the boundary were used to determine how predictive boundary field positions relate to the rescaling magnitude of grid cells. Other tuning distances were excluded because they were either too long for their fields to be within the arena, or too short for their narrow fields to be adequately sampled by the random trajectory and 2 cm spatial bins. Absolute field position along each tuning direction was scaled to both Trial 1 and 5, the average of which was treated as one vertical (collapsing north and south together) or horizontal (collapsing east and west together) rescaling estimate. The magnitude of partial rescaling was statistically indistinguishable from rat grid cells along all twelve dimensions tested (*P* > 0.05, two-sample *t*-tests, FDR corrected), demonstrating that predictive boundary field rescaling matches rat grid rescaling during arena resizing trials.(TIF)

S8 Fig Grid parameters remain correlated despite gross changes through arena resizing. (**A**) Rate map autocorrelograms for grid cells in a familiar environment (trials 1 and 5) and resized versions of the familiar environment (trials 2–4). (**B**) The 7 central autocorrelogram peaks were used to find the 4 grid parameters which defined an ideal tessellating grid. The grid axis orientations were chosen to match the convention of Fig 3 of [43]. (**C**) Grid parameter ratios (mean ± SD) are shown for probabilistic (left) and rat (right) grid cells, comparing parameter rescaling relative to trial 1 (top two rows) in distinct cell pairs within each trial (nominally designated Cell 1 and Cell 2; *n* = 1,000 probabilistic grid cells per group, *n* = 100 bootstrap samples from rat data—[43] Online Methods). The similarity between probabilistic grid cell subpopulations reflects the large sample size used. The pattern of relative rescaling of grid parameters were nearly identical between rat and probabilistic grid cells (mean rescaling ratios—rat cell 1 vs model cell 1: *r* = 0.93, *P* = 2.6 × 10^−7^; rat cell 2 vs model cell 2: *r* = 0.98, *P* = 8.8 × 10^−12^). In both, the unitary ratio of grid parameters between cell pairs within each trial (bottom row: left–model, right—rat) showed grid parameter stability between cells within a trial, despite substantial variability across trials. Note: SIFM grid parameters underestimate the magnitude of rescaling because grid orientations are random, whereas recent analyses suggest that rat grids align closely with one rectangular boundary [11, 44].
(TIF)

S9 FigProbabilistic grid drift and omnidirectional rescaling show attractor-like properties.(**A**) Using template-fitted grid parameters, normalized grid phase differences are compared between cell pairs within a trial (Recall trial 1, Recall trial 2, Between-cell), and within a cell across independent recall trials (Within-cell) in a kite arena in darkness. Phase offsets between cell pairs within each trial ($\delta_{1}^{\alpha \beta }$, $\delta_{2}^{\alpha \beta }$) can be compared directly because SIFM grid cells are phase-matched. The difference in the phase offset of the corresponding cell pairs across the two recall sessions, i.e., $\Delta_{t}\left(\delta_{1}^{\alpha \beta }\right)$ and $\Delta_{t}\left(\delta_{2}^{\alpha \beta }\right)$, showed less variability than within-cell phase changes along the same grid template axes, i.e., $\Delta_{t}\left(\phi_{1}^{\alpha }\right)$ and $\Delta_{t}\left(\phi_{2}^{\alpha }\right)$. Below each normalised phase plot, the corresponding probability mass function of the radial magnitude of drift is shown (black line), superimposed on all four probability distributions (grey lines). (**B**) Using results from S6 Fig which modelled environmental novelty by reducing speed gain, probabilistic grid parameters were fitted as shown on the right (see also S8 Fig and [43]). Relative to the familiar environment (Familiar 1, gain = 1), rescaling of grid parameter ratios (mean ± SD) from distinct cell pairs within each trial (nominally designated Cell 1 and Cell 2; *n* = 1,000 probabilistic grid cells per group) varied within cells over multiple novel sessions (Novel 1, 2 and 3) despite being in a geometrically-identical arena (1 m square). In contrast, the grid parameter ratios between cells were unchanged (lower panel, close to unity).(TIF)

S10 Fig Boundary prediction error feedback is critical for probabilistic learning. Typical examples of learning (**A**, **C**) and recall (**B**, **D**) are shown either with (**A**, **B**–probabilistic) or without (**C**, **D**–non-probabilistic) boundary prediction error feedback. All other model parameters were identical, including distributed grid codes with compensatory phase noise, associative learning between grid and boundary codes, and in darkness. During non-probabilistic learning and recall (**C**, **D**), boundary prediction error was set constant to negate its influence on the distribution of grid codes and association maps, impairing probabilistic information fusion and preventing the arena geometry from being learned (**C** row 1, compared to **A** row 1). Grids were only evident in standard rate maps and autocorrelograms (static) from probabilistic learning and recall, while spike-triggered dynamic rate maps and autocorrelograms (dynamic [56]) showed some grid-like spatial patterns even during non-probabilistic learning and recall, reflecting the underlying iPI process. Similarly, short-range predictive boundary cells showed inconsistent and dispersed responses without boundary prediction error feedback, leading to loss of oriented structure in boundary vector maps (**C**, **D**—lower right, compared to **A**, **B**–lower right). Long-range boundary cells were inactive due to lack of vision.
(TIF)

S11 Fig Grid fragmentation is affected by self-motion noise magnitude but not association map resolution. Grid fragmentation in a 1.5 m hairpin maze persisted using association maps with 4-fold (**A**, halving *σ*_*g*_ and *d*_max_, adjacent grid codes separated by 1 cm) and 0.25-fold (**B**, doubling *σ*_*g*_ and *d*_max_, adjacent grid codes separated by 4 cm) spatial resolution (S1.1.6 Text). The fragmentation pattern of individual grid cells depended on global running direction, forming distinct checkerboard arm-arm correlation matrices. Grid fragmentation also persisted when self-motion linear and angular noise variances were increased 4-fold (**C**), but was largely abolished when self-motion linear and angular noise variances were decreased to 0.25-fold (**D**). Under normal to high self-motion noise, the learned spatial layout of the hairpin maze was laterally compressed (**A** to **C**, column 1), irrespective of the underlying resolution of the association map (magnified inset), preventing the hexagonal tessellating grid pattern from emerging (columns 6 and 7). Under low self-motion noise, the hairpin maze structure was evident following learning (**D**, column 1), as were hexagonal tessellating grids (columns 6 and 7). See S2 Table for correlations between arm-arm correlation matrices of probabilistic and rat grid cells.
(TIF)

S12 Fig Typical learning outcomes with alternate associative learning rule based on boundary prediction error. (**A**) Combined, long-range and short-range association maps following probabilistic learning using a prediction error feedback rule and different learning rates *η*, initially naïve and with vision. (**B**) Trajectories and spikes (grey lines, red dots, column 1), firing rate maps (column 2), and rate map autocorrelograms (columns 3) of probabilistic grid cells showing stable grids across an order of magnitude in learning rates. (**C**) Predictive boundary cells also showed boundary-dependent responses across the same range of learning rates (rate maps, columns 1 and 3). Neither probabilistic grid cells nor predictive boundary cells showed directional-selectivity (directional rate plots, (**B**) column 4, (**C**) columns 2 and 4).
(TIF)

S1 Text The supporting text provides further details on the spatial information fusion model (S1.1 Text), trajectory models (S1.2 Text) and data analysis (S1.3 Text). (PDF)

S1 Table Stable grids require compensatory phase noise. Gridness index (mean ± SD) of probabilistic grid cells following learning with (+) and without (-) compensatory phase noise in 1 m arenas. See S1.3.3 Text for definitions of the four variants of the gridness index used.
(DOC)

S2 Table Grid fragmentation caused by self-motion noise. Correlation between population arm-arm correlation matrices from probabilistic and rat grid cells in a 1.5 m hairpin maze (excludes main diagonal of arm-arm correlation matrices).
(DOC)

S1 Code SIFMMatlabCode contains scripts and functions to run learning (SIFM_ProbabilisticLearning.m) and recall (SIFM_ProbabilisticRecall.m) experiments using SIFM in rectangular arenas, written in Matlab R2015a. Following a learning or recall trials, probabilistic grid cell responses, predictive boundary cell responses, and association maps can be displayed using SIFM_DisplayCellResponse.m.
(ZIP)
