# Supplementary figures and images for: Probabilistic Learning by Rodent Grid Cells
Source: PLoS Comput Biol. 2016 Oct 28;12(10):e1005165. doi: 10.1371/journal.pcbi.1005165 (PMC5085080; doi:10.1371/journal.pcbi.1005165)

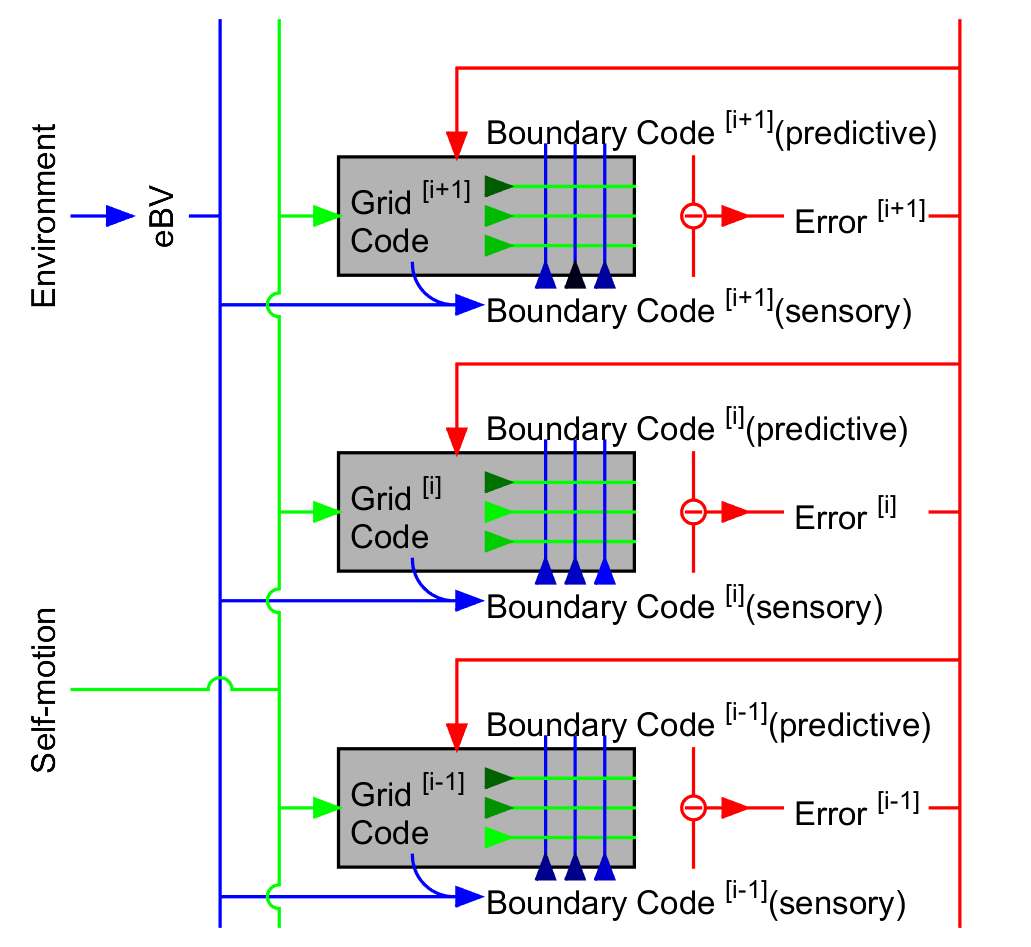

Supplement: S1 Fig — Three example grid codes and their associative weights with predictive boundary codes are shown (numbered [i-1], [i] and [i+1]). Environmental cues provide egocentric boundary vector information in the animal’s egocentric reference frame, and transformed via each grid code’s private heading estimate to an allocentric boundary code (sensory). Self-motion cues (angular and linear displacement estimates) cause grid cell phase shift, in conjunction with compensatory phase noise. Via each associative weight matrix, a boundary code (predictive) is generated from its corresponding grid code, compared to the sensory boundary code, producing an error signal (Error). The magnitude of each error determines the probability of a grid code and its association weight matrix being replaced during resampling (indicated by the red feedback arrow). Concurrently, associative weights between grid and predictive boundary codes continually update using current sensory boundary information. See also S1.1 Text. (TIF) [file pcbi.1005165.s001.tif]

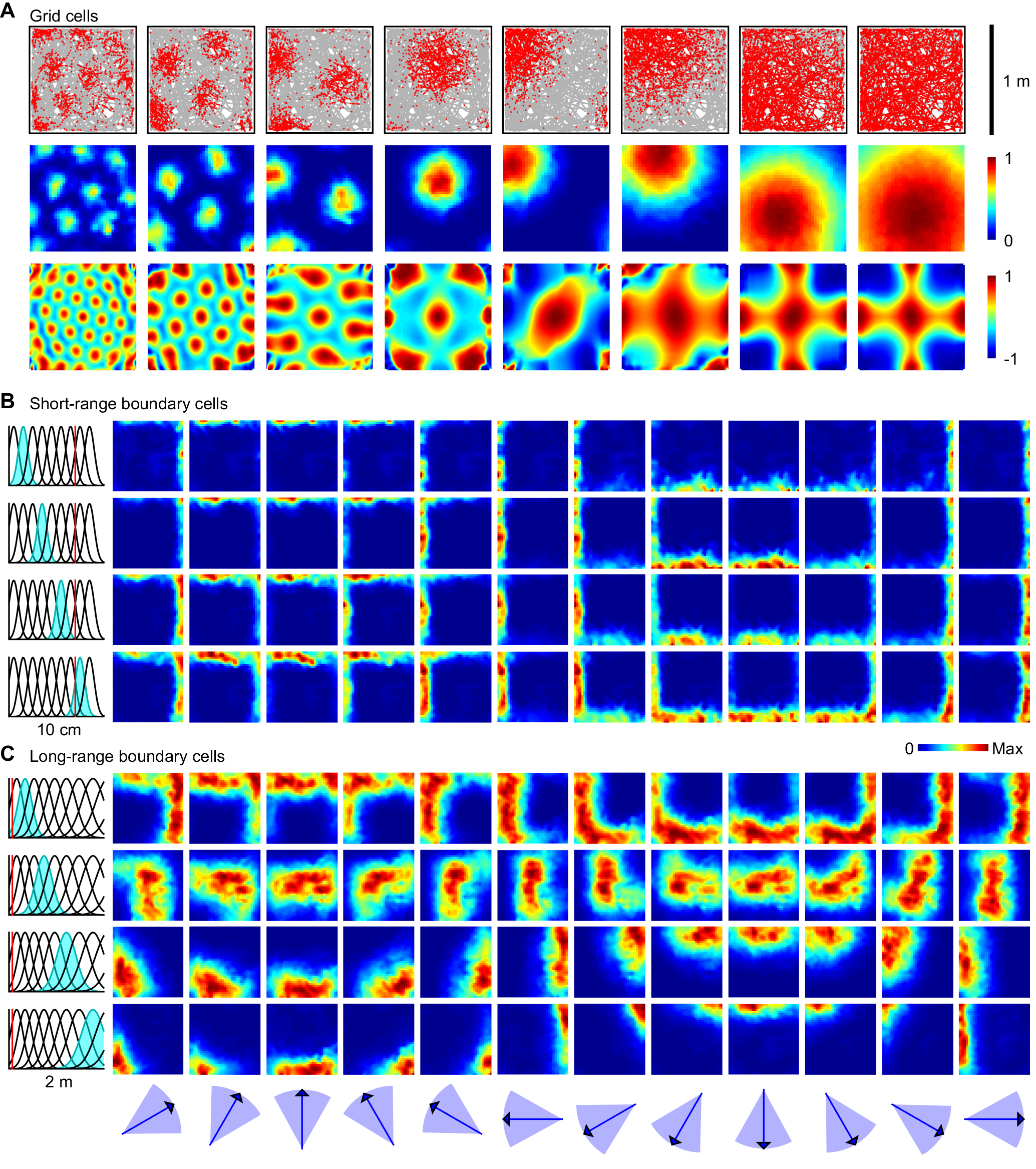

Supplement: S2 Fig — (A) Trajectory (grey lines) and spikes (red dots) are shown for one representative grid cell from 8 grid scale modules during a single learning trial of 20 minutes with vision in a 1 m square arena. Rate maps (row 2) and autocorrelograms (row 3) show spatial periodicity, up to arena size. (B) Rate maps of short-range predictive boundary cells, showing activity along either one or two adjacent arena walls. The radial tuning function of each row of boundary cells is shown in cyan (left column, the maximum boundary contact range is indicated by a red line). (C) In addition to the properties of short-range boundary cells, some rate maps of long-range boundary cells were disjoint from boundaries parallel to the field, similar to both a subset of subicular boundary vector cells [27], and also a subset of medial entorhinal neurons [26] which do not fit the current definition of border cells. Also similar to a subpopulation of medial entorhinal border cells, some predictive boundary fields were restricted along a wall (arising from a response to more distant boundaries rather than the adjacent walls). The ideal tuning direction for each boundary rate maps is shown (bottom row, 95% C.I. shaded). (TIF) [file pcbi.1005165.s002.tif]

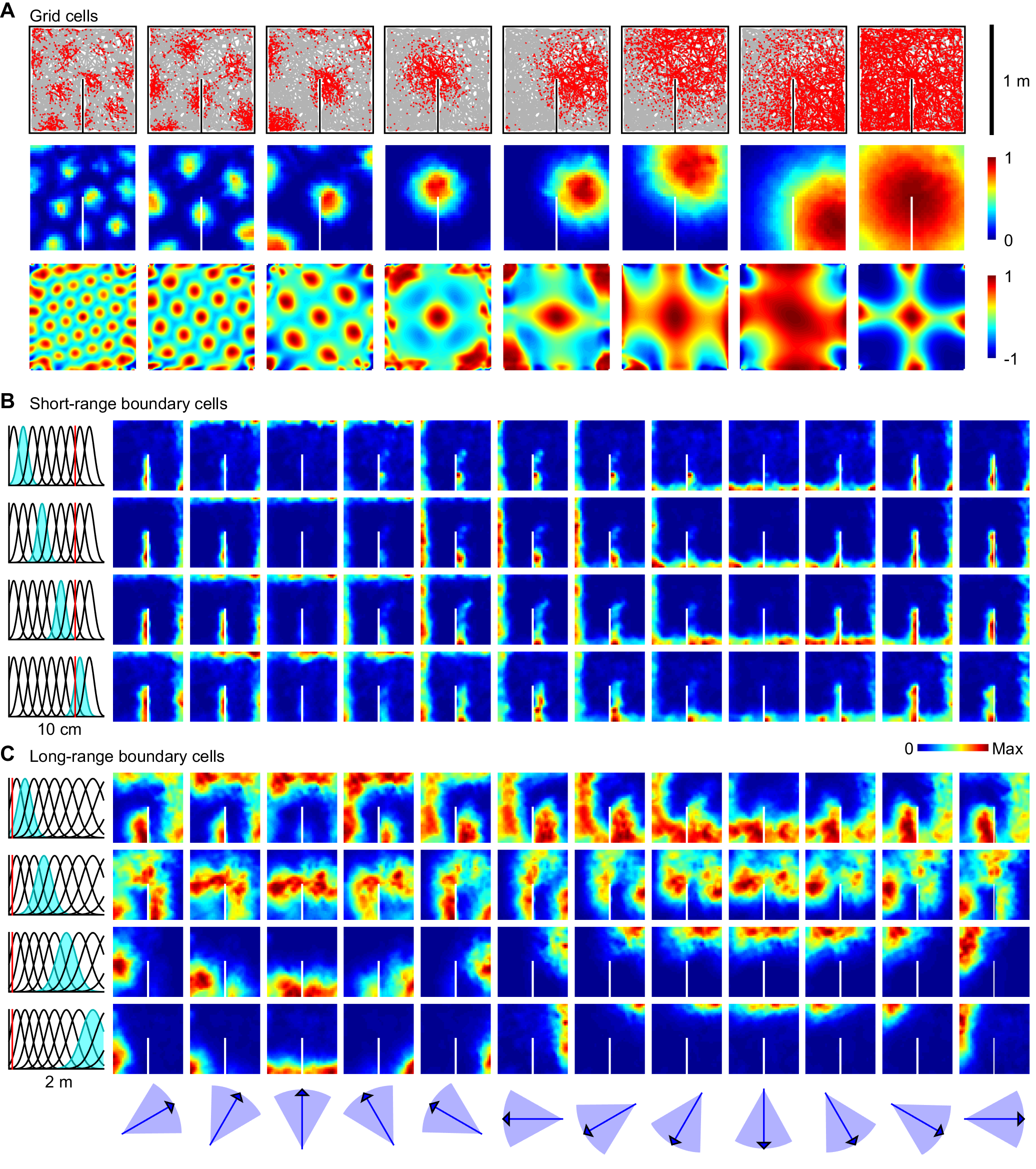

Supplement: S3 Fig — As per S2 Fig but with a 50 cm barrier inserted (vertical white line). Predictive boundary cell activity was seen along both the perimeter boundary and along the interior barrier, consistent with rodent boundary vector cells and border cells in subiculum and medial entorhinal cortex [26, 27]. (TIF) [file pcbi.1005165.s003.tif]

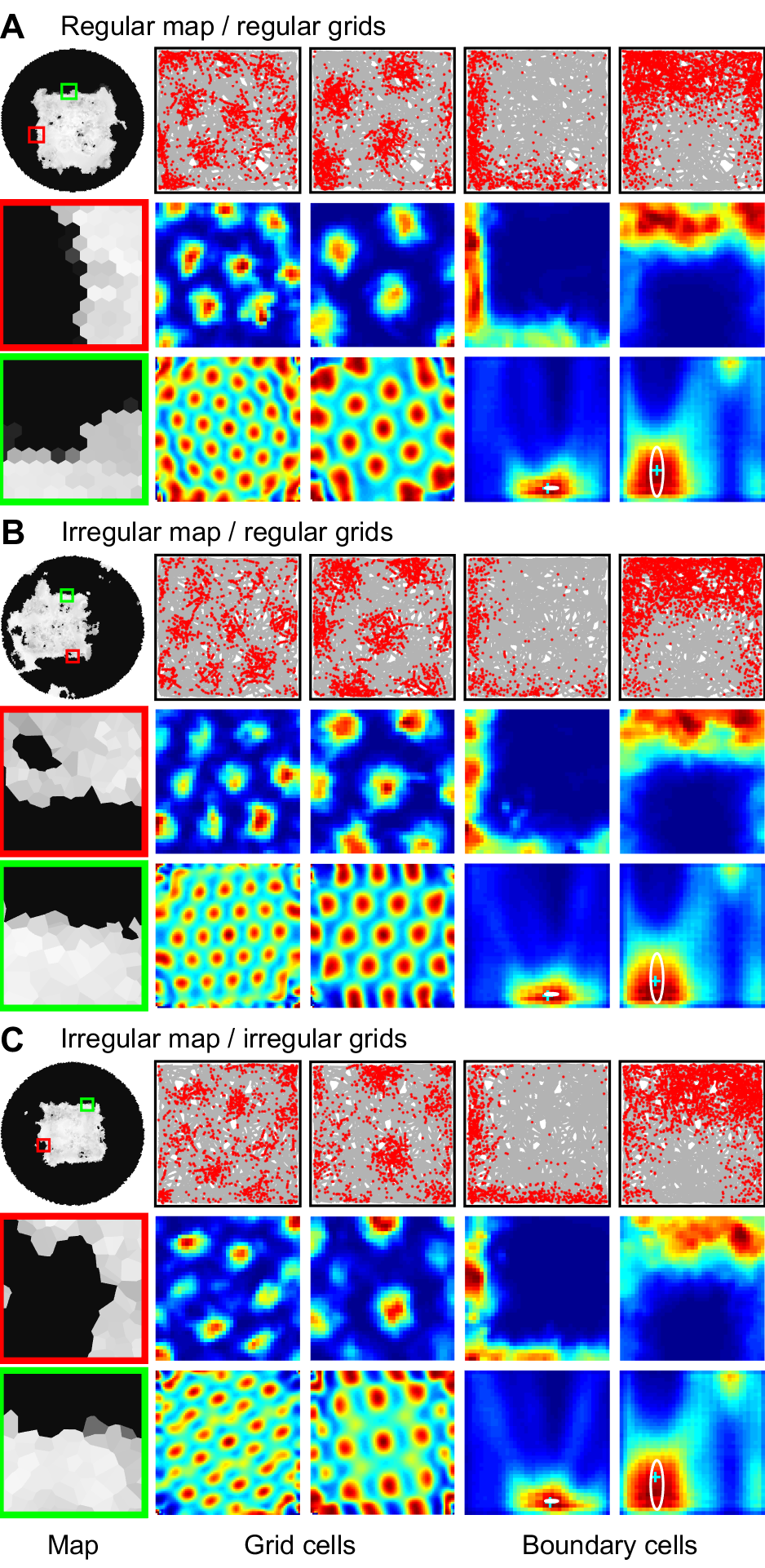

Supplement: S4 Fig — (A) Example of an association map and magnified subregions (□ and □) learned using a regular hexagonal array of association map grid codes. Typical grid cells (columns 2 and 3) and predictive boundary cells (columns 4 and 5), showing tessellating and boundary-following responses, respectively. Estimated boundary cell tuning functions (row 3, columns 4 and 5) show response concentrated at a single allocentric boundary direction and distance. Estimated optimal boundary cell tuning (+) was within 1 SD of ideal tuning parameters (ellipse). (B) As per (A) but learning with irregular association map spatial codes (left column), showing similar grid cell and boundary cell properties (uniformly random spatial locations at the same mean density as A). (C) As per (A) but learning with irregular association map spatial codes (left column) and irregular grids (columns 2 and 3), showing that predictive boundary fields also tolerate irregularity in the grid codes used for probabilistic learning (uniformly random spatial locations at the same mean density as A, and randomly chosen grid cell oscillatory components: ϕdj ∼ N(jπ/3,0.22) in radians). Despite loss of regular grid patterns, grid cell responses remained spatially selective and temporally stable. (TIF) [file pcbi.1005165.s004.tif]

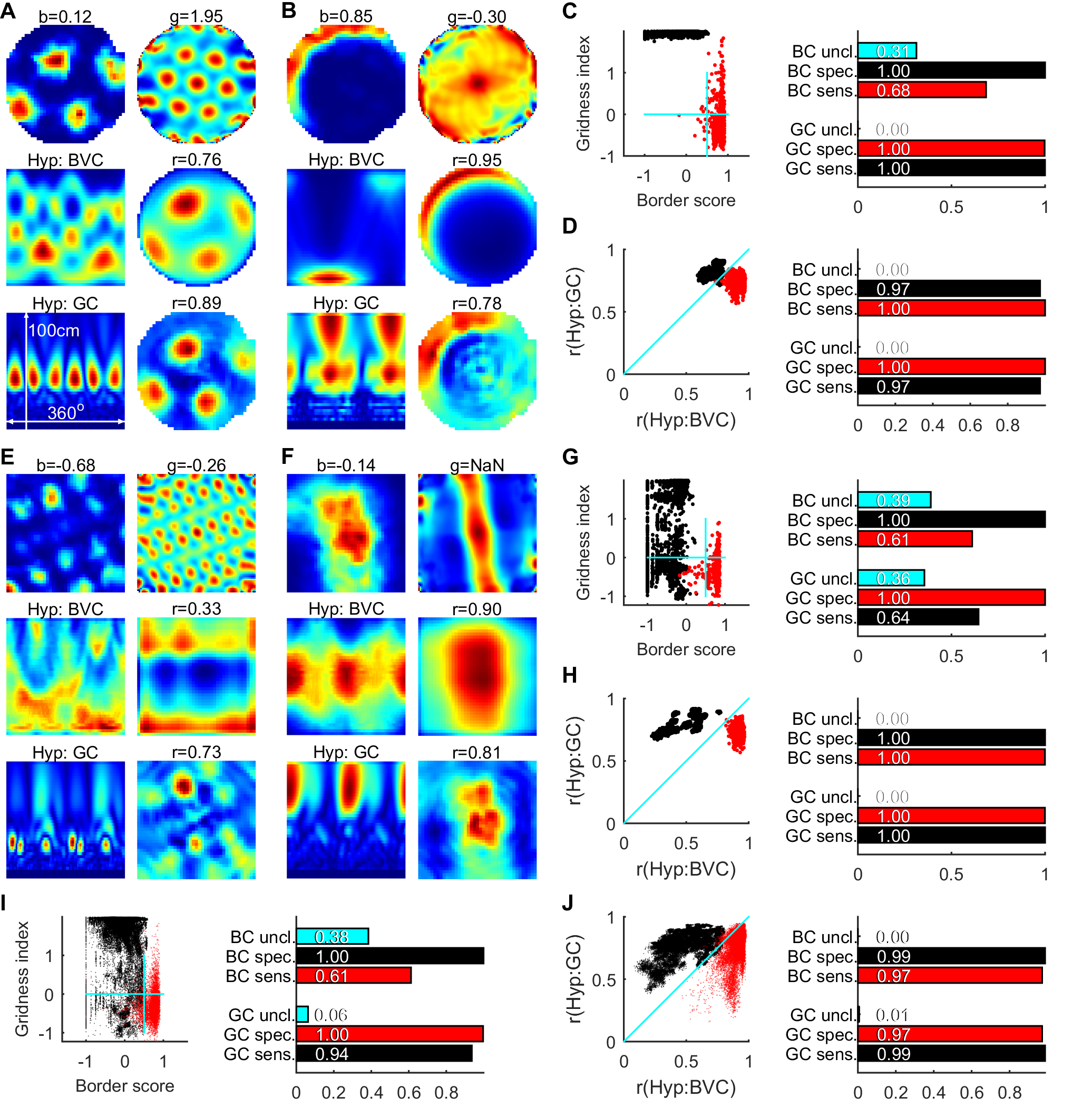

Supplement: S5 Fig — (A) The rate map and autocorrelogram (top row) of a probabilistic grid cell in a 1 m circular arena showing a low border score (b < 0.5) and high gridness index (g > 0), consistent with the current definition of a grid cell. Using a boundary vector cell hypothesis (row 2), and a simplified oscillatory interference grid cell hypothesis (row 3), a parameter map (left) and reconstructed rate map (right) are shown (see S1.3.6 Text for details). Since the reconstructed rate map using a grid cell hypothesis better matched the original rate map (higher r) than using a boundary cell hypothesis, this was classified correctly as a grid cell. (B) As per (A) but data was from a short-range predictive boundary cell, showing that border score, gridness index and parametric rate map correlation coefficients are in agreement that it is a boundary cell. (c) Gridness index[43] vs border score [26] of grid cells (●, n = 8,000) and boundary cells (●, n = 2,640) from 20 recall trials in a 1 m circular arena (including data from (A) and (B)), showing standard threshold values (cyan lines). Probabilistic grid cells (GC) were classified with high sensitivity (sens.) and specificity (spec.), but 31% of predictive boundary cells (BC) were unable to be classified (uncl.). Note that some cells could not be plotted because at least one metric was undefined. Only those boundary cells tuned between 3 and 100 cm were included for analysis, due to arena size constraint and analysis spatial sampling resolution. (D) For the same data as (C), parametric rate map correlations are shown under a boundary vector cell hypothesis, r(Hyp:BVC), and a simplified oscillatory interference grid cell hypothesis, r(Hyp:GC). Unclassified cells (uncl.) were defined as those where both correlation coefficients were below 0.5. (E) As per (A) but in a 1 m square arena with irregular grid axes and grid scales. Normally, this would not be classified as a grid cell (low gridness). In contrast, use of parametric rate map co [file pcbi.1005165.s005.tif]

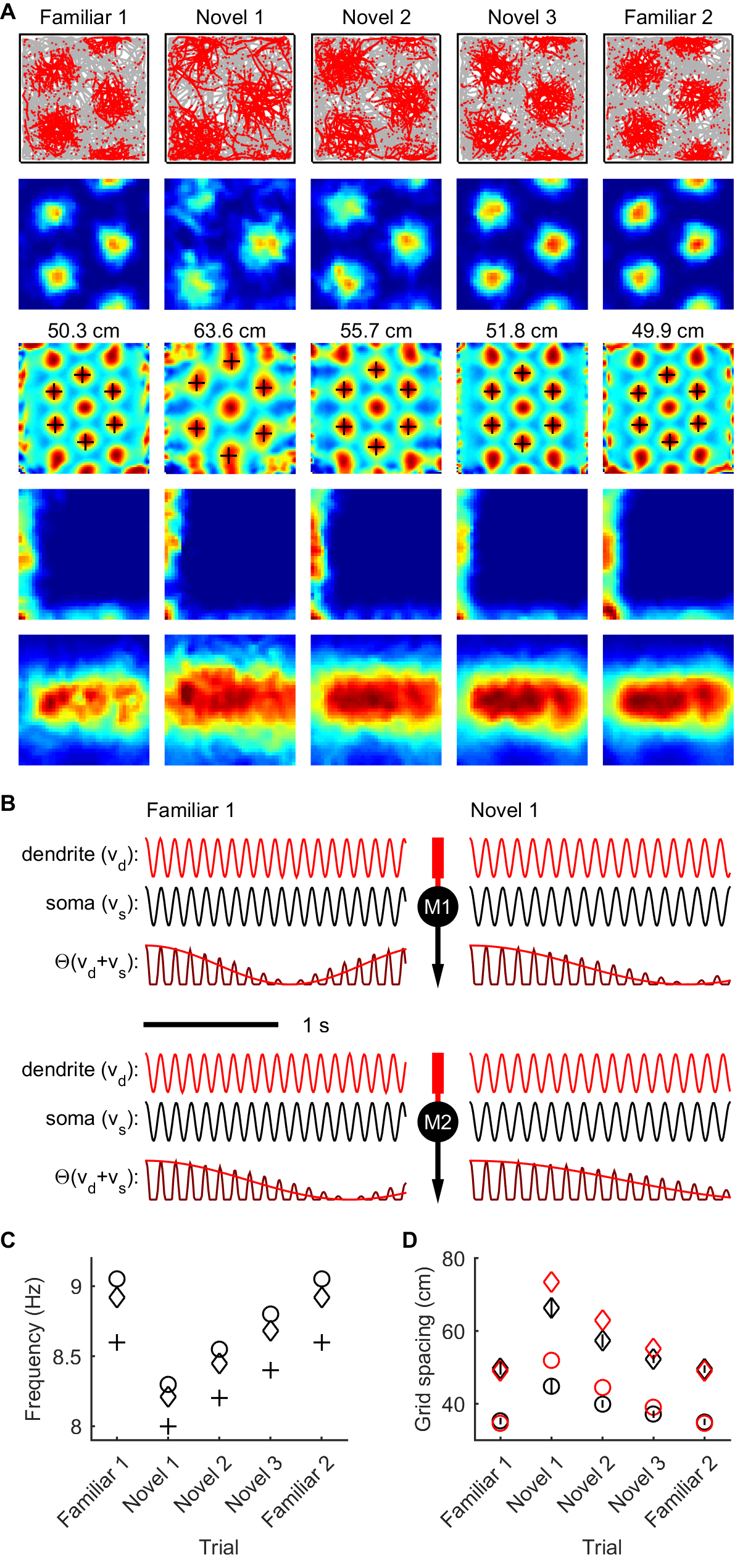

Supplement: S6 Fig — (A) The effect of environmental novelty was modelled as reduced self-motion gain in a 1 m square arena (Novel 1, gain = 6/9; Novel 2, gain = 7/9; Novel 3, gain = 8/9; Familiar 2, gain = 1), following learning (Familiar 1, gain = 1). Grid fields were evident (Novel 1 to 3), showing graded grid expansion (rows 1 to 3). Boundary cell activity also persisted despite conflict with self-motion cues, showing that identical sensory cues and learned information can be used to stabilize multiple distinct grid patterns resulting from reduced self-motion gain. (B) Ideal temporal dynamics of probabilistic grid cells in familiar and novel environments, from grid scale module 1 and 2 (M1 and M2, respectively), with somatic input based on the speed-independent theta frequency reduction in rats exposed to novel environments [47, 55]. (C) Somatic (+) and dendritic (◊, ○) oscillatory frequencies of grid scale module 1 (○) and 2 (◊), corresponding to the reduced self-motion gains of (A). (D) Predicted (◊, ○) and actual (◊, ○) grid spacing (mean ± SD) of probabilistic grid cells diverge in novel environments, showing that oscillatory interference parameters do not fully determine grid scale if probabilistic computations are used. (TIF) [file pcbi.1005165.s006.tif]

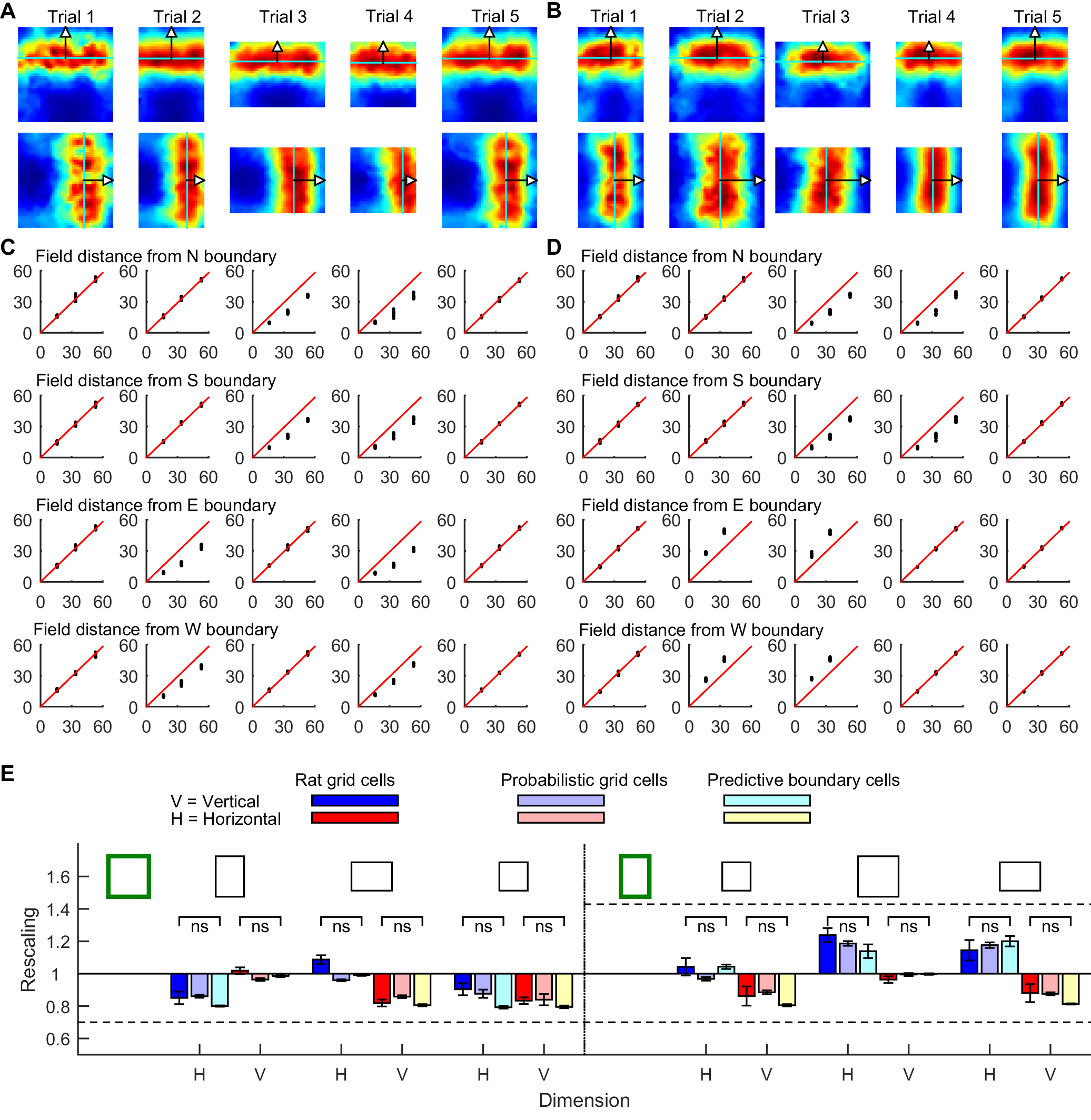

Supplement: S7 Fig — (A and B) Two examples (rows 1 and 2) of predictive boundary cell rate maps from two separate arena resizing series. From boundary cells with tuning directions perpendicular to the boundaries, tuning distances were estimated along the ideal tuning direction (arrow). Field position (─) was estimated by the rate map’s center of mass, thresholded by its mean, i.e., Θ(f−f¯). (C and D) All estimated field distances are shown against their ideal tuning distance. The red line is the equivalence line, adjusted for the wrapped Gaussian angular tuning distribution which reduces the mean perpendicular boundary detection distance, i.e., y = xExp(−σθ2/2) where σθ = π/12 in this model. Arena compression consistently reduced boundary field distances (below the red line) while arena expansion increased field distances (above the red line). (E) Predictive boundary cells with optimal tuning distances of 16.2 and 33.8 cm from the boundary were used to determine how predictive boundary field positions relate to the rescaling magnitude of grid cells. Other tuning distances were excluded because they were either too long for their fields to be within the arena, or too short for their narrow fields to be adequately sampled by the random trajectory and 2 cm spatial bins. Absolute field position along each tuning direction was scaled to both Trial 1 and 5, the average of which was treated as one vertical (collapsing north and south together) or horizontal (collapsing east and west together) rescaling estimate. The magnitude of partial rescaling was statistically indistinguishable from rat grid cells along all twelve dimensions tested (P > 0.05, two-sample t-tests, FDR corrected), demonstrating that predictive boundary field rescaling matches rat grid rescaling during arena resizing trials. (TIF) [file pcbi.1005165.s007.tif]

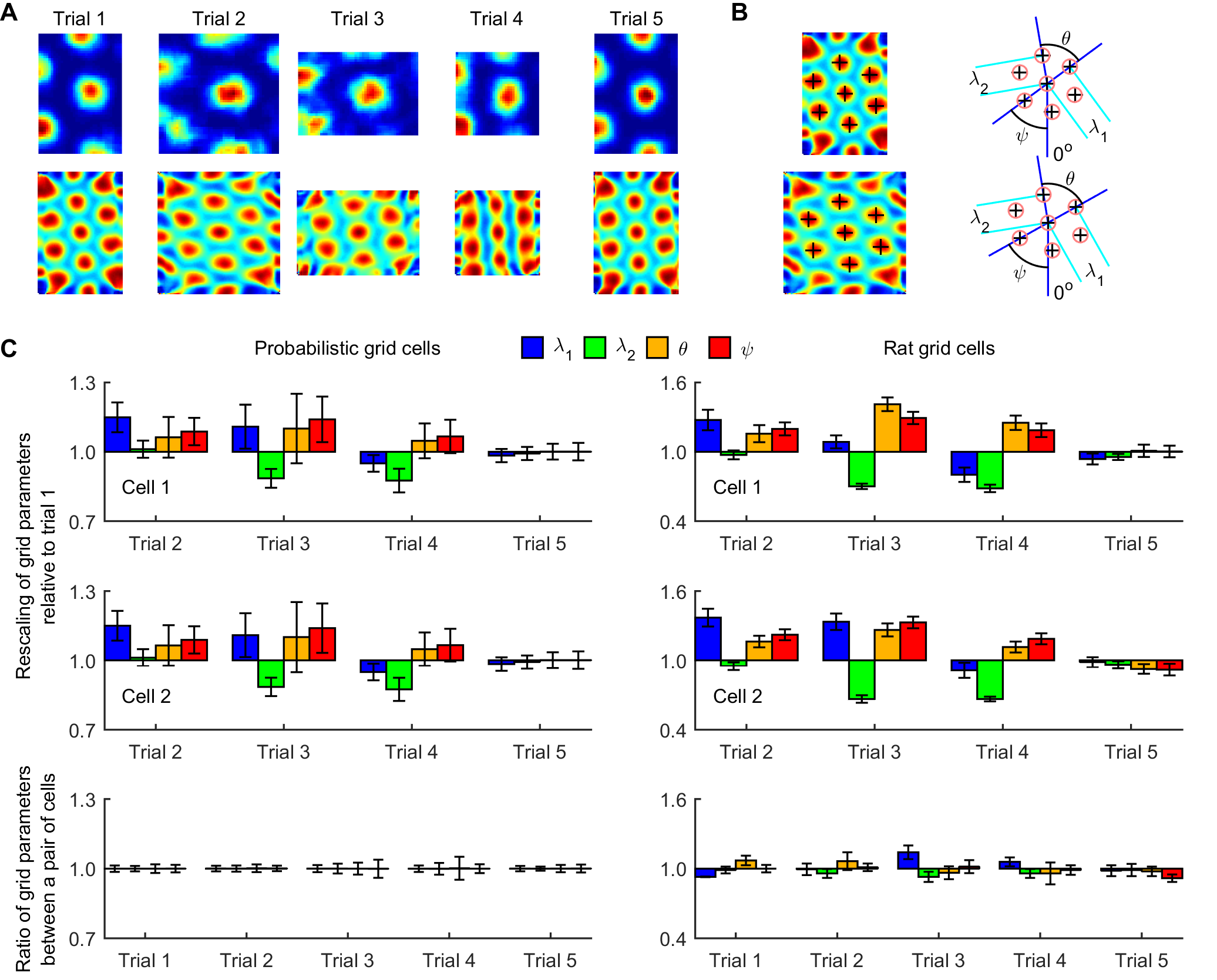

Supplement: S8 Fig — (A) Rate map autocorrelograms for grid cells in a familiar environment (trials 1 and 5) and resized versions of the familiar environment (trials 2–4). (B) The 7 central autocorrelogram peaks were used to find the 4 grid parameters which defined an ideal tessellating grid. The grid axis orientations were chosen to match the convention of Fig 3 of [43]. (C) Grid parameter ratios (mean ± SD) are shown for probabilistic (left) and rat (right) grid cells, comparing parameter rescaling relative to trial 1 (top two rows) in distinct cell pairs within each trial (nominally designated Cell 1 and Cell 2; n = 1,000 probabilistic grid cells per group, n = 100 bootstrap samples from rat data—[43] Online Methods). The similarity between probabilistic grid cell subpopulations reflects the large sample size used. The pattern of relative rescaling of grid parameters were nearly identical between rat and probabilistic grid cells (mean rescaling ratios—rat cell 1 vs model cell 1: r = 0.93, P = 2.6 × 10^−7^; rat cell 2 vs model cell 2: r = 0.98, P = 8.8 × 10^−12^). In both, the unitary ratio of grid parameters between cell pairs within each trial (bottom row: left–model, right—rat) showed grid parameter stability between cells within a trial, despite substantial variability across trials. Note: SIFM grid parameters underestimate the magnitude of rescaling because grid orientations are random, whereas recent analyses suggest that rat grids align closely with one rectangular boundary [11, 44]. (TIF) [file pcbi.1005165.s008.tif]

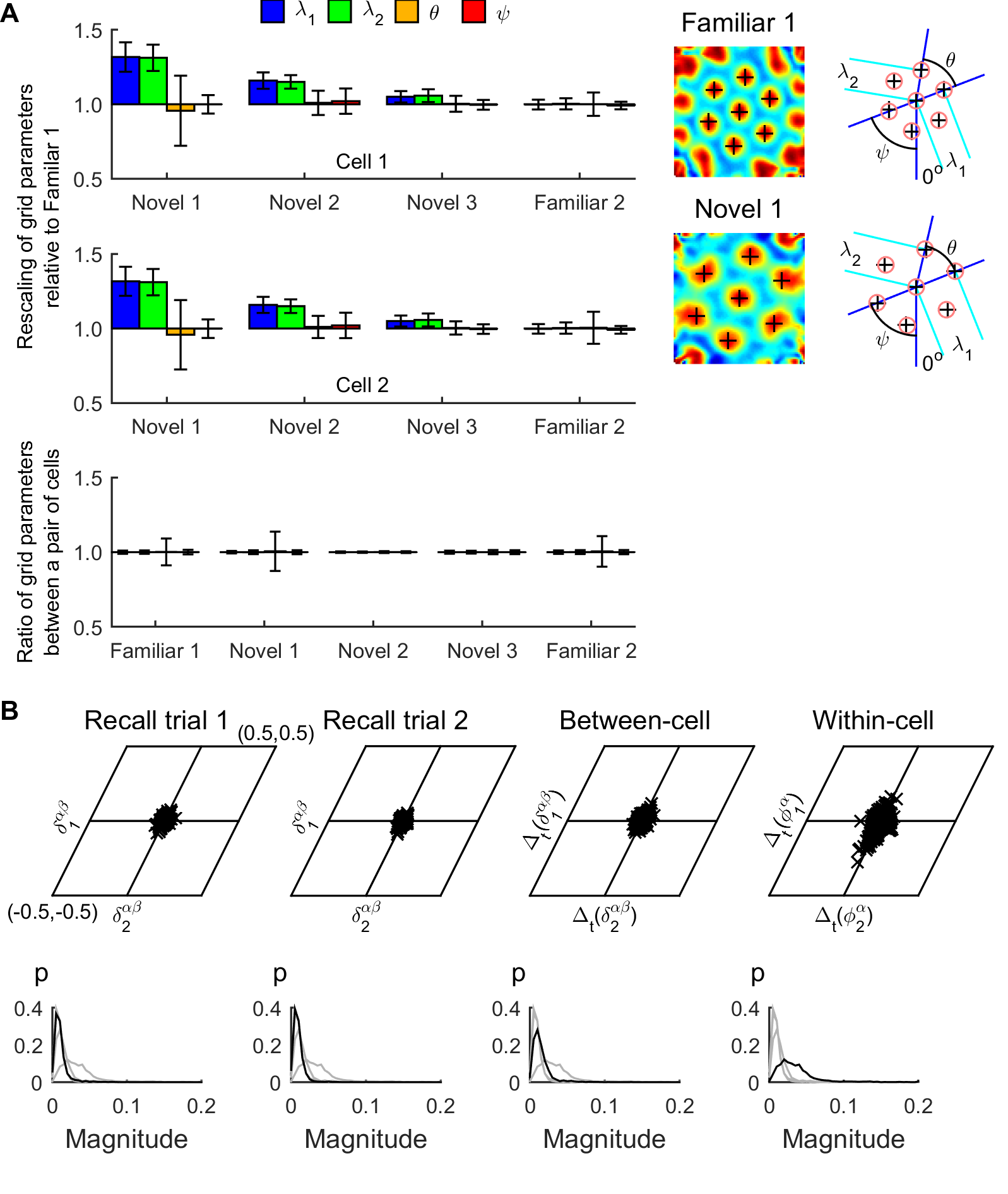

Supplement: S9 Fig — (A) Using template-fitted grid parameters, normalized grid phase differences are compared between cell pairs within a trial (Recall trial 1, Recall trial 2, Between-cell), and within a cell across independent recall trials (Within-cell) in a kite arena in darkness. Phase offsets between cell pairs within each trial (δ1αβ, δ2αβ) can be compared directly because SIFM grid cells are phase-matched. The difference in the phase offset of the corresponding cell pairs across the two recall sessions, i.e., Δt(δ1αβ) and Δt(δ2αβ), showed less variability than within-cell phase changes along the same grid template axes, i.e., Δt(ϕ1α) and Δt(ϕ2α). Below each normalised phase plot, the corresponding probability mass function of the radial magnitude of drift is shown (black line), superimposed on all four probability distributions (grey lines). (B) Using results from S6 Fig which modelled environmental novelty by reducing speed gain, probabilistic grid parameters were fitted as shown on the right (see also S8 Fig and [43]). Relative to the familiar environment (Familiar 1, gain = 1), rescaling of grid parameter ratios (mean ± SD) from distinct cell pairs within each trial (nominally designated Cell 1 and Cell 2; n = 1,000 probabilistic grid cells per group) varied within cells over multiple novel sessions (Novel 1, 2 and 3) despite being in a geometrically-identical arena (1 m square). In contrast, the grid parameter ratios between cells were unchanged (lower panel, close to unity). (TIF) [file pcbi.1005165.s009.tif]

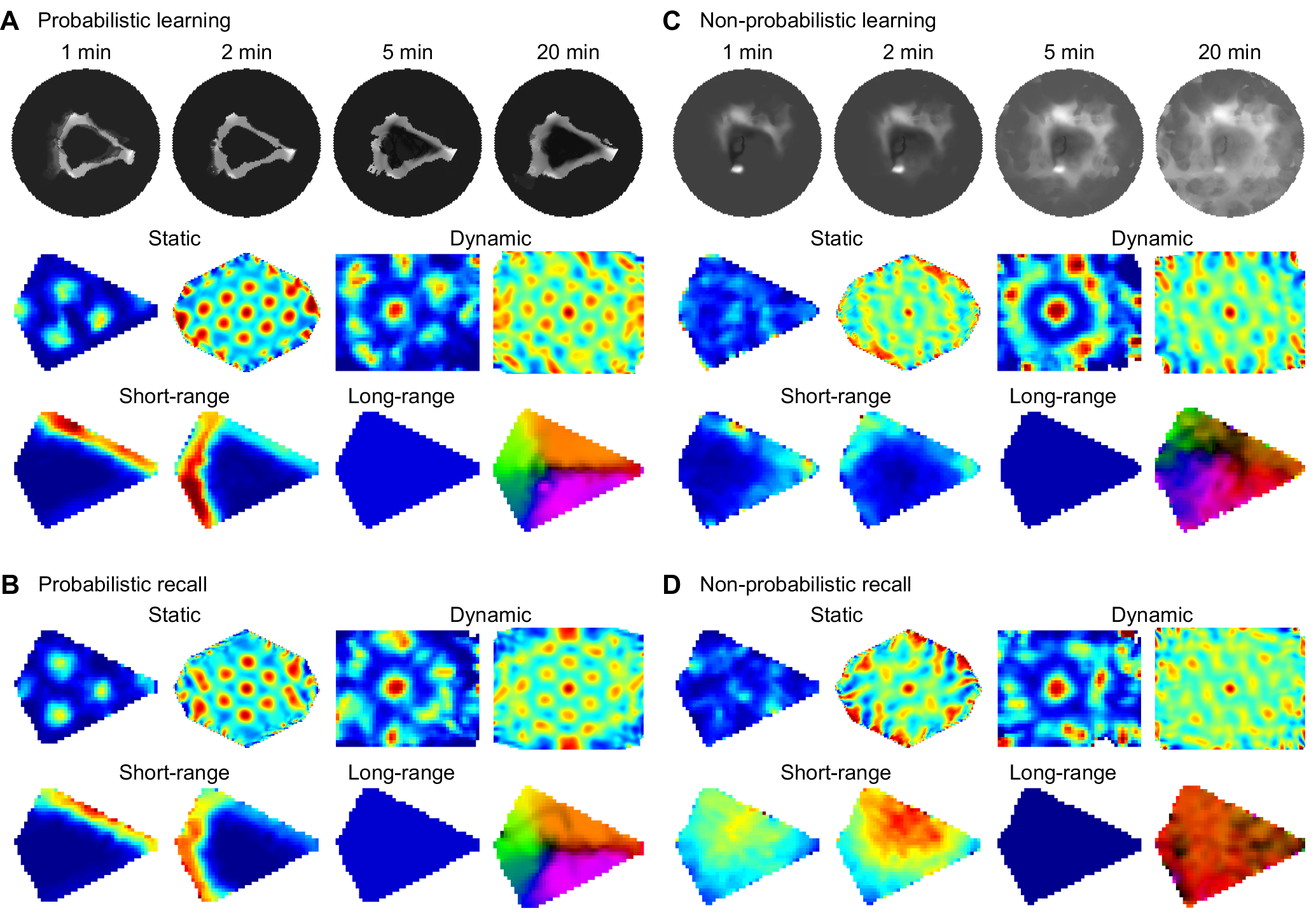

Supplement: S10 Fig — Typical examples of learning (A, C) and recall (B, D) are shown either with (A, B–probabilistic) or without (C, D–non-probabilistic) boundary prediction error feedback. All other model parameters were identical, including distributed grid codes with compensatory phase noise, associative learning between grid and boundary codes, and in darkness. During non-probabilistic learning and recall (C, D), boundary prediction error was set constant to negate its influence on the distribution of grid codes and association maps, impairing probabilistic information fusion and preventing the arena geometry from being learned (C row 1, compared to A row 1). Grids were only evident in standard rate maps and autocorrelograms (static) from probabilistic learning and recall, while spike-triggered dynamic rate maps and autocorrelograms (dynamic [56]) showed some grid-like spatial patterns even during non-probabilistic learning and recall, reflecting the underlying iPI process. Similarly, short-range predictive boundary cells showed inconsistent and dispersed responses without boundary prediction error feedback, leading to loss of oriented structure in boundary vector maps (C, D—lower right, compared to A, B–lower right). Long-range boundary cells were inactive due to lack of vision. (TIF) [file pcbi.1005165.s010.tif]

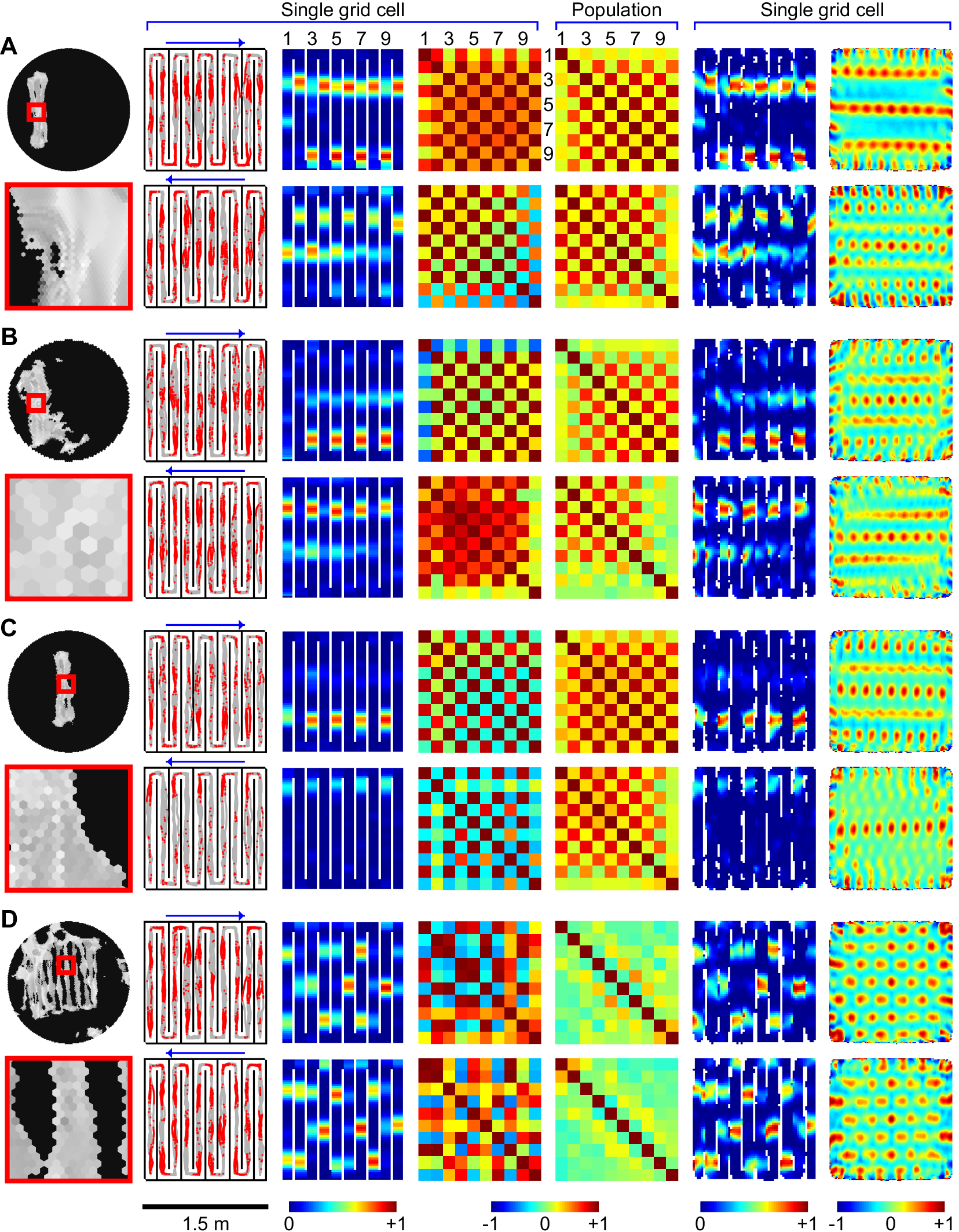

Supplement: S11 Fig — Grid fragmentation in a 1.5 m hairpin maze persisted using association maps with 4-fold (A, halving σg and dmax, adjacent grid codes separated by 1 cm) and 0.25-fold (B, doubling σg and dmax, adjacent grid codes separated by 4 cm) spatial resolution (S1.1.6 Text). The fragmentation pattern of individual grid cells depended on global running direction, forming distinct checkerboard arm-arm correlation matrices. Grid fragmentation also persisted when self-motion linear and angular noise variances were increased 4-fold (C), but was largely abolished when self-motion linear and angular noise variances were decreased to 0.25-fold (D). Under normal to high self-motion noise, the learned spatial layout of the hairpin maze was laterally compressed (A to C, column 1), irrespective of the underlying resolution of the association map (magnified inset), preventing the hexagonal tessellating grid pattern from emerging (columns 6 and 7). Under low self-motion noise, the hairpin maze structure was evident following learning (D, column 1), as were hexagonal tessellating grids (columns 6 and 7). See S2 Table for correlations between arm-arm correlation matrices of probabilistic and rat grid cells. (TIF) [file pcbi.1005165.s011.tif]

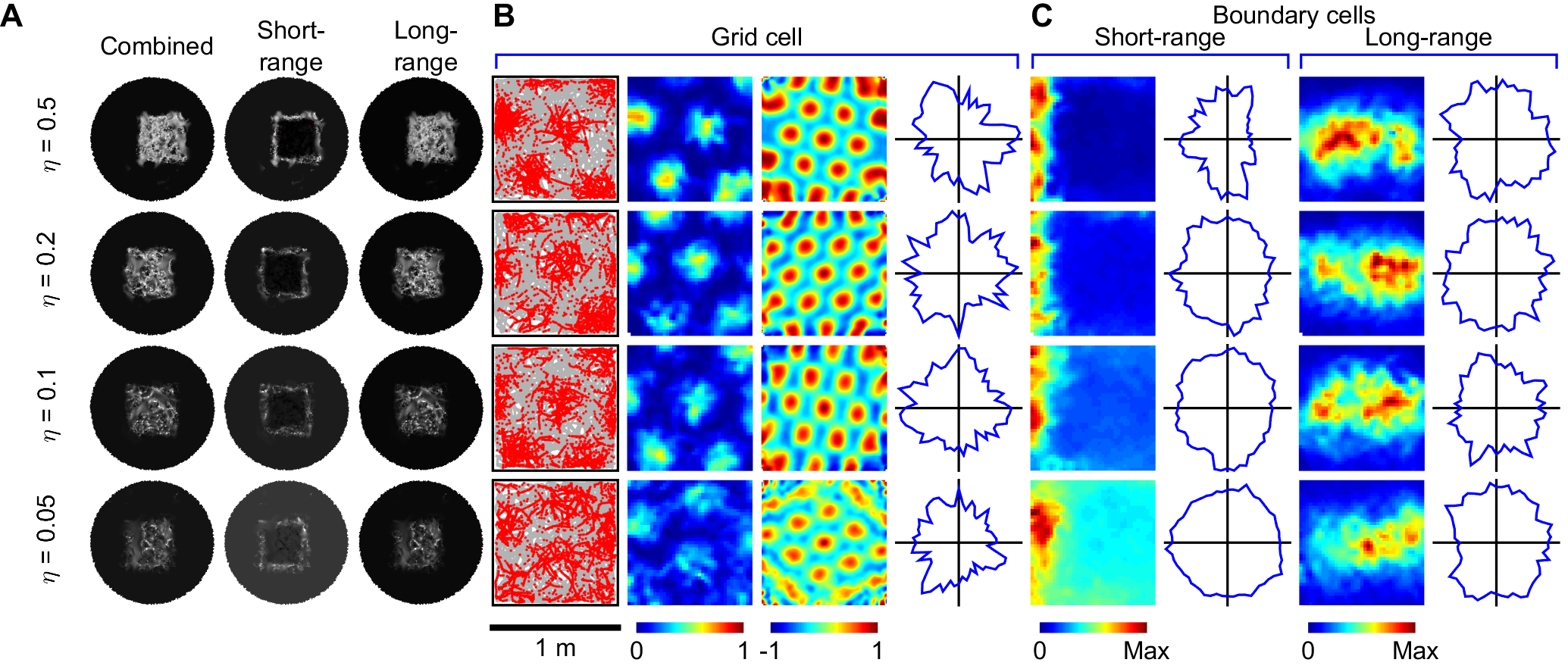

Supplement: S12 Fig — (A) Combined, long-range and short-range association maps following probabilistic learning using a prediction error feedback rule and different learning rates η, initially naïve and with vision. (B) Trajectories and spikes (grey lines, red dots, column 1), firing rate maps (column 2), and rate map autocorrelograms (columns 3) of probabilistic grid cells showing stable grids across an order of magnitude in learning rates. (C) Predictive boundary cells also showed boundary-dependent responses across the same range of learning rates (rate maps, columns 1 and 3). Neither probabilistic grid cells nor predictive boundary cells showed directional-selectivity (directional rate plots, (B) column 4, (C) columns 2 and 4). (TIF) [file pcbi.1005165.s012.tif]
