# Supplementary material for: Probabilistic Learning by Rodent Grid Cells
Source: PLoS Comput Biol. 2016 Oct 28;12(10):e1005165. doi: 10.1371/journal.pcbi.1005165 (PMC5085080; doi:10.1371/journal.pcbi.1005165)
Supplement: S1 Text — (PDF) [file pcbi.1005165.s013.pdf]

Probabilistic learning by rodent grid cells

S1 Text

Author: Allen Cheung[1*]

Affiliation:

[1]The University of Queensland, Queensland Brain Institute, Upland Road, St. Lucia, Queensland, Australia

*Correspondence to: a.cheung@uq.edu.au

Contents

S1.1 Text. Spatial information fusion model (SIFM)

S1.1.1. Theoretical framework

S1.1.2. Oscillatory grid codes

S1.1.3. Noisy self-motion cues

S1.1.4. Noisy boundary codes

S1.1.5. Noisy compass cue

S1.1.6. Associative learning between grid and boundary codes

S1.1.7. Prediction error

S1.1.8. Recall (localization) trials

S1.1.9. Pseudocode for SIFM learning

S1.1.10. Pseudocode for SIFM recall

S1.2 Text. Trajectory models

S1.2.1. Random trajectory

S1.2.2. Thigmotactic bias for learning in darkness

S1.2.3. Hairpin maze

S1.2.4. Total trajectory length was decreased in darkness

S1.3 Text. Data analysis

S1.3.1. Firing rate maps

S1.3.2. Spatial crosscorrelograms

S1.3.3. Gridness index

S1.3.4. Grid rescaling

S1.3.5. Border score

S1.3.6. Parametric rate map correlation

S1.3.7. Weight maps

S1.3.8. Simulated spikes

S1.3.9. Short-range boundary vector maps

S1.3.10. Dynamic grid cell rate maps

S1.3.11. Grid phase change

S1.4 Text. Supporting references

S1.1 Text. Spatial information fusion model (SIFM)

S1.1.1. Theoretical framework

The information fusion principle of SIFM is adapted from probabilistic SLAM (simultaneous localization and mapping). The standard problem of probabilistic SLAM is to estimate the distribution $p\left(x_{1:t}, m \mid z_{1:t}, u_{1:t}\right)$ in high-dimensional state space, where $x_{1:t}$ is the set of poses (position and direction) from step 1 to *t*, *m* is the map of the observable environment, $z_{1:t}$ is the set of external sensory inputs from step 1 to *t*, and $u_{1:t}$ is the set of self-motion estimates from step 1 to *t*. The aim is to approximate the probability distribution of all possible paths and maps given uncertain self-motion and external inputs.

SIFM is implemented as a Rao-Blackwellized particle filter [1-4] which separates the estimation of the trajectory from the learning of associations between anatomically co-localized grid and boundary cells – a set of associations is called a ‘map’ here. This implementation of the probabilistic spatial information fusion principle can directly use and predict information encoded by neuronal responses, is tractable in simulation, and readily understood mathematically. Factorization of the SLAM problem reduces the dimensionality of the state space of all poses and maps to a 3-D state space of pose (two dimensions of position and one of direction), and a conditional term attached to each instantiation of pose. To perform this factorization step in SIFM, associations between each boundary code and grid code were assumed to be independent of grid code. Note that this assumption is an approximation in reality since real boundaries tend to span multiple spatially adjacent grid codes. Nonetheless this step makes the problem computationally tractable and has been found in robotic SLAM models to work well under real-world conditions [2-5]. The state estimation problem can then be factorized into

$$\begin{aligned} p\left(x_{1:t}, m \mid z_{1:t}, u_{1:t}\right) &= p\left(x_{1:t} \mid z_{1:t}, u_{1:t}\right) p\left(m \mid x_{1:t}, z_{1:t}, u_{1:t}\right) \\ &= p\left(x_{1:t} \mid z_{1:t}, u_{1:t}\right) p\left(m \mid x_{1:t}, z_{1:t}\right) \end{aligned} \tag{S1}$$

The last simplification assumes that the map is independent of self-motion cues given the path and environmental inputs are already known.

Since the model was developed to account for the real-time activity of grid cells, only current pose and map information is required so the factorization of (S1) can be rewritten as

$$p(x_t, m_t \mid z_{1:t}, u_{1:t}) = p(x_t \mid z_{1:t}, u_{1:t})\, p(m_t \mid x_{1:t}, z_{1:t}) \tag{S2}$$

Applying a Markov assumption, past self-motion and boundary information are discarded once incorporated into the pose and map estimates, so the factors of (S2) can be written as

$$p(x_t \mid z_{1:t}, u_{1:t}) \approx p(x_t \mid x_{t-1}, z_t, u_t, m_{t-1}) \tag{S3}$$

and

$$p(m_t \mid x_{1:t}, z_{1:t}) \approx p(m_t \mid m_{t-1}, x_t, z_t) \tag{S4}$$

Note that m_t denotes the map which can be estimated by the information acquired up to step *t*, but the true layout of the environment is assumed to be static over time. Applying Bayes theorem to (S3),

$$p(x_t \mid x_{t-1}, z_t, u_t, m_{t-1}) \propto p(z_t \mid x_{t-1}, x_t, u_t, m_{t-1})\, p(x_t \mid x_{t-1}, u_t, m_{t-1}) \tag{S5}$$

From (S5),

$$p(z_t \mid x_{t-1}, x_t, u_t, m_{t-1}) = p(z_t \mid x_t, m_{t-1}) \tag{S6}$$

because the previous pose and most recent self-motion cues have already been incorporated into the pose x_t. Also,

$$p(x_t \mid x_{t-1}, u_t, m_{t-1}) = p(x_t \mid x_{t-1}, u_t) \tag{S7}$$

because pose change is assumed to be independent of the map. Combining (S2) to (S7),

$$p(x_t, m_t \mid z_{1:t}, u_{1:t}) \propto p(z_t \mid x_t, m_{t-1})\, p(x_t \mid x_{t-1}, u_t)\, p(m_t \mid m_{t-1}, x_t, z_t) \tag{S8}$$

In a particle filter implementation of (S8), $p(m_t \mid m_{t-1}, x_t, z_t) \approx 1$, because the map attached to each particle is updated given its pose, so the map is assumed to be conditionally exact. There are abstract worlds for which an exact map update is achievable recursively, e.g., in an environment made of binary blocks, where an occupancy map is used, and all measurements of each block's occupancy are statistically independent.

Empirical evidence shows that violation of these assumptions in real-world environments are well-tolerated [3-5].

The posterior state distribution $p\left(x_t \mid z_{1:t}, u_{1:t}\right) \propto p\left(z_t \mid x_t, m_{t-1}\right) p\left(x_t \mid x_{t-1}, u_t\right)$ is maintained by a set of particles X_t. Each particle, $x_t^{[i]} \in X_t$, represents one hypothesis of pose, and is associated with its own map, $m_t^{[i]}$, which has been constructed stepwise using its private estimate of path $x_{1:t}^{[i]}$ and common external inputs $z_{1:t}$. For example, each particle in SIFM consists of one grid code and its association map. The details of how information encoded by grid and boundary cells are used in SIFM are detailed in subsequent S1 Texts.

Particles are recursively updated using first a prediction step and then a correction step. In the prediction step, the most recent noisy self-motion estimate, u_t, is used to generate a probabilistic estimate of current pose, i.e.,

$$x_t^{[i]} \sim p\left(x_t \mid x_{t-1}^{[i]}, u_t\right) \tag{S9}$$

by sampling from an internal model of self-motion error. This step corresponds to adding random phase noise to a grid code (see later).

In the correction step, the temporary particle set (commonly called the proposal distribution) is resampled according to its importance weight

$$w_t^{[i]} = p\left(z_t \mid x_t^{[i]}, m_{t-1}^{[i]}\right) \tag{S10}$$

such that particles with low importance are likely to be replaced by particles with a high weight. In SIFM, an exact importance weight is not available. For instance, a grid code repeats an infinite times spatially, which makes the exact likelihood of current sensory input given the current grid code (and map) difficult to compute. Instead, the importance weight is approximated by a prediction error term (S1.1.7 Text). A similar surrogate function of the Bayesian likelihood term has been demonstrated previously to be an effective approximation for particle filter implementation on a mobile robot [6]. Stochastic universal resampling [6, 7] is used to normalize the importance, $w_t^{[i]}$, after every step.

Finally, the map, $m_{t-1}^{[i]}$, is updated for each particle [i], based on the common sensory input, z_t, and the pose represented by that particle, $x_t^{[i]}$, i.e.,

$$m_t^{[i]} = \left(m_t^{[i]} \mid m_{t-1}^{[i]}, x_t^{[i]}, z_t \right) \qquad \text{(S11)}$$

where each map is updated according to its corresponding $x_t^{[i]}$. The recursive map update step of (S11) implies that all the map-relevant pose and sensory information have already been incorporated into the previous map, and the latest pose and sensory estimates are sufficient to build on the existing map. This is possible because uncertainty is carried by the distribution of particles so that each particle has the exact map conditional on its pose and sensory history. Adaptations of $x_t^{[i]}$, z_t required to use grid cell and boundary cell responses are described next.

S1.1.2. Oscillatory grid codes

To use and predict grid cell activity in SIFM, pose $x_t^{[i]}$ is replaced by a spatially-repetitive grid code whose properties are based on rodent grid cell data [8],

$$x_t^{[i]} \cong g_t^{[i]} = \begin{bmatrix} f_t^{[i,1]} \\ \vdots \\ f_t^{[i,M]} \\ \phi_t^{[i]} \end{bmatrix} \qquad \text{(S12)}$$

where M = 8 grid scales (modules) unless otherwise stated, and $\phi_t^{[i]}$ denotes the heading orientation associated with grid code [i]. Of note is that directional information is encoded in rodent medial entorhinal cortex as both pure head direction cells and conjunctive grid cells [9], and revealed through hippocampal inactivation in normally directionally-insensitive grid cells [10]. In SIFM, the activity of eight grid cells plus an orientation estimate constitutes each grid code, which is a surjective function of pose (conjunctive position and orientation). Unless otherwise specified (e.g., Fig 8B), a total of 10^3 grid codes are used.

The response of each probabilistic grid cell [i] at time t is determined by the superposition of plane waves along non-parallel directions ϕ_{dj}, i.e.,

$$f_t^{[i]} = \prod_{j=1}^{n_w} \frac{1}{2} \left(\cos\left(\frac{2\pi}{r_j} \sum_{\tau=1}^{t} \lambda_\tau^{[i]} \cos\left(\phi_\tau^{[i]} - \phi_{dj} \right) \right) + 1 \right) \qquad \text{(S13)}$$

where r_j is the spatial period along each direction, $\lambda_\tau^{[i]}$ denotes the linear displacement estimate of grid cell [i] at step τ, and $\phi_\tau^{[i]}$ denotes the heading estimate of grid cell [i] at step τ, noting that $\lambda_\tau^{[i]}$ and $\phi_\tau^{[i]}$ include both sensory and phase noise (see S1.1.3 Text). Unless otherwise specified (e.g., S4 Fig), three plane waves are used (n_w = 3), oriented 60° apart. Spatial periods r_j range from 30 cm to 339 cm along a geometric series increasing by $\sqrt{2}$ per scale module [8]. The grid response, $f_t^{[i]}$, is linearly mapped to between 0 and 1. Grid spatial irregularities [11] can be incorporated by varying the spatial periodicity gain parameter, r_j, or preferred orientation of each dendritic input, ϕ_{dj} [12] (S4 Fig). A non-zero phase offset between pairs of grid cells within a module [13] can also be implemented via additional firing offset during a probabilistic update.

Although the grid response f is not dependent on any particular biophysical model of grid cell activity, one possible interpretation is that it is an approximation of the oscillatory interference model [12, 14, 15] with three dendritic inputs, i.e.,

$$f_t^{[i]} = \Theta\left(\prod_{j=1}^{3}\left(\cos\left(\omega_d t\right) + \cos\left(\omega_s t\right)\right)\right) \tag{S14}$$

where Θ is the Heaviside function, ω_s is the somatic oscillatory frequency, and ω_d is the dendritic oscillatory frequency which depends on the animal's movement speed, s_t, along the preferred direction ϕ_{dj} of dendritic input j:

$$\omega_d = \omega_s + \beta s_t \cos\left(\phi_t^{[i]} - \phi_{dj}\right) \tag{S15}$$

From (S14) and (S15), the interference pattern along direction j oscillates at $\beta/2\pi$ cycles per unit distance. Note that the temporal oscillation frequency of the interference pattern, $\beta s_t \cos\left(\phi_t^{[i]} - \phi_{dj}\right)$, is directly proportional to the speed component along direction j, $s_t \cos\left(\phi_t^{[i]} - \phi_{dj}\right)$, and thus maintains a constant spatial oscillatory period, $2\pi/\beta$, along direction j. Relating back to (S13), over a discrete time step Δt,

$$\beta s_t \Delta t \approx 2\pi\, \lambda_t^{[i]} / r_j \tag{S16}$$

By combining (S13), (S14), (S15) and (S16), testable hypotheses can be generated about local field potential oscillations (e.g., omnidirectional grid expansion in novel environments in S6 Fig).

S1.1.3. Noisy self-motion cues

Self-motion cues, $u_{1:t}$, are assumed to provide noisy angular and linear displacement information, which update grid codes in the same manner as (S9), i.e.,

$$g_t^{[i]} \sim p\left(g_t \mid g_{t-1}^{[i]}, u_t\right) \tag{S17}$$

All grid codes receive a common noisy self-motion input, u_t, suffering from further independent phase noise during updates within each grid code. The sensory angular displacement estimation error, ε_ϕ, is assumed to be Gaussian, i.e.,

$$\Delta\phi + \varepsilon_\phi = \Delta\phi \sim N\left(\Delta\phi, \sigma_\phi^2\right) \tag{S18}$$

where σ_ϕ was estimated previously to be approximately 0.03 radians per step in darkness (at 9/7 steps per s), by modelling the drift in rat head direction cell tuning functions [6, 7, 16, 17]. The linear displacement estimation error, ε_λ, is also assumed to be Gaussian with a coefficient of variation of 0.1, i.e.,

$$\lambda + \varepsilon_\lambda = \lambda \sim N\left(\lambda, \sigma_\lambda^2\right) \tag{S19}$$

and where $\sigma_\lambda = \mu_\lambda / 10$. The independent phase noise is assumed to follow the same distributions so that

$$u_t^{[i]} = \begin{bmatrix} \lambda_t^{[i]} \cos\left(\Delta\phi_t^{[i]}\right) \\ \lambda_t^{[i]} \sin\left(\Delta\phi_t^{[i]}\right) \end{bmatrix} \tag{S20}$$

where $\Delta\phi_t^{[i]} \sim N\left(\Delta\phi_t, \sigma_\phi^2\right)$ and $\lambda_t^{[i]} \sim N\left(\lambda_t, \sigma_\lambda^2\right)$. Since the rotation input, $\Delta\phi_t$, already contains sensory noise, each grid code's heading accumulates both sensory and phase noise, i.e., $\phi_t^{[i]} = \phi_{t-1}^{[i]} + \Delta\phi_t^{[i]}$. Combining (S20) with (S12) and (S13), yields the grid code prediction step of (S17).

In SIFM, the independent phase noise has a compensatory effect by sampling the local space of the noisy displacement estimate $\left(\Delta\phi, \lambda\right)$, so that by chance, some grid codes end up close to the ideal grid code

had there been no sensory noise (others end up further). It is explicitly assumed in SIFM that grid phases across grid cells within each code are tightly correlated. A small amount of non-cumulative independent noise is tolerated within a grid code since association map grid codes learn within a small zone in grid code space. However, cumulative random phase drift between elements of a grid code would lead to SIFM failure because cumulative phase decoupling within each grid code would correspond to large jumps in physical position, unrelated to actual physical displacement. Recent theoretical work demonstrated that autocorrection of grid codes is possible using networks of noisy neurons, whereby cumulative phase decoupling is effectively prevented [18]. Furthermore, both algorithmic and possible neural network implementations have recently been presented which can calculate the physical vector between two grid codes [19]. Therefore, independent grid phase drift which corresponds to an unrealistically large jump (vector magnitude) in physical space can potentially be detected by a similar mechanism, and either corrected or removed (e.g., by incorporating the error into the importance weight in SIFM). Finally, an operational SIFM grid code need not include cells from all grid scale modules (e.g., Fig 8B), which means there could be functional independence between some grid modules [8].

S1.1.4. Noisy boundary codes

In SIFM, boundary information is encoded by two types of boundary cells – sensory and predictive boundary cells. Sensory boundary cells are based on the boundary vector cell (BVC) model [20-23], with some important modifications. First, SIFM boundary input is assumed to be in egocentric coordinates since the animal may not always have a stable compass cue, especially in darkness. The original BVC model assumed that boundaries provide allocentric input to BVC's, and the directional cue was assumed to be from a global compass, possibly via the head direction system. However, the rodent head direction system accumulates error in darkness and drifts randomly [16, 17], which would destabilize BVC response. Instead, SIFM boundary cells use an estimated allocentric direction, $\phi_t^{[i]}$, associated with each grid code rather than a single global direction which may be unstable. Hence modified boundary cell activation, f_{BV}^{Allo}, is

$$f_{BV}^{Allo} \propto \int_{\theta'}\int_{r} \gamma\left(r, \theta' + \phi_t^{[i]}\right) dr d\theta' \qquad \text{(S21)}$$

where θ' denotes the egocentric direction of each detectable boundary point, *r* denotes the distance of the boundary point, and γ denotes the boundary cell's conjunctive distance-and-direction response function [21], i.e.,

$$\gamma(r,\theta) \propto \frac{Exp\left(\frac{-(r-\mu_r)^2}{2\sigma_r^2} + \frac{-(\theta-\mu_\theta)^2}{2\sigma_\theta^2}\right)}{2\pi\sigma_r\sigma_\theta} \tag{S22}$$

The domain of integration in (S21) is the set of egocentric polar coordinates of all boundary points which contribute to a boundary cell's response, which in turn depends on the boundary geometry, the current true pose, availability of vision, and the transparency of boundary walls. The directional Gaussian tuning mean and standard deviation, μ_θ and σ_θ, and radial distance Gaussian tuning mean and standard deviation, μ_r and σ_r, are described in detail below where short range boundary cells are introduced.

To model correlated boundary detection error, zero-mean random Gaussian noise is added to all egocentric boundary vectors during each boundary detection step. This error constitutes a random rotation of all egocentric boundary points, $\varepsilon_\theta^B \sim N\left(0,\sigma_\phi^2\right)$, the same magnitude as self-motion angular noise; and random translations along Cartesian directions, $\varepsilon_x^B \sim N\left(0,0.5^2\right)$ and $\varepsilon_y^B \sim N\left(0,0.5^2\right)$ in units of cm.

For simulation efficiency, a single set of egocentric boundary cell responses are first found using (S22), and used as the environmental sensory input, i.e.,

$$z_t \cong b_t' = \begin{bmatrix} f_{BV}^{Ego[1]} \\ : \\ f_{BV}^{Ego[n]} \end{bmatrix} \tag{S23}$$

where b_t' denotes the egocentric boundary code, consisting of elements $f_{BV}^{Ego[j]}$ which denotes the response of boundary cell [j] to boundary points according to (S22) in egocentric coordinates. The tuning parameters of all boundary cells are described below. Then for each grid code, b_t' is transformed into an estimated allocentric boundary code $\left(b_t^{[i]} \mid b_t', \phi_t^{[i]}\right)$ and compared against the prediction from the grid code and map.

The transform is a circular shift operation (linear interpolation) on the elements of $f_{BV}^{Ego[j]}$ which have the same tuning distance but different tuning directions, i.e.,

$$f_{BV}^{Allo[i,\theta]} = \frac{\angle(\theta_2,\theta)}{\angle(\theta_2,\theta_1)} f_{BV}^{Ego[i,\theta'_1]} + \frac{\angle(\theta,\theta_1)}{\angle(\theta_2,\theta_1)} f_{BV}^{Ego[i,\theta'_2]} \tag{S24}$$

where $\angle(a,b)$ denotes the angular separation of *a* and *b*, $\theta_1 = \theta'_1 + \phi_t^{[i]}$ and $\theta_2 = \theta'_2 + \phi_t^{[i]}$ are the allocentric directions which flank θ and which θ'_1 and θ'_2 map to, respectively, given the estimated heading $\phi_t^{[i]}$. For example, given $\phi_t^{[i]} = 130°$, $f_{BV}^{Allo[i,90°]} = \frac{20}{30} f_{BV}^{Ego[i,-30°]} + \frac{10}{30} f_{BV}^{Ego[i,-60°]}$. Hence an allocentric boundary code is estimated from a common set of egocentric boundary cell responses and the estimated heading $\phi_t^{[i]}$ of each grid code, i.e.,

$$\left(b_t^{[i]} \mid b_t', \phi_t^{[i]}\right) = \begin{bmatrix} f_{BV}^{Allo[i,1]} \\ \vdots \\ f_{BV}^{Allo[i,n]} \end{bmatrix} \tag{S25}$$

where $f_{BV}^{Allo[i,j]}$ denotes the response of allocentric sensory boundary cell [*j*] associated with grid code [*i*]. Notably, sensory boundary cells need to develop before SIFM grid cells can show stable grids, consistent with recent rodent developmental results [24].

In contrast, predictive boundary cell responses depend on both grid cell response and learned associative weights, i.e.,

$$b_t^{[i,j]} \mid g_t^{[i]}, m_{t-1}^{[i]} = \sum_k \alpha_t^{[i,k]} g_t^{[i]} W_{t-1}^{[i,j,k]} \tag{S26}$$

and can therefore deviate from sensory boundary cell response $\left(b_t^{[i]} \mid b_t', \phi_t^{[i]}\right)$, and should mature along with grid cells (see S1.1.6 Text). Successful learning in a stable environment leads to convergence of sensory and predictive boundary cell responses, i.e., $\left(b_t^{[i]} \mid b_t', \phi_t^{[i]}\right) \approx \left(b_t^{[i]} \mid g_t^{[i]}, m_{t-1}^{[i]}\right)$. For simplicity, head direction is assumed to be aligned with the heading ϕ. Otherwise, an extra head rotation term is required to estimate sensory boundary cell activation, e.g., $\left(b_t^{[i]} \mid b_t', \phi_t^{[i]} + \Delta\phi_t^{Head}\right)$. Since predictive boundary cells are unique to SIFM, their responses are stored for further analysis. Sensory boundary cell responses are not stored for

further analysis since their properties are virtually indistinguishable from boundary vector cells described previously [20, 21, 23]. Due to computer memory limitations, the response of each cell is averaged over all grid codes and maps. The latter reduces rate variability but otherwise produces similar boundary cell rate maps because both association maps and grid codes converge over time.

In addition to long-range boundary cells of the BVC model, SIFM includes a population of short-range boundary cells whose tuning distances are appropriate for boundary detection in darkness. The latter is assumed to have a maximum range of 7 cm, based on the longest rat whiskers being on average 6 cm [25] and head width and head movement adding a small radial range. A circular (panoramic) detection region is assumed, ignoring more extensive boundary detection contributions which may arise from the body and tail which trail significantly more than 7 cm behind the rat's head. The short-range boundary cell tuning distances are $\mu_r^{SHORT} \in \{0.5, 1.5, 2.5, 3.5, 4.5, 5.5, 6.5, 7.5, 8.5\}$ cm, with constant tuning standard deviation σ_r^{SHORT} = 0.5 cm. Long-range boundary cell tuning distances are from model BVCs originally fitted to best account for hippocampal place fields[21]. The fitted tuning distances are $\mu_r^{LONG} \in \{16.2, 33.8, 53.0, 73.8, 96.5, 121.3, 148.2, 177.4, 209.3, 244.0\}$ cm, and the tuning standard deviations are given by $\sigma_r^{LONG} = \sigma_0 \left(\mu_r^{LONG} / 183cm + 1 \right)$ where σ_0 = 12 cm, taken from from the BVC model.

Long-range boundary cell responses are assumed to be vision-dependent [26], and therefore limited by visual field [27] so only boundary points in the frontal 270° are used (limiting the domain of integration of θ' in (S21)). Prediction error (see later) is similarly assumed to be limited for the long-range boundary cells corresponding to the frontal 270°.

The directional Gaussian tuning parameters of SIFM boundary cells are $\mu_\theta \in \{0, 30, 60, 90, 120, 150, 180, 210, 240, 270, 300, 330\}°$, and σ_θ = 15°, resulting in a total of 228 distinct boundary cell distance-direction pairs. Absolute direction is arbitrary in SIFM since initial grid orientations and grid phases are randomly assigned. However, for consistency and presentation clarity, 0° is aligned with the X-axis at the beginning of each learning trial, without loss of generality. Cumulative errors can lead to drift in map position and orientation, the latter especially in the absence of a compass.

During foraging, rats whisk at approximately 4-6 cycles per second [28], corresponding to approximately 4 whisks per step in darkness [6, 29]. For simplicity, a constant 4 boundary observations are modelled per step. Boundary information is learned once per step, immediately following the generation of the prediction error, based on (S10) and (S11).

S1.1.5. Noisy compass cue

Rodent head direction cell tuning is stable in the presence, but not absence, of vision [16]. A coarse compass is therefore assumed to be used specifically with vision. Compass error is assumed to be Gaussian, i.e., $\phi_{HD} \sim N\left(\phi, \sigma_{HD}^2\right)$, where σ_{HD} is liberally estimated to be 45°, an upper limit based on the width of head direction cell tuning functions [30]. The compass cue independently modulates the prediction error signal (S1.1.7 Text):

$$\varepsilon_{HD}^{[i]} = \left(\frac{\phi_t^{[i]} - \phi_{HD}}{\sigma_{HD}} \right)^2 \tag{S27}$$

In the absence of vision, $\varepsilon_{HD}^{[i]} = 0$.

S1.1.6. Associative learning between grid and boundary codes

Each associative map element is updated according to the current grid code and sensory boundary information, based on (S11). In robotic probabilistic SLAM using occupancy maps, the map is typically assumed to be composed of independent locations, each either occupied or not. Map update then involves updating the estimated probability that each location is occupied (Chapter 9 of [5]). In SIFM, the boundary code is not readily transformed into occupancy. Instead, the boundary code itself is learned. Learning is restricted to a region in grid code space defined by a metric of distance between the current grid code and each map element. The update weight from grid code [*i*] to map element [*k*] is defined by a truncated Gaussian function:

$$\alpha_t^{[i,k]} = \left\{ \begin{array}{cc} Exp\left(\dfrac{-\sum_{c=1}^{M}\left(g_t^{[i,c]} - g_m^{[k,c]}\right)^2}{2\sigma_g^2} \right) & if \quad \sum_{c=1}^{M}\left(g_t^{[i,c]} - g_m^{[k,c]}\right)^2 < d_{\max} \\ 0 & otherwise \end{array} \right\} \tag{S28}$$

where M is the number of grid scales (modules) of grid cells (see S1.1.2 Text), and $g_m^{[k,c]}$ is the grid code of association map element k at module c. Hence there is a small zone in grid code space within which each map grid code can learn boundary associations. Unless otherwise specified, $d_{\max} = 0.01$ and $\sigma_g = 0.005$, and association map grid codes are defined according to (S13) without noise, corresponding to a regular hexagonal spatial grid of scale 2 cm. Empirically, these values balance the need for grid codes to cover the traversable physical space, while maintaining sufficient spatial resolution for effective spatial learning. Although (S28) is efficient and straightforward to use in simulation, the distance between two grid codes can also be extracted using neural networks, via either rate- or phase- coded vector cell implementations [19]. Next, the resulting set of update weights for each grid code is normalized so that the total map update weight for any grid code [i] remains constant, i.e.,

$$\sum_k \alpha_t^{[i,k]} = 1 \tag{S29}$$

Finally, for grid code [i], the associative weight between map grid code [k] and predictive boundary cell [j] is updated according to

$$W_t^{[i,k,j]} = \frac{\alpha_t^{[i.k]}\left(b_t^{[i,j]} \mid b_t{}', \phi_t^{[i]}\right) + A_{t-1}^{[i,k]} W_{t-1}^{[i,k,j]}}{A_t^{[i,k]}} \tag{S30}$$

where

$$A_t^{[i,k]} = A_{t-1}^{[i,k]} + \alpha_t^{[i,k]} \tag{S31}$$

At the beginning of learning, $W_0^{[i,j,k]} = 0.1$, which can be increased or decreased through learning, analogous to the prior probability of occupancy maps in probabilistic SLAM [2-5]. Since $b_t^{[i,j]} \mid b_t{}', \phi_t^{[i]} \geq 0$, $W_t^{[i,k,j]} \geq 0$. For simplicity, associative learning is modelled directly between grid codes and boundary cells. Future

development of SIFM may involve grid cells activating a population of intermediate cells, such as place cells, which then learn associations with boundary cells. Note that during recall trials, no learning occurs (S1.1.8 Text).

The use of a cumulative learning record in (S31) allows a simple arithmetic mean of the sensory boundary code to be learned recursively using (S30). While this mathematically simple learning rule ensures transparency regarding the information which is learned, it is not essential for probabilistic learning *per se*. For example, an alternative simple learning based on a cell-wise boundary prediction error can also be used which does not require (S31), i.e.,

$$W_t^{[i,j,k]} = W_{t-1}^{[i,j,k]} + \eta \alpha_t^{[i.k]} \left(\left(b_t^{[i,j]} \mid b_t', \phi_t^{[i]} \right) - \left(b_t^{[i,j]} \mid g_t^{[i]}, m_{t-1}^{[i]} \right) \right) \tag{S32}$$

where η denotes the learning rate. Empirically, η ranging from 0.05 to 0.5 can produce stable grids over 20 minutes of probabilistic learning, initially naïve (S12 Fig).

S1.1.7. Prediction error

Applying the probabilistic SLAM framework to the elements of SIFM, the importance weight (S10) assigned to each grid code should be based on the likelihood of the sensory boundary code given the current grid code and association map. In practice, an exact likelihood function is difficult to define except under certain assumptions about the environment such as the world being made up of independent blocks which are either occupied or not (e.g., Chapter 9 of [5]). Furthermore, computations such as the estimation of an occupancy code from a boundary code can be difficult to implement either neurally or algorithmically. Hence a simple alternative likelihood function is used in SIFM. Since a predictive boundary code is a function of the current grid code and association map, a boundary prediction error can be defined which is inversely related to the importance weight, i.e.,

$$SSE = \sum_j \left(\left(b_t^{[i,j]} \mid g_t^{[i]}, m_{t-1}^{[i]} \right) - \left(b_t^{[i,j]} \mid b_t', \phi_t^{[i]} \right) \right)^2 \tag{S33}$$

where $\left(b_t^{[i,j]} \mid b_t', \phi_t^{[i]}\right)$ denotes the response of sensory boundary cell [*j*] in estimated allocentric coordinates of grid code [*i*], and $\left(b_t^{[i,j]} \mid g_t^{[i]}, m_{t-1}^{[i]}\right)$ denotes the response of predictive boundary cell [*j*], which is a function of the current grid code, $g_t^{[i]}$, and map, $m_{t-1}^{[i]}$ (see (S26)). The importance weight of (S10) is then approximated as

$$w_t^{[i]} = Exp\left(-\frac{SSE + \varepsilon_{HD}^{[i]}}{2}\right) \tag{S34}$$

and used for stochastic universal resampling following each step [6, 7, 31]. The culling of grid codes and association maps with high prediction error is coupled with cloning of those with low prediction error, thereby preserving a stable population of active grid cells.

It is worth noting that the resampling step in a particle filter implementation is a practical measure to prevent particle weight degeneracy, and not a fundamental requirement of information fusion. Without resampling when using finite particle populations, very few particles end up with most of the importance weight and cannot adequately sample the state space of interest. In SIFM, the useful population of grid codes and association maps would dwindle, progressively reducing the accuracy of the representation of uncertainty and hence information fusion itself. In contrast, if the size of a particle population is unlimited (or at least very large relative to the duration of learning), then the cumulative importance weight suffices to carry the necessary information about which particles are accurate, and which are not. Then resampling is not necessary since sufficient particles will remain near the correct state space despite being a decreasing fraction of the total.

S1.1.8. Recall (localization) trials

After learning, recall (localization) tests are assumed to occur with a frozen map averaged over all grid codes. This sped up simulations by at least an order of magnitude, allowing multiple trials to be carried out across different recall conditions. Freezing the map also has the advantage of preventing any possibility of performance variability being affected by continued learning (e.g., independent recall trials in darkness).

Unless otherwise specified, all recall trials began initially disoriented, modelled as a random distribution of grid codes over the learned map where weights had either increased >150% or decreased to <50% of the baseline weight $W_0^{[i,j,k]}$.

Note that for 10-minute localization trials in 1 m circular arenas in darkness, animals were assumed to use the last available set of grid codes in light at the beginning of the darkness trial. In the original experiment [13], lights were switched off without disturbing the animal, allowing the last pose estimate in light to be used at the beginning of the darkness period.

S1.1.9. Pseudocode for SIFM learning

The following pseudocode summarizes the key steps in implementing SIFM for probabilistic learning, initially naïve.

Model initialization

Grid cells

Number of grid cells per grid code (e.g., 8)

Directions and phase offset of each oscillatory component (e.g., three directions at 0°, 60° and 120°, with zero offset)

Boundary cells

Egocentric direction tuning functions (μ_θ, σ_θ)

Distance tuning functions (μ_r^{SHORT}, μ_r^{LONG}, σ_r^{SHORT}, σ_r^{LONG})

Association maps (one per grid code)

Predefine map grid codes and update range function ($g_m^{[k,c]}$, $\alpha_t^{[i,k]}$ and $\sum_{c=1}^{M}\left(g_t^{[i,c]} - g_m^{[k,c]}\right)^2 < d_{\max}$)

Set prior association weights ($W_0^{[i,j,k]}$)

Noise

Self-motion ($\varepsilon_{\phi} \sim N\left(0,\sigma_{\phi}^{2}\right)$, $\varepsilon_{\lambda} \sim N\left(0,\sigma_{\lambda}^{2}\right)$)

Boundary detection ($\varepsilon_{\theta}^{B} \sim N\left(0,\sigma_{\phi}^{2}\right)$, $\varepsilon_{x}^{B} \sim N\left(0,\left(\sigma_{x}^{B}\right)^{2}\right)$, $\varepsilon_{y}^{B} \sim N\left(0,\left(\sigma_{y}^{B}\right)^{2}\right)$)

Compass (when available, $\phi_{HD} \sim N\left(\phi,\sigma_{HD}^{2}\right)$)

Sensory limits

Boundary detection radial range in darkness, e.g., 7 cm whisking range

Boundary detection angular range using vision, e.g., frontal 270 degrees

Boundary detection cycles per step, e.g., 4 whisks per step

Boundary detection angular sampling resolution, e.g., 2°

Arena definition

Arena geometry

e.g., rectangle of length 100 cm and width 70 cm

Arena wall transparency

e.g., semitransparent hairpin maze (ST), modelled by letting boundary cells respond to both local walls, as well as outer perimeter walls which are otherwise obstructed by local walls

Trajectory definition

Random trajectory (approximately uniform coverage of 2D spaces)

Thigmotactic trajectory (concentrated coverage near outer boundary)

Path-following (hairpin and radial arm mazes)

Note the trajectory length is longer with vision (e.g., 50% larger t_{max}).

Probabilistic learning

For t = 1 to t_{max}

Noisy self-motion inputs:

$$\Delta\phi \sim N\left(\Delta\phi, \sigma_\phi^2\right)$$

$$\lambda \sim N\left(\lambda, \sigma_\lambda^2\right)$$

Noisy boundary inputs: egocentric distance and direction vectors of detectable boundary points (detectability depends on arena geometry, wall transparency, and availability of vision)

Update grid codes using self-motion input and compensatory phase noise:

$$u_t^{[i]} = \begin{bmatrix} \lambda_t^{[i]} \cos\left(\Delta\phi_t^{[i]}\right) \\ \lambda_t^{[i]} \sin\left(\Delta\phi_t^{[i]}\right) \end{bmatrix}$$

$$f_t^{[i]} \approx \prod_{j=1}^{3} \frac{1}{2}\left(\cos\left(\frac{2\pi}{r_j} \sum_{\tau=1}^{t} \left\| u_\tau^{[i]} \right\| \cos\left(\phi_t^{[i]} - \phi_{dj}\right) \right) + 1 \right)$$

Define activity of sensory boundary cells using boundary inputs (for all boundary detection cycles within each step), using an intermediate egocentric reference frame:

$$\gamma(r,\theta) \propto \frac{Exp\left(\frac{-(r-\mu_r)^2}{2\sigma_r^2} + \frac{-(\theta-\mu_\theta)^2}{2\sigma_\theta^2} \right)}{2\pi\sigma_r\sigma_\theta}$$

$$f_{BV}^{Ego} \propto \int_{\theta'}\int_{r} \gamma(r,\theta')\, dr\, d\theta'$$

$$b_t' = \begin{bmatrix} f_{BV}^{Ego[1]} \\ \vdots \\ f_{BV}^{Ego[n]} \end{bmatrix}$$

Transform into allocentric boundary cell response:

$$\left(b_t^{[i]} \mid b_t', \phi_t^{[i]}\right) = \begin{bmatrix} f_{BV}^{Allo[i,1]} \\ \vdots \\ f_{BV}^{Allo[i,n]} \end{bmatrix}$$

Note that defining boundary cell activities in an intermediate egocentric frame increases simulation efficiency, but is not required for model function.

Noisy compass direction estimate when vision is available

$$\phi_{HD} \sim N\left(\phi, \sigma_{HD}^2\right)$$

Predict boundary cell activity from each grid code and corresponding association map

$$b_t^{[i,j]} \mid g_t^{[i]}, m_{t-1}^{[i]} = \sum_k \alpha_t^{[i,k]} g_t^{[i]} W_{t-1}^{[i,j,k]}$$

Find boundary prediction error of each grid code

$$SSE = \sum_j \left(\left(b_t^{[i,j]} \mid g_t^{[i]}, m_{t-1}^{[i]} \right) - \left(b_t^{[i,j]} \mid b_t', \phi_t^{[i]} \right) \right)^2$$

$$\varepsilon_{HD}^{[i]} = \left(\frac{\phi_t^{[i]} - \phi_{HD}}{\sigma_{HD}} \right)^2$$

Calculate the importance weight of each grid code

$$w_t^{[i]} = Exp\left(-\frac{SSE + \varepsilon_{HD}^{[i]}}{2} \right)$$

Resample grid codes and the paired association maps

Update association maps using the new grid codes and sensory boundary information over the last step

$$W_t^{[i,k,j]} = \frac{\alpha_t^{[i.k]} \left(b_t^{[i,j]} \mid b_t', \phi_t^{[i]} \right) + A_{t-1}^{[i,k]} W_{t-1}^{[i,k,j]}}{A_t^{[i,k]}}$$

$$A_t^{[i,k]} = A_{t-1}^{[i,k]} + \alpha_t^{[i,k]}$$

End for

See SIFM_ProbabilisticLearning.m (S1 Code) for an example in Matlab.

S1.1.10. Pseudocode for SIFM recall

The following pseudocode summarizes the key steps in implementing SIFM for probabilistic recall, using information learned previously.

Model initialization

The parameters of grid cells, boundary cells, association maps, noise, compass, and sensory limits were the same as the final step of the last learning trial.

When initially disoriented, grid codes were randomly chosen from association map grid codes whose mean associative weight to boundary cells exceeded a change threshold (measured as the absolute difference from W_0), as a marker of learning. When initially oriented, grid codes were set to the last oriented values.

Due to computer storage limitations, only an average association map was kept at the end of each learning trial. Since no learning occurred during strictly recall trials, the same single association map was used for all grid codes during recall.

Arena definition

Arena properties during recall trials may differ from learning.

Trajectory definition

Trajectory properties during recall trials may differ from learning.

Probabilistic recall

For t = 1 to t_{max}

Noisy self-motion inputs:

$$\Delta\phi \sim N\left(\Delta\phi, \sigma_\phi^2\right)$$

$$\lambda \sim N\left(\lambda, \sigma_\lambda^2\right)$$

Noisy boundary inputs: egocentric distance and direction vectors of detectable boundary points (detectability depends on arena geometry, wall transparency, and availability of vision)

Update grid codes using self-motion input and compensatory phase noise:

$$u_t^{[i]} = \begin{bmatrix} \lambda_t^{[i]} \cos\left(\Delta\phi_t^{[i]}\right) \\ \lambda_t^{[i]} \sin\left(\Delta\phi_t^{[i]}\right) \end{bmatrix}$$

$$f_t^{[i]} \approx \prod_{j=1}^{3} \frac{1}{2}\left(\cos\left(\frac{2\pi}{r_j} \sum_{\tau=1}^{t} \left\| u_\tau^{[i]} \right\| \cos\left(\phi_t^{[i]} - \phi_{dj}\right) \right) + 1 \right)$$

Define activity of sensory boundary cells using boundary inputs (for all boundary detection cycles within each step), using an intermediate egocentric reference frame:

$$\gamma(r,\theta) \propto \frac{Exp\left(\frac{-(r-\mu_r)^2}{2\sigma_r^2} + \frac{-(\theta-\mu_\theta)^2}{2\sigma_\theta^2} \right)}{2\pi\sigma_r\sigma_\theta}$$

$$f_{BV}^{Ego} \propto \int_{\theta'}\int_{r} \gamma(r,\theta')\, dr\, d\theta'$$

$$b_t' = \begin{bmatrix} f_{BV}^{Ego[1]} \\ : \\ f_{BV}^{Ego[n]} \end{bmatrix}$$

Transform into allocentric boundary cell response:

$$\left(b_t^{[i]} \mid b_t', \phi_t^{[i]}\right) = \begin{bmatrix} f_{BV}^{Allo[i,1]} \\ : \\ f_{BV}^{Allo[i,n]} \end{bmatrix}$$

Note that defining boundary cell activities in an intermediate egocentric frame increases simulation efficiency, but is not required for model function.

Noisy compass direction estimate when vision is available

$$\phi_{HD} \sim N\left(\phi, \sigma_{HD}^2\right)$$

Predict boundary cell activity from each grid code and corresponding association map

$$b_t^{[i,j]} \mid g_t^{[i]}, m_{t-1}^{[i]} = \sum_k \alpha_t^{[i,k]} g_t^{[i]} W_{t-1}^{[i,j,k]}$$

Find boundary prediction error of each grid code

$$SSE = \sum_j \left(\left(b_t^{[i,j]} \mid g_t^{[i]}, m_{t-1}^{[i]} \right) - \left(b_t^{[i,j]} \mid b_t', \phi_t^{[i]} \right) \right)^2$$

$$\varepsilon_{HD}^{[i]} = \left(\frac{\phi_t^{[i]} - \phi_{HD}}{\sigma_{HD}} \right)^2$$

Calculate the importance weight of each grid code

$$w_t^{[i]} = Exp\left(-\frac{SSE + \varepsilon_{HD}^{[i]}}{2} \right)$$

Resample grid codes

End for

See SIFM_ProbabilisticRecall.m (S1 Code) for an example in Matlab.

S1.2 Text. Trajectory models

All learning trials presented were 20 minutes in duration, comparable to training sessions in rodent experiments [8, 9, 11, 13, 32-35]. This duration was long enough to adequately sample the arena to calculate rate maps for grid cells and boundary cells, while being short enough to simulate multiple independent trials in reasonable computing time (minimum of 10 trials per condition). Subsequent test trial durations were based on published experiments [13, 32, 34]. Trajectory positions were assumed to correspond to the center of a rat's head, which cannot physically touch any boundary wall and assumed to be no closer than 2 cm from the nearest boundary.

S1.2.1. Random trajectory

A random trajectory was used for rapid coverage of the entire space of each 2D arena [7]. This trajectory model was used for learning and recall trials with vision, providing complete sampling of the traversable space. Pseudocode used to model true displacement $(\Delta\phi_t, \lambda_t)$, starting at pose y_t for a random trajectory at time step *t* is shown below.

Pseudocode begins

Randomly draw angular displacement $\Delta\phi_t \sim \mathrm{WN}\left(0, \sigma_\tau^2\right)$

KeepSearching = true

k=0

While KeepSearching = true **do**

    Randomly draw linear displacement $\lambda_t \sim \mathrm{N}\left(\mu_\lambda, \sigma_\lambda^2\right)$

    If displacement $(\Delta\phi_t, l_t)$ from current pose y_t crosses a region within 2 cm of any boundary

        k=k+1

        If RandomNumber > 0.5

            $\tau \sim \mathrm{WN}\left(0, \sigma_\tau^2\right)$

$$\Delta\phi_t = \Delta\phi_t + \tau(1.1)^k$$

Else

$$\tau \sim \mathrm{WN}\left(0, 25\sigma_\tau^2\right)$$

$$\Delta\phi_t = \Delta\phi_t + \tau(1.1)^k$$

End

Else

KeepSearching = false

End

End

$$y_{t+1} = y_t + \begin{bmatrix} \lambda_t \cos\left(y_t(1) + \Delta\phi_t\right) \\ \lambda_t \sin\left(y_t(1) + \Delta\phi_t\right) \\ \Delta\phi_t \end{bmatrix}$$

Pseudocode ends

A new displacement $\left(\Delta\theta_{t+1}, \lambda_{t+1}\right)$ was then generated recursively starting from the new pose y_{t+1}.

For the random trajectory model, $\sigma_\tau = 0.5$, while for the thigmotactic bias trajectory model, $\sigma_\tau = 0.1$. The factor $(1.1)^k$ ensures that attempted turns gradually increase in magnitude while a viable next step has not been found. WN denotes a wrapped normal distribution. Note that the thigmotactic trajectory model variant is only suitable for convex arenas [7].

S1.2.2. Thigmotactic bias for learning in darkness

For learning trials in darkness (e.g., Fig 8A), a trajectory model with additional thigmotactic bias was used to ensure adequate learning of boundary contact regions (< 7 cm from the nearest boundary), which resulted in denser sampling of short-range boundary contact information.

There was an initial 2 minutes of pure thigmotaxis where the trajectory remained 2 cm from the boundary, moving anticlockwise. The remaining trajectory were 1-minute cycles consisting of a period of

random trajectory with thigmotactic bias ($\sigma_\tau = 0.1$) and a period of standard random trajectory ($\sigma_\tau = 0.5$), set empirically to maintain approximately uniform coverage of the traversable space.

S1.2.3. Hairpin maze

A path was constructed from straight segments along the center of the hairpin maze corridors (Fig 6A). Along each segment, the endpoint was used as a beacon to generate the noisy trajectory such that $\phi_t \sim \mathrm{WN}\left(\phi_t^{Beacon}, \sigma_\tau^2\right)$ where ϕ_t^{Beacon} was the true direction to the next beacon point at *t*. This algorithm was also used to generate the virtual hairpin paths (Fig 7C).

S1.2.4. Total trajectory length was decreased in darkness

The total trajectory length affects the spatial sampling density of the arena during learning and recall. Most published rat experiments were defined by the total time of each trial, which may have variable total trajectory length depending on movement speed. Rats typically run about 25% to 75% faster with vision during foraging [29, 36, 37]. This was modelled as an increase of 50% of the total number of steps per time period (relative to no vision), in all trials with vision. This increased the total trajectory length by 50% in trials with vision relative to no vision.

S1.3 Text. Data analysis

S1.3.1. Firing rate maps

Grid cell and boundary vector cell rate maps were calculated using the instantaneous activity following each step. Trajectories and corresponding unit activities were resampled 10× using linear interpolation to yield an effective sampling rate of 12.9Hz without vision and 19.3Hz with vision. For consistency with published studies, rate maps were calculated using three different spatial smoothing algorithms adapted from spike-based analyses used in the original studies. The average rate at bin location (*x*, *y*) is defined as

$$\overline{f_{rate}}(x,y) = \sum_t w_t f_{rate}(t) \tag{S35}$$

where $f_{rate}(t)$ is the instantaneous activity at step *t* and w_t denotes the weight which depends on the bin location, trajectory location at *t* and the smoothing kernel used. Since $0 \le f_{rate}(t) \le 1$ in the model **Error! Reference source not found.**, $0 \le \overline{f_{rate}}(x,y) \le 1$.

S1.3.1.1. Gaussian smoothing

Unless otherwise specified, a Gaussian smoothing kernel was used [9, 13, 33], defined by

$$w_t = \begin{cases} Exp\left(-\frac{(x-x_t)^2+(y-y_t)^2}{2\sigma_1^2}\right) & (x-x_t)^2+(y-y_t)^2 < r_1^2 \\ 0 & otherwise \end{cases} \tag{S36}$$

where $\sigma_1 = 2.5$ cm, $r_1 = 5$ cm and w_t was normalized so that $\sum w_t = 1$. Bins were centered on a 2.5 cm square grid.

S1.3.1.2. Boxcar smoothing

For the arena resizing experiments [32] a boxcar smoothing kernel was applied, i.e.,

$$w_t = \begin{cases} 1 & (x-x_t)^2 < r_1^2 \wedge (y-y_t)^2 < r_2^2 \\ 0 & otherwise \end{cases} \tag{S37}$$

where $r_1 = r_2 = 5\,\text{cm}$ and w_t was normalized so that $\sum w_t = 1$. Bins were centered on a 2 cm square grid.

S1.3.1.3. Hairpin maze smoothing

In hairpin maze experiments in which maze arms rates were displayed as linear tracks [34], the smoothing kernel was

$$w_t = \begin{cases} Exp\left(-\frac{(y-y_t)^2}{2\sigma_1^2}\right) & (y-y_t)^2 < r_1^2 \wedge (x-x_t)^2 < r_2^2 \\ 0 & otherwise \end{cases} \tag{S38}$$

where $\sigma_1 = 2.5\,\text{cm}$, $r_1 = 5\,\text{cm}$, $r_2 = 7.5\,\text{cm}$ to cover the width of the hairpin corridors, and w_t was normalized so that $\sum w_t = 1$. Bins were centered on a rectangular grid of horizontal scale 15 cm (centered along maze corridors) and vertical scale 2.5 cm. Note that the for the arm-arm rate map correlation analyses, activity was placed into contiguous non-overlapping 10 cm x 15 cm bins [34], equivalent to boxcar smoothing with $r_1 = 7.5\,\text{cm}$ and $r_2 = 5\,\text{cm}$.

S1.3.2. Spatial crosscorrelograms

To characterize the spatial response patterns of grid cells, spatial crosscorrelograms were computed from the smoothed rate maps of individual cells [38].

$$\rho(u,v) = \frac{\sum_{x,y\in\Gamma_1}\left(f_{rate1}(x,y) - \overline{f_{rate1}(\Gamma_1)}\right)\left(f_{rate2}(x-u,y-v) - \overline{f_{rate2}(\Gamma_2)}\right)}{\sqrt{\sum_{x,y\in\Gamma_1}\left(f_{rate1}(x,y) - \overline{f_{rate1}(\Gamma_1)}\right)\sum_{x,y\in\Gamma_2}\left(f_{rate2}(x-u,y-v) - \overline{f_{rate2}(\Gamma_2)}\right)}} \tag{S39}$$

where $\rho(u,v)$ is the crosscorrelation coefficient at bin position (u,v), f_{rate1} is the first rate map, f_{rate2} is the second rate map, Γ_1 is the region of f_{rate1} which overlaps with the region Γ_2 of f_{rate2} when f_{rate2} is translated by (u,v) relative to f_{rate1}, $\overline{f_{rate}(\Gamma)}$ is the mean of f_{rate} over the region Γ. Note that Γ_1 and Γ_2 are always equal in size and only those bins in both Γ_1 and Γ_2 which are defined are used, excluding unsampled spatial bins. When $f_{rate1} = f_{rate2}$, ρ is the autocorrelogram.

S1.3.3. Gridness index

The gridness index (also termed gridness score) was developed to quantify how well the activity patterns of putative grid cells match a regular hexagonal grid [9, 38-40]. Four gridness index calculations were performed on autocorrelograms using a fixed and variable radius circular annulus mask [32], and a fixed and variable radius elliptic annulus mask [38]. The autocorrelogram pixels under the mask were rotated by α = 30°, 60°, 90°, 120° and 150°, and the Pearson correlation, $r(\alpha)$, found with the unrotated pixels. The gridness index was calculated as

$$g = \min\left(r\left(60^\circ\right), r\left(120^\circ\right)\right) - \max\left(r\left(30^\circ\right), r\left(90^\circ\right), r\left(150^\circ\right)\right) \tag{S40}$$

To define a circular annulus, an average grid scale, $\bar{r}_g$, was first found from the distances to the six nearest autocorrelogram peaks excluding the central peak [32]. The inner and outer radius of a fixed circular annulus was defined as $\bar{r}_g/2$ and $3\bar{r}_g/2$, respectively. The inner and outer radius of a variable circular annulus was defined as $(1-k)\bar{r}_g$ and $(1+k)\bar{r}_g$, respectively, where $k \in \{0.02, 0.04, ..., 0.5\}$.

To define an elliptic annulus [38], an ellipse was fitted to the six nearest autocorrelogram peaks excluding the central peak, by finding the eigenvalues and eigenvectors of the second moment matrix of the peak coordinates. The elliptic axes were defined by the eigenvectors, with lengths $\sqrt{2d}$ where d are the corresponding eigenvalues.

S1.3.4. Grid rescaling

Grid cell rate maps were analyzed for the amount of joint rescaling along the two orthogonal dimensions of the resized rectangular boundaries [32]. Briefly, each smoothed rate map from the three resized arenas (test) was compared with the average of the two rate maps in the training arena (reference). Each test rate map was resampled using bilinear interpolation with the number of resampled bins varying independently along the X and Y dimensions. For example, a 100 cm × 70 cm test arena has a baseline smoothed rate map consisting of 50 × 35 bins (2 cm separation between bin centers). A total of 43 × 43 = 1,849 resampled rate maps were generated, using 29 to 71 bins along the 100 cm dimension, and 20 to 62 bins along the 70 cm dimension.

These resampled rate maps covered rescaling ratios of 50/71 ≈ 0.70 to 50/29 ≈ 1.72 along the 100 cm dimension, and 35/62 ≈ 0.56 to 35/20 ≈ 1.75 along the 70 cm dimension.

Next, the resampled rate map which had the highest correlation with the reference map was found. The number of bins along the resized dimension was then used to calculate the rescaling value. If grid spacing was unchanged, then rescaling would be 50/50 = 1.00 and 35/35 = 1.00 (since 50 × 35 bins was optimal). For instance, if the test grid partially contracted along the 70 cm dimension, so that 42 bins produced the optimal correlation between test and reference maps, rescaling would be 35/42 ≈ 0.83. If a test map consisted of the entire reference map of 100 cm, but completely squashed inside 70 cm, then 50 bins would produce the optimal correlation, resulting in a rescaling estimate of 35/50 ≈ 0.70, i.e., 100% rescaling. To account for the possibility that grid rescaling co-occurred with grid translation, the value of the center-most crosscorrelogram peak between a reference map and resampled test map was used (S39).

S1.3.5. Border score

The border score was developed to quantify how closely a cell's firing field follows an arena boundary [41]. Briefly, fields were defined as sets of neighboring bins in a rate map >0.3 of the maximum rate and >200cm^2 in area. Next, c_M was defined as the maximum fractional coverage of bins immediately adjacent to any of the four walls by any field. In circular arenas, c_M was defined as the maximum fractional coverage of bins immediately adjacent to any 90 degree arc. Next, d_M was defined as the average perpendicular distance to the nearest wall from all pixels of all fields, normalized by half the shortest side of the environment (radius for circular arenas). The border score was defined as

$$b = \frac{c_M - d_M}{c_M + d_M} \tag{S41}$$

and a 'border cell' was defined as a cell with b > 0.5 [41]. The border score was designed to measure elongation of a field parallel to a boundary coupled with compression of that field perpendicular to the boundary.

S1.3.6. Parametric rate map correlation

The border score penalizes distance away from a boundary and excludes a subpopulation of both rodent and model long-range boundary cells [23, 41]. To avoid exclusion of long-range boundary cells, a parametric rate map correlation method was developed. Two parametric rate map correlation coefficients were found for each rate map: using a boundary vector cell hypothesis and a simplified oscillatory interference grid cell hypothesis. The cell was considered to be functionally more similar to the underlying model which produced the higher parametric rate map correlation.

The boundary vector cell hypothesis states that activity *f* at each position (*x*, *y*) in a rate map is determined by distance *r* and allocentric direction *θ* of some boundary points. A complementary map in parameter space is constructed based on this hypothesis and the original rate map *f*. In practice, it is impossible to tell which boundary points actually contribute to *f*(*x*, *y*), so assume that they all contribute equally. Inversely, the firing rate *f*(*x*, *y*) is added to all sampled boundary (*r*, *θ*) pairs, i.e.,

$$h_b\left(r,\theta\right)=\sum_{x,y}n_b\left(x,y,r,\theta\right)\cdot f\left(x,y\right) \tag{S42}$$

where $n_b\left(x,y,r,\theta\right)$ is the number of boundary points whose polar vector from (*x*, *y*) lies within the polar bin centered at (*r*, *θ*). Here, $r\in\{2.5,5,...,100\}$ cm, and $\theta\in\{4,8,...,360\}$ degrees. To reduce overfitting of noisy features in the rate map, the parameter function h_b is smoothed with a boxcar kernel of width 5 × 5 bins, wrapping around in circular space (*θ* dimension of h_b). Using h_b, a new rate map f_b is then generated, i.e.,

$$f_b\left(x,y\right)=\sum_{r,\theta}n_f\left(r,\theta,x,y\right)\cdot h_b\left(r,\theta\right) \tag{S43}$$

where $n_f\left(r,\theta,x,y\right)$ denotes the number of boundary points whose polar vector from (*x*, *y*) lies within the Cartesian bin centered at (*x*, *y*). As above, $r\in\{2.5,5,...,100\}$ cm, and $\theta\in\{4,8,...,360\}$ degrees, while *x* and *y* bin centers match the original rate map *f*. Finally, the bin-wise Pearson correlation coefficient between *f* and f_b gives the parametric rate map correlation for a BVC hypothesis, $r\left(Hyp:BVC\right)$.

The simplified oscillatory interference grid cell hypothesis states that rate maps can be approximated by spatially-sinusoidal oscillations of various phases, frequencies and directions [12]. The resultant

parameter map is similar to a 2D Fourier spectrogram [42], but constructed using a marginal rate map generated along each direction tested, i.e.,

$$h_g(r,\theta) = \frac{1}{rN}\sum_{t=1}^{N} a_t Exp(-i\omega u_t) \tag{S44}$$

where $i = \sqrt{-1}$, $u_t = x_t \cos\theta + y_t \sin\theta$ is the projection of path position (x_t, y_t) along direction θ at step t, a_t is the response of the cell, $\omega = 2\pi/r$ is the spatial frequency being tested. One advantage of (S44) over a standard 2D Fourier spectrogram [42] is the natural reduction in weight given to positions along the θ-axis which are under-sampled due to arena geometry and/or trajectory inhomogeneity. To use a spike time series, $a_t = 1$ if there is a spike at step t, $a_t = 0$ otherwise. For consistency with h_b, h_g is also smoothed with a boxcar kernel of width 5 × 5 bins. Using h_g, a new activity vector a_t is then generated, i.e.,

$$a_t = \sum_{r,\theta} \frac{|h_g(r,\theta)|}{r^2} \cos\left(\omega u_t + Arg\left(h_g(r,\theta)\right)\right) \tag{S45}$$

where $|h_g(r,\theta)|$ and $Arg\left(h_g(r,\theta)\right)$ denote the complex modulus and argument of h_g respectively. From a_t, x_t and y_t, a new rate map $f_g(x,y)$ is calculated (in the same way that the original rate map $f(x,y)$ was calculated from a_t, x_t and y_t). Finally, the bin-wise Pearson correlation coefficient between f and f_g is the parametric rate map correlation for a simplified oscillatory grid cell hypothesis, $r(Hyp:GC)$.

Using forced choice classification, if $r(Hyp:BVC) > r(Hyp:GC)$, a cell was classified as a boundary cell. If $r(Hyp:BVC) < r(Hyp:GC)$, a cell was classified as a grid cell. This method can potentially be used with other hypotheses, either in addition to or in place of the ones described here. In S5 Fig, forced choice classification is supplemented by defining a cell as unclassified if both $r(Hyp:BVC) < 0.5$ and $r(Hyp:GC) < 0.5$, which suggests that neither hypothesis is adequate to approximate the spatial response characteristics of the cell. In addition to improving classification rate from standard metrics (tested using model cells whose generative mechanisms are unambiguous), the

reconstructed rate map provides an intuitive visual check as to whether the rate map properties of interest have been satisfactorily captured by the hypothesis (S5 Fig).

It is worth noting that using the border score, the average unclassified boundary cells per experiment ranged from 27% (learning in a 1 m circular arena) to 54% (recall in 70 cm × 100 cm rectangular arena, learning in 100 cm × 70 cm rectangular arena). The variability can be explained as follows. The SIFM boundary cell tuning function is based on the published BVC model whose optimal tuning depends on a bivariate Gaussian function of distance and direction, which maps to a crescentic function in Cartesian coordinates (Jacobian = 1/r [43]). Consequently, a curved boundary tends to result in fields with peak activity slightly closer to the boundary (boundary geometry more closely matches the tuning function). The latter results in a lower normalized perpendicular wall distance as defined in the border score, increasing the fraction of boundary cells whose fields reach border score criterion. In resized arenas, predictive boundary fields stretched or compressed according to arena resizing. This led to a significant portion of boundary fields which widened along the perpendicular dimension, which reduced the border score. Fields were also noisier in resized arenas which further reduced the border score.

S1.3.7. Visualizing associative weight maps

Learned spatial information in SIFM is stored in associative weights between grid codes and boundary codes, and visualized by averaging the associative weights between all boundary vector cells and each map grid position, and collapsing across all grid codes, i.e.,

$$\overline{W}_m^{[k]} = \frac{1}{N}\sum_{i,j} W_t^{[i,k,j]} \tag{S46}$$

where $\overline{W}_m^{[k]}$ denotes the map associative weight for map element [*k*], *N* is the number of grid codes multiplied by the number of boundary vector cells, and $W_t^{[i,k,j]}$ is the associative weight between map element [*k*] and boundary cell [*j*] of grid code [*i*]. The resulting associative weight at each map element can be interpreted as the total boundary cell activity expected at that grid code. Partial weight maps are similarly produced using only long-range boundary cells, or only short-range boundary cells (e.g., Fig 2). Since a grid

code does not correspond uniquely to an (*x*, *y*) position, the coordinate used to display associative weights is the ideal (*x*, *y*) used to generate the map grid code reference during initialization of a learning trial.

S1.3.8. Simulated spikes

Using linearly interpolated positions and instantaneous rate, $f_{rate}(t)$, spikes were generated using a stochastic model for visual comparison with published data. All analyses of simulated grid and boundary cells were performed on the instantaneous rate $f_{rate}(t)$ unless otherwise specified. Spikes were simulated according to

$$s(t) = \begin{cases} 1 & f_{rate}(t) > r(t)/\lambda_{spike} \\ 0 & otherwise \end{cases} \tag{S47}$$

where $s(t)=1$ denotes a spike at time *t*, $s(t)=0$ denotes no spike at time *t*, the random vector $r(t) \sim U(0,1)$, and $0.5 \le \lambda_{spike} \le 1$. The Poisson spike rate constant λ_{spike} was manually adjusted to vary the total spike count for visual clarity, and to be similar to rat data when comparisons are shown. For example, if $f_{rate}(t)=1$, $\lambda_{spike}=0.5$, the expected number of spikes at *t* is $\langle s(t) \rangle = p(\lambda_{spike} f_{rate}(t) > r(t)) = 0.5$. Since $0 \le f_{rate}(t) \le 1$ and $r(t) \sim U(0,1)$,

$$\langle s(t) \rangle = \lambda_{spike} f_{rate}(t) \tag{S48}$$

as long as $\lambda_{spike} \le 1$, producing an average stochastic spike rate proportional to $f_{rate}(t)$.

S1.3.9. Short-range boundary vector maps

A short-range boundary cell responds maximally when its tuning direction aligns with a boundary normal because the widest extent of the boundary contributes to its response (S22). Therefore, the population activity of predictive short-range boundary cells reflect the local boundary normal which has been learned. At each spatial bin, the circular mean direction of short-range boundary cells is found, weighted by the boundary cell's mean activity in that bin. The weighted circular mean direction is then displayed according to a color wheel (e.g., Fig 4B lower right), and whose intensity scales linearly with the weighted circular

concentration (mean resultant length) [44]. For example, bright green indicates a westerly mean boundary vector with low variance (concentration approaching 1), while dark red indicates an easterly mean boundary vector with high variance (concentration approaching 0). This color-coded boundary vector map summarizes location-dependent arena boundary orientation information encoded by a population of predictive boundary cells, despite individual cells having no directional information content (Fig 2).

S1.3.10. Dynamic grid cell rate maps and autocorrelograms

Standard rate maps and autocorrelograms are suited for analyzing static tessellating spatial response patterns. However, if there is spatial drift in the response pattern, such as due to cumulative error during PI, then a static reference frame may smear out any underlying regularity. In contrast, a dynamically-referenced response map can be used to enhance drifting but otherwise stable spatial patterns in the response [10]. To produce a dynamic grid cell rate map, spikes were generated stochastically using the SIFM activity response time series as described previously (S1.3.8 Text). Each spike in a trial was used as reference. The relative spike positions were plotted for spikes within a 10-s time window after each reference spike, forming a dynamic rate map pooled over all reference spikes. A dynamic autocorrelogram was calculated from the dynamic rate map as for static rate maps and autocorrelograms (S1.3.2 Text).

S1.3.11. Grid phase change

Grid phase can change across trials, even within the same arena and without further learning (S9 Fig). Normalized phase shift quantification was adapted from the Online Methods of [38]. Briefly, grid parameters $(\lambda_1, \lambda_2, \theta, \psi)$ were fitted to a crosscorrelogram (e.g., Fig 5, S9 Fig) and the raw phase shift was estimated as the center-most crosscorrelogram peak position (r_c, ϕ_c) expressed in polar coordinates. The normalized phase shift components were found by projecting the raw phase shift onto the two fitted grid lattice axes, and scaled by their magnitudes, i.e.,

$$x_1^{'} = \left(\frac{r_c \sin(\psi + \theta - \phi_c)}{\lambda_1 \sin\theta} \right) \mod 1 \tag{S49}$$

$$x_2^{'} = \left(\frac{r_c \sin\left(\phi_c - \psi\right)}{\lambda_2 \sin\theta} \right) \bmod 1 \tag{S50}$$

Finally, to avoid phase shifts which are close to zero being inappropriately mapped to four corners of the unit rhombus lattice, the following remapping was applied:

$$\left(x_1, x_2\right) = \begin{cases} \left(x_1^{'} - 1, x_2^{'} - 1\right) & x_1^{'} \geq 0.5 \wedge x_2^{'} \geq 0.5 \\ \left(x_1^{'} - 1, x_2^{'}\right) & x_1^{'} \geq 0.5 \wedge x_2^{'} < 0.5 \\ \left(x_1^{'}, x_2^{'} - 1\right) & x_1^{'} < 0.5 \wedge x_2^{'} \geq 0.5 \\ \left(x_1^{'}, x_2^{'}\right) & x_1^{'} < 0.5 \wedge x_2^{'} < 0.5 \end{cases} \tag{S51}$$

Three types of phase changes were estimated in this way, using the nomenclature of [38]. The first is the phase shift of a single grid cell across two trials within the same arena, i.e., $\left(x_1, x_2\right) = \left(\Delta_t\left(\phi_1^{\alpha}\right), \Delta_t\left(\phi_2^{\alpha}\right)\right)$ where $\Delta_t\left(\phi_j^{\alpha}\right)$ denotes the normalized phase shift of the same cell along grid lattice *j*. The second is the phase offset between two grid cells within the same trial, i.e., $\left(x_1, x_2\right) = \left(\delta_1^{\alpha\beta}, \delta_2^{\alpha\beta}\right)$ where $\delta_j^{\alpha\beta}$ denotes the normalized phase offset between two cells along grid lattice *j*. Note that SIFM grid cells in this work are phase matched so the ideal noise-free grid offset is zero. Third, the change in the phase offset between a pair of grid cells is calculated across two trials within the same arena, i.e., $\left(x_1, x_2\right) = \left(\Delta_t\left(\delta_1^{\alpha\beta}\right), \Delta_t\left(\delta_2^{\alpha\beta}\right)\right)$ where $\Delta_t\left(\delta_j^{\alpha\beta}\right)$ denotes the normalized change in phase offset between a pair of cells along grid lattice *j*. Since the ideal SIFM grid axes are separated by 60°, both for simplicity and following the analysis of [38], the normalized radial magnitude of each phase change is given by

$$Magnitude = \sqrt{\left(x_2 \sin\left(60°\right)\right)^2 + \left(x_1 + x_2 \cos\left(60°\right)\right)^2} = \sqrt{x_1^2 + x_2^2 + x_1 x_2} \tag{S52}$$

S4. Supporting References

1. Murphy K. Bayesian map learning in dynamic environments. Neural Info Proc Systems (NIPS); Denver, CO, USA1999.
2. Montemerlo M, Thrun S, Koller D, Wegbreit B. FastSLAM: A factored solution to the simultaneous localization and mapping problem. Eighteenth National Conference on Artificial Intelligence (Aaai-02)/Fourteenth Innovative Applications of Artificial Intelligence Conference (Iaai-02), Proceedings. 2002:593-8. PubMed PMID: WOS:000183593700089.
3. Stachniss C, Hahnel D, Burgard W, Grisetti G. On actively closing loops in grid-based FastSLAM. Adv Robotics. 2005;19(10):1059-79. doi: 10.1163/156855305774662181. PubMed PMID: WOS:000233920400003.
4. Pearson MJ, Fox C, Sullivan JC, Prescott TJ, Pipe T, Mitchinson B. Simultaneous localisation and mapping on a multi-degree of freedom biomimetic whiskered robot. 2013 Ieee International Conference on Robotics and Automation (Icra). 2013:586-92. PubMed PMID: WOS:000337617300086.
5. Thrun S, Burgard W, Fox D. Probabilistic Robotics. Cambridge: MIT Press; 2005. 652 p.
6. Cheung A, Ball D, Milford M, Wyeth G, Wiles J. Maintaining a cognitive map in darkness: the need to fuse boundary knowledge with path integration. PLoS computational biology. 2012;8(8):e1002651. Epub 2012/08/24. doi: 10.1371/journal.pcbi.1002651. PubMed PMID: 22916006; PubMed Central PMCID: PMC3420935.
7. Cheung A. Estimating Location without External Cues. PLoS computational biology. 2014;10(10):e1003927. doi: 10.1371/journal.pcbi.1003927. PubMed PMID: 25356642.
8. Stensola H, Stensola T, Solstad T, Froland K, Moser MB, Moser EI. The entorhinal grid map is discretized. Nature. 2012;492(7427):72-8. doi: 10.1038/Nature11649. PubMed PMID: ISI:000311893400047.
9. Sargolini F, Fyhn M, Hafting T, McNaughton BL, Witter MP, Moser MB, et al. Conjunctive representation of position, direction, and velocity in entorhinal cortex. Science. 2006;312(5774):758-62. Epub 2006/05/06. doi: 10.1126/science.1125572. PubMed PMID: 16675704.
10. Bonnevie T, Dunn B, Fyhn M, Hafting T, Derdikman D, Kubie JL, et al. Grid cells require excitatory drive from the hippocampus. Nat Neurosci. 2013;16(3):309-17. doi: 10.1038/nn.3311. PubMed PMID: 23334581.
11. Krupic J, Bauza M, Burton S, Barry C, O'Keefe J. Grid cell symmetry is shaped by environmental geometry. Nature. 2015;518(7538):232-5. doi: 10.1038/nature14153. PubMed PMID: 25673417.
12. Burgess N, Barry C, O'Keefe J. An oscillatory interference model of grid cell firing. Hippocampus. 2007;17(9):801-12. doi: 10.1002/hipo.20327. PubMed PMID: ISI:000249423700010.
13. Hafting T, Fyhn M, Molden S, Moser MB, Moser EI. Microstructure of a spatial map in the entorhinal cortex. Nature. 2005;436(7052):801-6. Epub 2005/06/21. doi: 10.1038/nature03721. PubMed PMID: 15965463.
14. Hasselmo ME, Giocomo LM, Zilli EA. Grid cell firing may arise from interference of theta frequency membrane potential oscillations in single neurons. Hippocampus. 2007;17(12):1252-71. doi: 10.1002/hipo.20374. PubMed PMID: 17924530; PubMed Central PMCID: PMC2408670.
15. Burgess N. Grid cells and theta as oscillatory interference: theory and predictions. Hippocampus. 2008;18(12):1157-74. doi: 10.1002/hipo.20518. PubMed PMID: 19021256; PubMed Central PMCID: PMC3196519.
16. Goodridge JP, Dudchenko PA, Worboys KA, Golob EJ, Taube JS. Cue control and head direction cells. Behavioral neuroscience. 1998;112(4):749-61. Epub 1998/09/11. PubMed PMID: 9733184.
17. Clark BJ, Taube JS. Intect landmark control and angular path integration by head direction cells in the anterodorsal thalamus after lesions of the medial entorhinal cortex. Hippocampus. 2011;21(7):767-82. Epub 3 Nov 2010. doi: 10.1002/hipo.20874.
18. Sreenivasan S, Fiete I. Grid cells generate an analog error-correcting code for singularly precise neural computation. Nat Neurosci. 2011;14(10):1330-7. doi: 10.1038/nn.2901. PubMed PMID: 21909090.
19. Bush D, Barry C, Manson D, Burgess N. Using grid cells for navigation. Neuron. 2015;87:507-20. doi: 10.1016/j.neuron.2015.07.006.
20. O'Keefe J, Burgess N. Geometric determinants of the place fields of hippocampal neurons. Nature. 1996;381(6581):425-8. Epub 1996/05/30. doi: 10.1038/381425a0. PubMed PMID: 8632799.
21. Hartley T, Burgess N, Lever C, Cacucci F, O'Keefe J. Modeling place fields in terms of the cortical inputs to the hippocampus. Hippocampus. 2000;10(4):369-79. doi: 10.1002/1098-1063(2000)10:4<369::Aid-Hipo3>3.0.Co;2-0. PubMed PMID: ISI:000089292000003.
22. Barry C, Lever C, Hayman R, Hartley T, Burton S, O'Keefe J, et al. The boundary vector cell model of place cell firing and spatial memory. Rev Neurosci. 2006;17(1-2):71-97. PubMed PMID: 16703944; PubMed Central PMCID: PMC2677716.
23. Lever C, Burton S, Jeewajee A, O'Keefe J, Burgess N. Boundary vector cells in the subiculum of the hippocampal formation. The Journal of neuroscience : the official journal of the Society for Neuroscience. 2009;29(31):9771-7. Epub 2009/08/07. doi: 10.1523/JNEUROSCI.1319-09.2009. PubMed PMID: 19657030; PubMed Central PMCID: PMC2736390.
24. Bjerknes TL, Moser EI, Moser MB. Representation of geometric borders in the developing rat. Neuron. 2014;82(1):71-8. doi: 10.1016/j.neuron.2014.02.014. PubMed PMID: 24613417.
25. Ibrahim L, Wright EA. The growth of rats and mice vibrissae under normal and some abnormal conditions. Journal of embryology and experimental morphology. 1975;33(4):831-44. PubMed PMID: 1176877.
26. Raudies F, Hasselmo ME. Modeling boundary vector cell firing given optic flow as a cue. PLoS computational biology. 2012;8(6):e1002553. doi: 10.1371/journal.pcbi.1002553. PubMed PMID: 22761557; PubMed Central PMCID: PMC3386186.
27. Adams AD, Forrester JM. The projection of the rat's visual field on the cerebral cortex. Q J Exp Physiol Cogn Med Sci. 1968;53(3):327-36. PubMed PMID: 5188317.
28. Fanselow EE, Nicolelis MA. Behavioral modulation of tactile responses in the rat somatosensory system. The Journal of neuroscience : the official journal of the Society for Neuroscience. 1999;19(17):7603-16. PubMed PMID: 10460266.

29. Save E, Cressant A, Thinus-Blanc C, Poucet B. Spatial firing of hippocampal place cells in blind rats. The Journal of neuroscience : the official journal of the Society for Neuroscience. 1998;18(5):1818-26. PubMed PMID: ISI:000072092600020.
30. Taube JS, Bassett JP. Persistent neural activity in head direction cells. Cerebral cortex. 2003;13(11):1162-72. Epub 2003/10/25. PubMed PMID: 14576208.
31. van Leeuwen PJ. Particle filtering in geophysical systems. Mon Wea Rev. 2009;137:4089-114.
32. Barry C, Hayman R, Burgess N, Jeffery KJ. Experience-dependent rescaling of entorhinal grids. Nat Neurosci. 2007;10(6):682-4. doi: 10.1038/nn1905. PubMed PMID: WOS:000246799800008.
33. Fyhn M, Hafting T, Treves A, Moser MB, Moser EI. Hippocampal remapping and grid realignment in entorhinal cortex. Nature. 2007;446(7132):190-4. doi: 10.1038/nature05601. PubMed PMID: 17322902.
34. Derdikman D, Whitlock JR, Tsao A, Fyhn M, Hafting T, Moser MB, et al. Fragmentation of grid cell maps in a multicompartment environment. Nat Neurosci. 2009;12(10):1325-32. doi: 10.1038/nn.2396. PubMed PMID: 19749749.
35. Stensola T, Stensola H, Moser MB, Moser EI. Shearing-induced asymmetry in entorhinal grid cells. Nature. 2015;518(7538):207-12. doi: 10.1038/nature14151. PubMed PMID: 25673414.
36. Hruska RE, Kennedy S, Silbergeld EK. Quantitative aspects of normal locomotion in rats. Life sciences. 1979;25(2):171-9. PubMed PMID: 491843.
37. Berryman ER, Harris RL, Moalli M, Bagi CM. Digigait quantitation of gait dynamics in rat rheumatoid arthritis model. Journal of musculoskeletal & neuronal interactions. 2009;9(2):89-98. PubMed PMID: 19516084.
38. Yoon K, Buice MA, Barry C, Hayman R, Burgess N, Fiete IR. Specific evidence of low-dimensional continuous attractor dynamics in grid cells. Nat Neurosci. 2013;16(8):1077-84. doi: 10.1038/nn.3450. PubMed PMID: 23852111; PubMed Central PMCID: PMC3797513.
39. Savelli F, Yoganarasimha D, Knierim JJ. Influence of boundary removal on the spatial representations of the medial entorhinal cortex. Hippocampus. 2008;18(12):1270-82. doi: 10.1002/hipo.20511. PubMed PMID: 19021262; PubMed Central PMCID: PMC3007674.
40. Langston RF, Ainge JA, Couey JJ, Canto CB, Bjerknes TL, Witter MP, et al. Development of the Spatial Representation System in the Rat. Science. 2010;328(5985):1576-80. doi: 10.1126/science.1188210. PubMed PMID: WOS:000278859200052.
41. Solstad T, Boccara CN, Kropff E, Moser MB, Moser EI. Representation of geometric borders in the entorhinal cortex. Science. 2008;322(5909):1865-8. Epub 2008/12/20. doi: 10.1126/science.1166466. PubMed PMID: 19095945.
42. Krupic J, Burgess N, O'Keefe J. Neural representations of location composed of spatially periodic bands. Science. 2012;337(6096):853-7. doi: 10.1126/science.1222403. PubMed PMID: 22904012.
43. Cheung A, Zhang S, Stricker C, Srinivasan MV. Animal navigation: the difficulty of moving in a straight line. Biological cybernetics. 2007;97(1):47-61. Epub 2007/05/24. doi: 10.1007/s00422-007-0158-0. PubMed PMID: 17520273.
44. Berens P. CircStat: A MATLAB Toolbox for Circular Statistics. J Stat Softw. 2009;31(10):1-21. PubMed PMID: WOS:000270513700001.
