# Supplementary material for: Probabilistic Learning by Rodent Grid Cells
Source: PLoS Comput Biol. 2016 Oct 28;12(10):e1005165. doi: 10.1371/journal.pcbi.1005165 (PMC5085080; doi:10.1371/journal.pcbi.1005165)
Supplement: S1 Table — Gridness index (mean ± SD) of probabilistic grid cells following learning with (+) and without (-) compensatory phase noise in 1 m arenas. See S1.3.3 Text for definitions of the four variants of the gridness index used. (DOC) [file pcbi.1005165.s014.doc]

| **Arena**  **Gridness** | **Square (+)** | **Square (-)** | **Circle (+)** | **Circle (-)** |
| --- | --- | --- | --- | --- |
| **Standard** | 1.93 ± 0.05 | 0.26 ± 0.55 | 1.91 ± 0.07 | 0.52 ± 0.51 |
| **Modified** | 1.95 ± 0.04 | 0.26 ± 0.56 | 1.94 ± 0.05 | 0.46 ± 0.53 |
| **Standard**  **(fixed annulus)** | 1.23 ± 0.07 | -0.05 ± 0.27 | 1.17 ± 0.11 | 0.09 ± 0.34 |
| **Modified**  **(fixed annulus)** | 1.26 ± 0.07 | -0.10 ± 0.29 | 1.20 ± 0.09 | 0.10 ± 0.30 |

**Table S1. Stable grids require phase noise.** Gridness index (mean ± SD) of probabilistic grid cells following learning with (+) and without (-) phase noise in 1 m arenas. See Text S3.3 for definitions of the four variants of the gridness index used.
