# Supplementary material for: Probabilistic Learning by Rodent Grid Cells
Source: PLoS Comput Biol. 2016 Oct 28;12(10):e1005165. doi: 10.1371/journal.pcbi.1005165 (PMC5085080; doi:10.1371/journal.pcbi.1005165)
Supplement: S2 Table — Correlation between population arm-arm correlation matrices from probabilistic and rat grid cells in a 1.5 m hairpin maze (excludes main diagonal of arm-arm correlation matrices). (DOC) [file pcbi.1005165.s015.doc]

| **Direction**  **Condition** | **Probabilistic easterly**  **vs**  **rat easterly** | | **Probabilistic westerly**  **vs**  **rat westerly** | | **Probabilistic easterly**  **vs**  **rat westerly** | | **Probabilistic westerly**  **vs**  **rat easterly** | |
| --- | --- | --- | --- | --- | --- | --- | --- | --- |
|  | *r* | *P* | *r* | *P* | *r* | *P* | *r* | *P* |
|  |  |  |  |  |  |  |  |  |
| **Standard** | 0.92 | 9.9 × 10^-43^ | 0.95 | 1.8 × 10^-50^ | 0.82 | 2.0 × 10^-25^ | 0.86 | 4.2 × 10^-31^ |
| **4-fold map resolution** | 0.93 | 1.0 × 10^-43^ | 0.94 | 1.3 × 10^-47^ | 0.86 | 9.0 × 10^-30^ | 0.83 | 6.9 × 10^-27^ |
| **0.25-fold map resolution** | 0.91 | 4.6 × 10^-39^ | 0.86 | 1.0 × 10^-29^ | 0.69 | 3.6 × 10^-15^ | 0.82 | 4.8 × 10^-25^ |
| **4-fold noise variances** | 0.92 | 1.5 × 10^-42^ | 0.93 | 1.9 × 10^-43^ | 0.83 | 1.1 × 10^-26^ | 0.85 | 3.1 × 10^-29^ |
| **0.25-fold noise variances** | 0.52 | 2.5 × 10^-8^ | 0.27 | 7.2 × 10^-3^ | 0.26 | 7.8 × 10^-3^ | 0.41 | 1.9 × 10^-5^ |

**Table S2. Grid fragmentation caused by self-motion noise.** Correlation between population arm-arm correlation matrices from probabilistic and rat grid cells in a 1.5 m hairpin maze (excludes main diagonal of arm-arm correlation matrices).
